# Comparative time-series multi-omics analyses suggest H1.2 involvement in anoxic adaptation and cancer resistance

**DOI:** 10.1371/journal.pbio.3002778

**Published:** 2024-08-23

**Authors:** Juan Du, Weiqiang Liu, Meng Li, Zihao Li, Xuanjing Li, Yichen Dai, Gaoming Liu, Xiao Wang, Pingfen Zhu, Vadim N. Gladyshev, Xuming Zhou

**Affiliations:** 1 CAS Key Laboratory of Animal Ecology and Conservation Biology, Institute of Zoology, Chaoyang District, Beijing, China; 2 University of Chinese Academy of Sciences, Beijing, China; 3 Division of Genetics, Department of Medicine, Brigham and Women’s Hospital and Harvard Medical School, Boston, Massachusetts, United States of America; Duke University, UNITED STATES OF AMERICA

## Abstract

The naked mole rat (NMR), *Heterocephalus glaber*, is known as the longest-lived rodent and is extraordinarily resistant to hypoxia and cancer. Here, both NMR embryonic fibroblasts (NEFs) and their mouse counterparts (MEFs) were subjected to anoxic conditions (0% O_2_, 5% CO_2_). A combination of comparative transcriptomics and proteomics was then employed to identify differentially expressed genes (DEGs). Notably, we observed distinct levels of histone H1.2 (encoded by *HIST1H1C*) accumulation between NEFs and MEFs. Subsequent mechanistic analyses showed that higher H1.2 expression in NEFs was associated with the lower expression of its inhibitor, *PARP1*. Additionally, we discovered that H1.2 can directly interact with HIF-1α PAS domains, thereby promoting the expression of HIF-1α through facilitating the dimerization with HIF-1β. The overexpression of H1.2 was also found to trigger autophagy and to suppress the migration of cancer cells, as well as the formation of xenograft tumors, via the NRF2/P62 signaling pathway. Moreover, an engineered H1.2 knock-in mouse model exhibited significantly extended survival in hypoxic conditions (4% O_2_) and showed a reduced rate of tumor formation. Collectively, our results indicate a potential mechanistic link between H1.2 and the dual phenomena of anoxic adaptation and cancer resistance.

## Abstract 2

Some long-lived animals, like the naked mole rat, are extraordinarily resistant to hypoxia and cancer. These authors show that embryonic fibroblasts from these animals overexpress histone 1.2, reducing tumor formation via the NRF2/P62 pathway and improving resistance to hypoxia.

## Introduction

Naked mole rats (NMRs), *Heterocephalus glaber*, are native to Eastern Africa and known for their extremely long lifespan, which reaches more than 37 years in captivity and 17 years in the wild [1] and is around 5-fold greater than their predicted lifespan according to body mass [2]. In addition to their longevity, NMRs show exceptional resistance to hypoxia and cancer, making them an excellent model for understanding genetic adaptations to hypoxia tolerance and cancer resistance.

NMRs reduce their basal metabolic rates to adapt to hypoxic environments [3]. Compared with mice, the NMR brain retains more of the N-methyl-D-aspartate receptor subunit GluN2D, which is protective during hypoxia [4]. In addition, NMR brain tissue is remarkably resistant to hypoxia, e.g., NMR neurons maintain synaptic transmission for longer periods than those of mice and can recover from >30 min of hypoxic treatment [5]. NMRs can also adapt to low oxygen environments using their unique physiological characteristics [6]. For example, they express high levels of hemoglobin α (Hba1 and Hba2) and neuroglobin (Ngb) [7]. Remarkably, NMRs can tolerate 18 min of total oxygen deprivation (anoxia), >60 min under 3% O_2_ conditions, and days to weeks under 8% O_2_ conditions without apparent injury [8–10]. During anoxia, NMRs switch to anaerobic metabolism fueled by fructose, which is actively accumulated and metabolized into lactate in the brain [10]. This fuel switch provides NMRs with an additional mode of ATP production under reduced oxygen, although metabolic rate must still be reduced to maintain energy balance.

NMRs also show an unusual resistance to cancer; indeed, studied animals had not shown the development of spontaneous neoplasms until 2 cases of tumors were reported in 2016 [11]. Researchers are currently seeking to uncover the genetic factors that underlie this unique resistance. For example, a study of NMR cells showed that, compared with human and mouse cells, they express a unique INK4 subtype, pALT^INK4a/b^, which is composed of the first exon of p15^INK4b^ and the second and third exons of p16^INK4a^; compared with p15^INK4b^ and p16^INK4a^, pALT^INK4a/b^ inhibits cell growth more effectively and may contribute to tumor resistance in NMRs [12]. In addition, NMR fibroblasts have been found to secrete high molecular weight hyaluronic acid (HMM-HA) and removing HMM-HA induces susceptibility to malignant transformation in NMR cells [13]. HMM-HA has also been shown to enhance cellular stress resistance in a p53-dependent manner [2], which is interesting because P53 is highly expressed in NMRs and has a long half-life in the nucleus and cytoplasm [14]. Other genetic changes have been implicated in the cancer resistance of NMRs, although validating evidence is required in these cases. For example, gene family analysis has revealed that the NMR genome contains 17 copies of the *PTEN* pseudogene and has suggested that the abnormal expression of this gene may contribute to NMR tumor resistance [15]. Interestingly, a characteristic of solid tumors is hypoxia, which leads to the malignant consequences of cancer [16]. Due to the increased consumption of oxygen that results from rapid cellular proliferation, hypoxia is a common stress in tumor progression. For example, cells closer to blood vessels had higher oxygen content and rapid multiplication, whereas cancer cells were usually located at 100 to 200 μm of functional blood vessels and/or close to the necrotic area that with very low oxygen content [17]. It is also found that hypoxia (1% O_2_) provided protection against acidosis-induced cell death compared to normoxia. At the same time, severe hypoxia (0.1% O_2_) could eliminate this protection, exacerbating acidosis-induced cell death in some cases [18]. However, in the hypoxic tumor microenvironment, cancer stem cells show enhanced activated differentiation potential [19]. Therefore, the relationship between hypoxia and tumor tissue microenvironment is complex, enabling us to explore the intrinsic relationship between hypoxic adaptation and cancer resistance in NMRs.

Integrative omics, i.e., a combination of omics approaches (e.g., genomics, transcriptomics, and proteomics), has been widely used to identify the driving factors in complex phenotypes. For example, single-nucleus RNA sequencing (snRNA-seq) and the single nucleus assay for transposase-accessible chromatin (snATAC-seq) have been employed previously to investigate tumor complexity [20]. Additionally, integrated snRNA-seq and snATAC-seq data have been used to determine cell type-specific markers that display cell type-specific patterns of chromatin accessibility [21]. In the present study, to explore the differences in cellular response to hypoxia in NMRs and mice, a combination of comparative transcriptomics and proteomics was employed to determine the gene expression under hypoxic condition. We aimed to characterize gene expression patterns in NMRs and mice according to their cellular response to hypoxia and identify any new genetic and epigenetic elements involved in the hypoxic tolerance of these animals.

## Results

### Multi-omics analysis reveals regions differentially expressed between NMR and mouse embryonic fibroblasts

**Fig 1:**
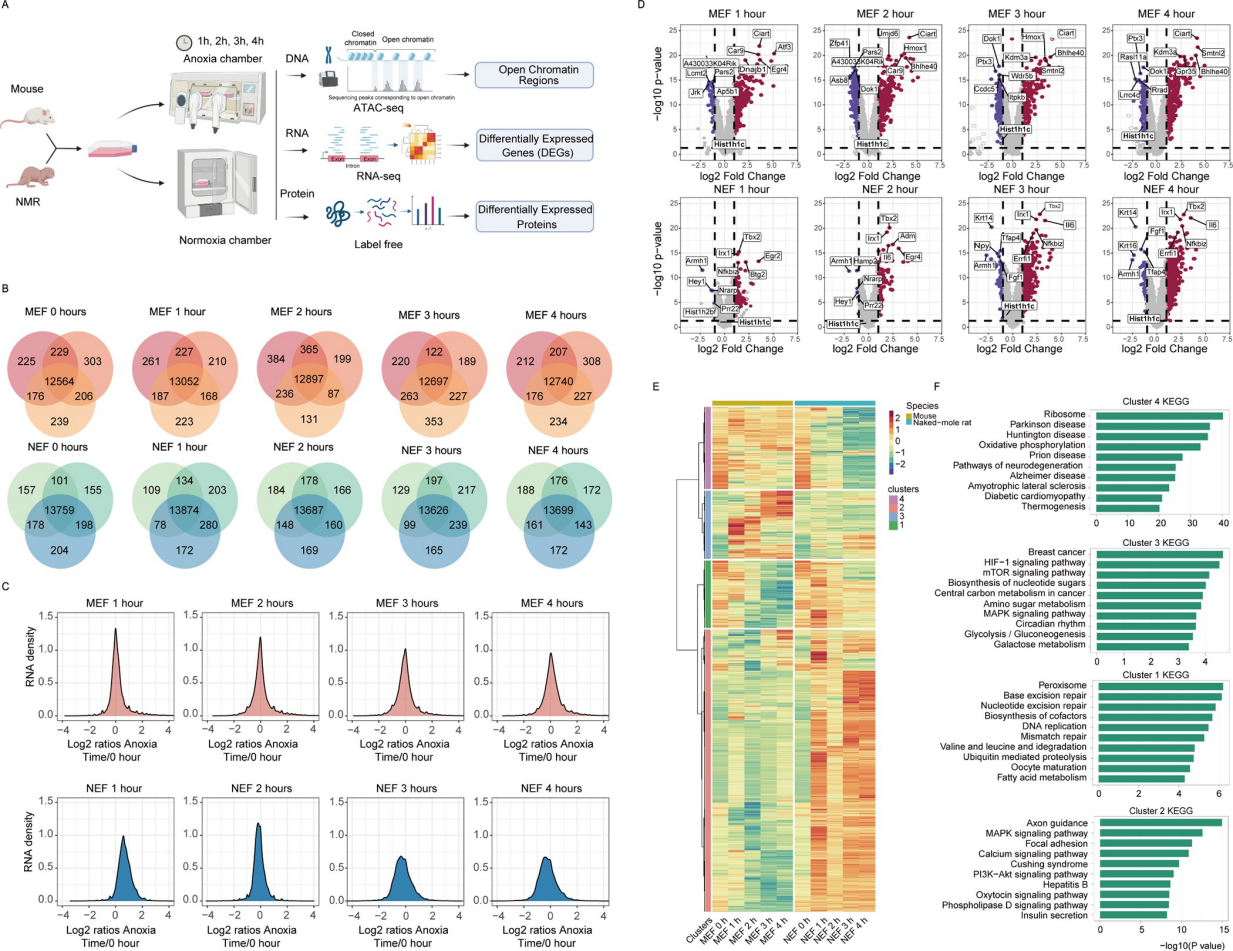
Integrative omics analysis of NMR and mouse fibroblasts under anoxic treatment. **(A)** Temporal response to anoxia in NEFs and MEFs across a 1–4-h O_2_-deprivation time course and analyses of NEFs and MEFs samples by label-free proteomics, RNA-seq, and ATAC-seq. For proteomics, proteins were extracted and digested using trypsin, and the resulting peptides were analyzed using LC-MS/MS label-free quantitative proteomics. For RNA-seq, total RNA was extracted, and cDNA was prepared and subjected to deep sequencing. For ATAC-seq, cells were digested with trypsin, and accessible DNA regions were identified by probing open chromatin with hyperactive mutant Tn5 transposase, which inserts sequencing adapters into the open regions of the genome. This panel is created with BioRender.com. **(B)** Venn diagram showing the number of identified mRNAs in NEFs and MEFs for the three biological replicates at each time point. **(C)** RNA density plots showing the distribution of log_2_ ratios for each time point relative to normoxia for both NEFs and MEFs. **(D)** Volcano plot of the identified mRNAs for each time point in NEFs and MEFs. Each point represents difference in the expression (fold change) relative to normoxia (time = 0) plotted against the level of statistical significance (*q* values). Different colors indicate density of data points. **(E)** Heatmap illustrating temporal changes between NEFs and MEFs at 1-, 2-, 3-, and 4-h time points under anoxic conditions. Genes were divided into 4 groups based on the relative expression under anoxic conditions compared with that under normoxic conditions. **(F)** KEGG pathways for each cluster. *X*-axis represents enrichment and the size depicts the log_10_ (*p* value) of each pathway; *y*-axis represents the different KEGG pathways. Data were scaled across rows before mapping to colors. The data underlying the graphs shown in the figure can be found in S1 Data. MEF, mouse embryonic fibroblast; NEF, NMR embryonic fibroblast; NMR, naked mole rat.

Considering the demonstrated fidelity of NMR fibroblasts in reflecting the species’ innate characteristics and their efficacy in molecular assays [13,22,23], we have selected NMR embryonic fibroblasts (NEFs) as our model to investigate the cellular response to anoxia in NMRs. In particular, NEFs and, for comparison, mouse embryonic fibroblasts (MEFs) were subjected to oxygen deprivation for 4 h (**Fig 1A**), whereas MEFs reach their maximum death (**S1 Fig**). Samples were harvested in triplicate at 5 time points (0, 1, 2, 3, and 4 h), and the samples were subjected to label-free mass spectrometry (MS)-based proteomics (*n* = 15) and RNA-seq-based transcriptomics (*n* = 14) (see **Method**, **Figs 1B–1D and S1–S4**). Pearson correlation analyses of RNA-seq and proteomics data showed high reproducibility among the samples harvested at similar time points (*r* > 0.93), and the correlation was higher at early time points during anoxia compared with the 4-h time point (**S2 Fig and S1 Table**). In general, housekeeping genes (e.g., *Hsp90ab1* and *Hspbp1*) showed stable expression across all time points, whereas expression of known hypoxia-related genes was highly dynamic and time dependent. For example, *Dst*, *Cyb5a*, and *Ddx47* were up-regulated in NEFs after 4 h of anoxic treatment, whereas *Spp1*, *Txnl1*, and *Pxdn* were down-regulated at this time point (**S1 Table**).

Using differential expression analysis, 12,138 and 9,335 differentially expressed genes (DEGs) in NEFs and MEFs were identified, respectively, which were differentially expressed at 1 time point or more (FDR < 0.05; **S1 Table**). In NEFs, more DEGs were up-regulated at 4 h compared with their expression in the first 3 h; in contrast, in MEFs, more DEGs were down-regulated at 4 h compared with their expression during the first 3 h. Hierarchical clustering was performed to determine clusters of genes with similar expression profiles. Accordingly, 4 distinct abundance profiles (clusters I–IV) in NEFs and MEFs (**Figs 1E, 1F, and S4**) were identified. Genes in cluster I (a group of genes showing little or no increase in abundance in NEFs, but a consistent decrease in abundance in MEFs) were enriched in “base/nucleotide excision repair” and “DNA replication,” consistent with the notion that NEFs have a strong base/nucleotide excision repair function in response to stress stimuli. Genes in cluster II (a group of genes showing a consistent increase in abundance in NEFs but a consistent decrease in abundance in MEFs) were enriched in the “MAPK signaling pathway” and “PI3K-AKT signaling pathway,” which could be associated with the hypoxic response through regulation of hypoxia-induced factor-1α (HIF-1α) expression [24,25]. A previous study suggested that the MAPK signaling pathway contributes to hypoxic adaptation in NMRs, and inhibition of MAPK8IP1 and MAPK10 expression can induce apoptosis and arrest cell growth in NMR fibroblasts following anoxia [26]. Genes in cluster III (a group of genes showing a nonsignificant increase in abundance under anoxia in NEFs but a significant increase in abundance at the early or later time points during anoxia in MEFs) were enriched in the “HIF-1 signaling pathway,” “MAPK signaling pathway,” and “mTOR signaling pathway,” indicating that hypoxia-related pathways are active in NEFs and MEFs (**Figs 1E, 1F, and S4**). Genes in cluster IV (a group of genes showing a consistent decrease in abundance in NEFs but little or no increase in abundance in MEFs) were enriched in “Ribosome” and “rRNA processing,” which is interesting as hierarchical clustering analyses of proteomic data did not recover other abundance profiles but revealed an enrichment of genes related to Ribosome (**Figs 1E, 1F, and S4**). In addition, all 4 clusters of proteomic data contained genes with expression changes in NEFs but not in MEFs. For example, genes in clusters I and II (i.e., genes showing an increase in abundance under anoxia in NEFs but a decrease or no increase in abundance under anoxia in MEFs) were enriched in “ATPase activity” and “RNA splicing.” Genes in clusters III and IV (i.e., genes showing an increase in abundance under anoxia in MEFs but a decrease or no increase in abundance under anoxia in NEFs) were also enriched in “RNA splicing” (**S5 Fig**). These results suggest that RNA splicing was involved in cellular adaptation to anoxic conditions more in NEFs than in MEFs, consistent with previous research showing that pre-mRNA splicing contributes to cellular adaptation to hypoxic conditions [27].

An overlap in the DEGs identified among the RNA-seq and proteomics data was also found. Based on the differential expression analysis, an overlap was found to be concentrated mainly at 3 to 4 h after anoxic treatment, i.e., 500 and 369 genes overlapped in NEFs at the 3- and 4-h time points, respectively. In parallel, fewer genes overlapped in MEFs, i.e., 62 and 71 genes overlapped at the 3- and 4-h time points, respectively (**S2** and **S3 Tables**).

### Comparative time-resolved proteomics reveals that H1.2 differs between NEFs and MEFs

**Fig 2:**
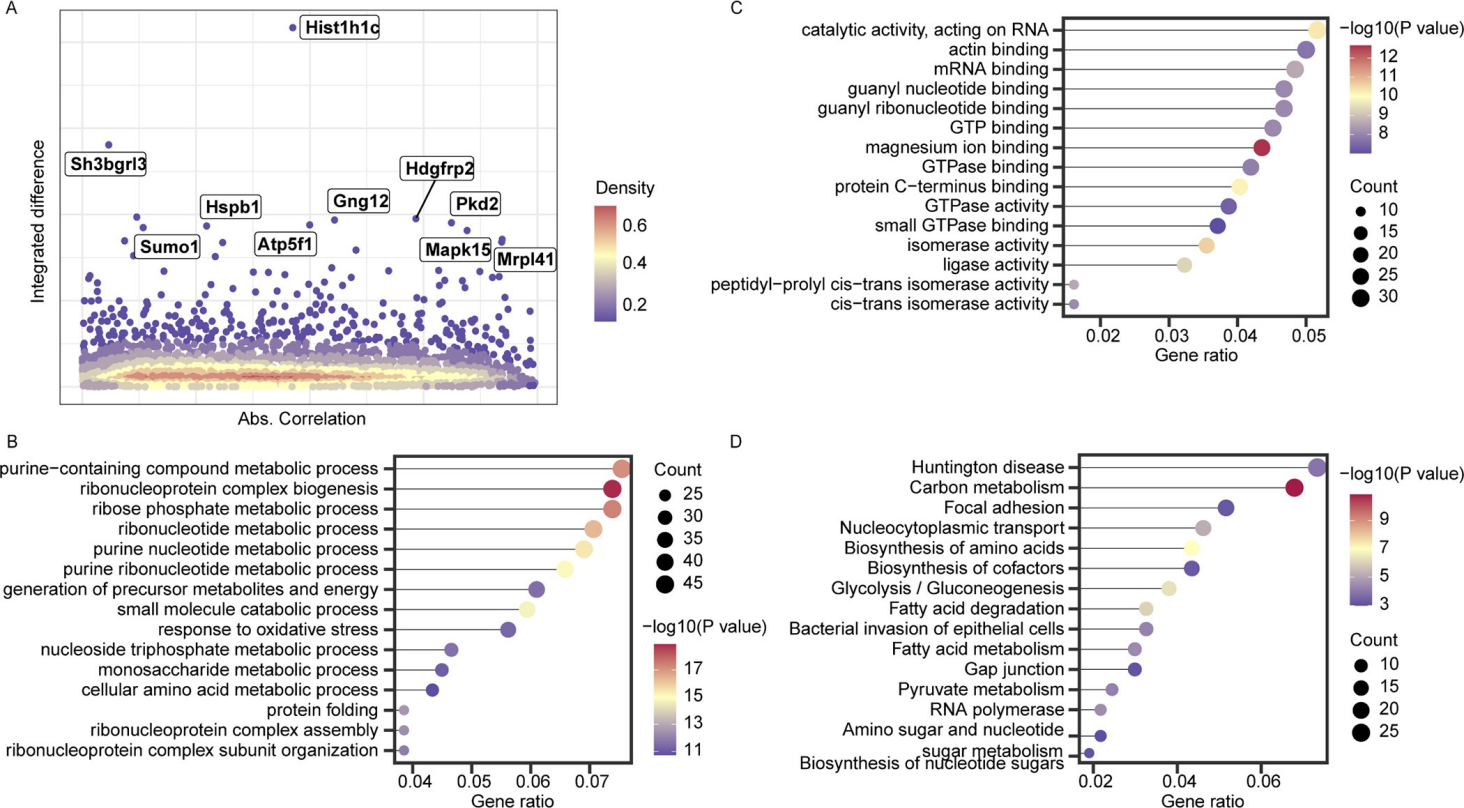
Comparative proteomics reveals that the H1.2 accumulation differs between NEFs and MEFs. **(A)** Plot of scores for genes identified in proteomics between NEFs and MEFs. The scores were determined as follows: *A* [1 –(absolute correlation coefficient)] versus *B* (integrated difference between the curves). The coordinates of proteins known to be associated with anoxia are marked with black protein names. Colors indicate the density of data points. Dot plots of enriched **(B)** BP GO terms, **(C)** MF GO terms, and **(D)** KEGG pathways among proteins with a *B* score above the 75th percentile. In **B**–**D**, the *x*-axis shows the fold enrichment of each GO term or go pathway, color represents the *p* value, and the size of dots represents the count. The data underlying the graphs shown in the figure can be found in S1 Data. BP, biological process; MEF, mouse embryonic fibroblast; MF, molecular function; NEF, NMR embryonic fibroblast.

Proteins showing sequentially different changes to anoxic conditions in NEFs and MEFs were identified using a newly developed method that calculates the change in protein abundance over time between study groups [28]. Scores for each gene were calculated using the following equation: score = (1 − |*A*|) × *B*, where *A* is the Pearson correlation between the temporal response curves for a given protein over time in NEFs and MEFs, and *B* is the integrated numerical difference between the same curves. Thus, for a certain protein, a high score indicates a large difference in abundance for a given protein between the 2 groups. Protein levels at each time point were normalized by the levels at start time point under normoxic conditions before treatment to diminish the impact due to a potentially large difference in abundance between 2 specific proteins in the 2 populations. In total, 667 proteins with significant score changes, including some genes with known functions in hypoxia and the HIF-1 signaling pathway (KEGG, *P* = 0.660 × 10^−3^), such as *Map2k1*, *Eno2*, *Pgk1*, and *Rela* (scores >0.87, corresponding to the 75th percentile; **Figs 2A and S6** and **S4 Table**), were identified. These genes were also significantly enriched in KEGG pathways “carbon metabolism” (*P* = 1.000 × 10^−4^), “glycolysis/gluconeogenesis” (*P* = 1.000 × 10^−4^), and “fructose and mannose metabolism” (*P* = 0.002; **S5 Table**). In particular, the enrichment of “fructose and mannose metabolism pathway” (*P* = 1.000 × 10^−4^) may be associated with the switch to anaerobic metabolism fueled by fructose during anoxia [10].

**Fig 3:**
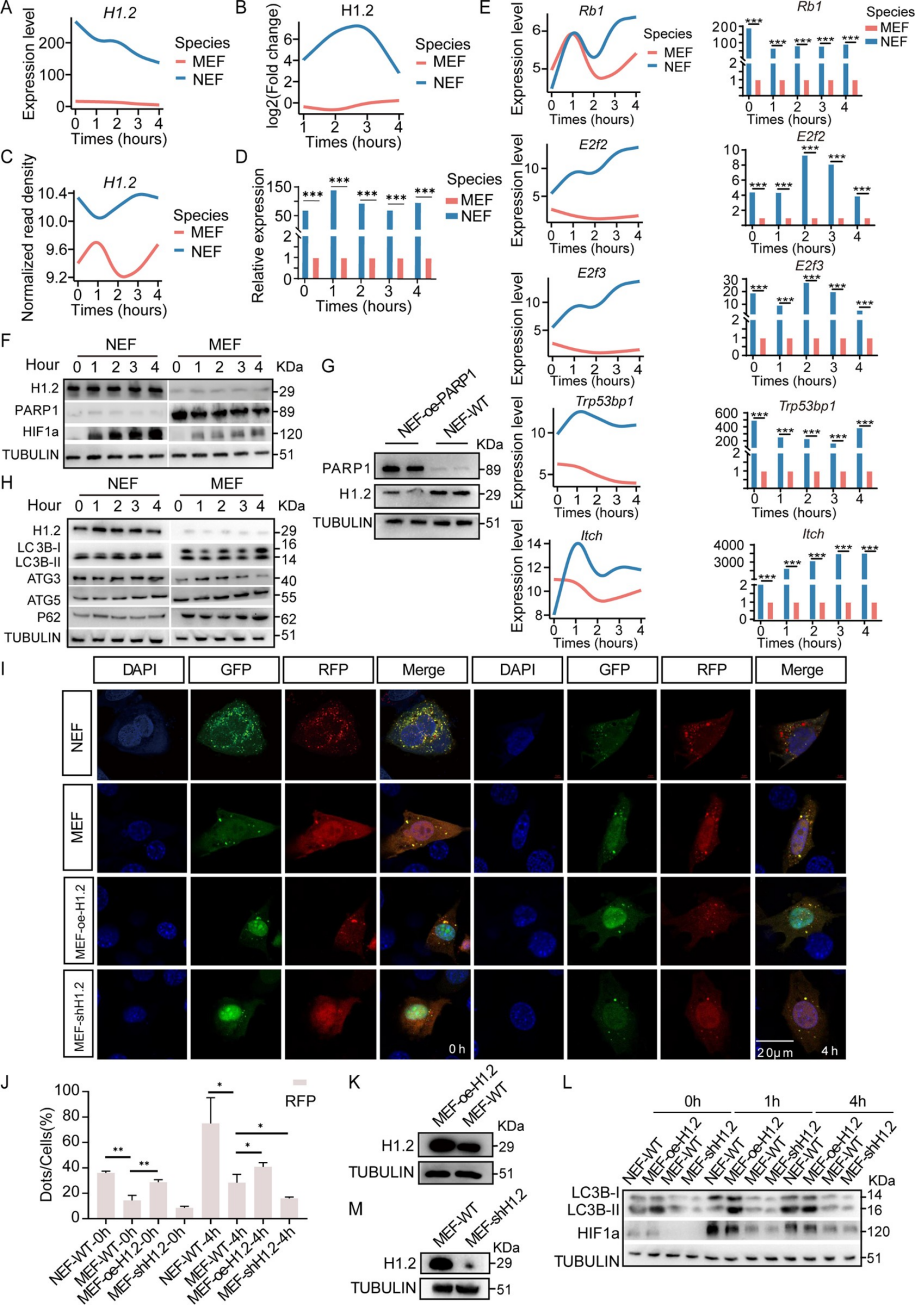
Overexpression of H1.2 induces autophagy. **(A)** RNA level of *H1*.*2* in NEFs and MEFs according to RNA-seq analysis. Times 0 represent normoxic condition, times 1, 2, 3, 4 represent at 1, 2, 3, and 4 h time points under anoxic conditions, respectively, hereinafter inclusive. **(B)** Protein level of *H1*.*2* in NEFs and MEFs according to proteomics analysis. Times 0 represent normoxic condition, times 1, 2, 3, 4 represent at 1, 2, 3, and 4 h time points under anoxic conditions, respectively, hereinafter inclusive. **(C)** Chromatin opening level of *H1*.*2* in NEFs and MEFs according to ATAC-seq analysis. Times 0 represent normoxic condition, times 1, 2, 3, 4 represent at 1, 2, 3, and 4 h time points under anoxic conditions, respectively, hereinafter inclusive. **(D)** RT-PCR results of *H1*.*2* in NEFs and MEFs. **(E)** RT-PCR results of *H1*.*2*-related genes in NEFs and MEFs. **(F)** Western blot results of *H1*.*2*, *PARP1*, and *HIF-1α* in NEFs and MEFs. **(G)** Overexpression of PARP1 in NEF inhibits the expression of *H1*.*2*. **(H)** Western blot results of different basal autophagy marker levels in NEFs and MEFs. **(I)** Representative images of the different autophagy levels at NEFs, MEFs, MEF-oe-H1.2, and MEF-shH1.2 in normoxic and anoxic conditions. **(J)** Statistical analysis of representative images of the different autophagy levels at NEFs, MEFs, MEF-oe-H1.2, and MEF-shH1.2 in normoxic and anoxic conditions. **(K)** Western blot results of the overexpression of H1.2 in MEFs. **(L)** Western blot results of the knockdown of H1.2 in MEFs. **(M)** Western blot of the different autophagy levels at NEFs, MEFs, MEF-oe-H1.2, and MEF-shH1.2 in normoxic and anoxic conditions. The data underlying the graphs shown in the figure can be found in S1 Data. MEF, mouse embryonic fibroblast; NEF, NMR embryonic fibroblast.

The top-ranked gene according to all scores was *Sh3bgrl3* (encoding SH3 domain binding glutamic acid-rich protein like 3), which is located on chromosome 1p34.3–35 and belongs to the thioredoxin superfamily [29]. *Sh3bgrl3* is associated with TNF-α-induced apoptosis inhibition and cell survival promotion [29]. High *Sh3bgrl3* expression is found in glioblastoma and associated with worse survival in affected patients [29]. The *Sh3bgrl3* RNA expression was found to be higher in MEFs than that in NEFs throughout the 4-h anoxia treatment period (**S7 Fig**); however, the protein level and chromatin accessibility of *Sh3bgrl3* in MEFs were not consistent with the *Sh3bgrl3* RNA level (**S7 Fig**). The second-ranked gene was *HIST1H1C*, which encodes histone H1.2 from the histone H1 family. H1.2 interacts with linker DNA between nucleosomes and plays a role in chromatin compaction into higher-order structures [30]. Importantly, both the RNA and protein levels of *H1*.*2* were increased in NEFs compared with those in MEFs (**Fig 3A and 3B**).

Consistently, ATAC-seq revealed that the *H1*.*2* gene was more accessible in NEFs than in MEFs (**Fig 3C**). We also obtained the expression levels of *H1*.*2* in tissues of different rodents, founding that *H1*.*2* were generally highly expressed in the liver, kidney, and brain of NMRs compared with other rodents (**S8 Fig**). Previous studies suggested that *H1*.*2* plays important roles in multiple cellular processes, including apoptosis [31], gene transcription, and protein folding [32–34]. In addition, *H1*.*2* phosphorylation has been found to regulate *p53* acetylation and play a role in DNA damage repair [35]. However, why *H1*.*2* is highly expressed in NMRs or how the gene functions in the cellular response to anoxic stress are unclear.

### H1.2 overexpression induces autophagy, HIF-1α, and glycolytic proteins

Reverse transcription-polymerase chain reaction (RT-PCR) and western blotting confirmed the higher H1.2 expression in NEFs relative to that in MEFs at both mRNA and protein levels (**Fig 3D and 3F**). RT-PCR was also used to explore the expression levels of H1.2-related genes, finding that *Rb1*, *E2f2*, *E2f3*, *Itch*, and *Trp53bp1* were expressed at higher levels in NEFs than those in MEFs (**Fig 3E**). These results support the finding that *p53* is up-regulated in NMRs compared with its expression in mice [14]. A previous study also showed that *H1*.*2* is a target gene of *PARP1*, which can inhibit H1.2 activation [36]. Consistently, the expression level of *PARP1* in MEFs was found to be higher than that in NEFs (**Fig 3F**). PARP1 overexpression in NEFs significantly decreased the expression level of H1.2 compared with that in wild-type (WT) NEFs (**Fig 3G**). Another prior study revealed that H1.2 is related to autophagy and that NMRs show higher levels of autophagy [37]. Autophagy-related markers were assessed and LC3B-I to LC3B-II conversion and ATG3 were found to be significantly increased in NEFs compared with MEFs (**Fig 3H**). Additionally, transfection of mRFP-GFP-LC3 significantly increased the percentage of autophagic flux in NEFs compared to MEFs in normoxic condition (*P* = 0.001) and anoxic conditions (**Fig 3I and 3J**; *P* = 0.02).

To further explore the relationship between H1.2 and autophagy, PEGFP-N1-Mus-H1.2 was transfected into MEFs (**Fig 3K**), in which H1.2 overexpression induced the conversion of LC3B-I to LC3B-II in both normoxic condition and anoxic conditions (**Fig 3L**). H1.2 and mRFP-GFP-LC3 cotransfection significantly increased the percentage of autophagic flux compared with that in WT MEFs in normoxic condition (*P* = 0.006) and anoxic conditions (**Fig 3I and 3J**; *P* = 0.041). Furthermore, both LC3B-I to LC3B-II conversion and the percentage of autophagic flux decreased in shH1.2 MEF cells (**Fig 3I, 3J, 3L, and 3M**; anoxic conditions, *P* = 0.032). These results suggest that H1.2 overexpression induces autophagy and that knockdown of H1.2 inhibits autophagy vice versa.

**Fig 4:**
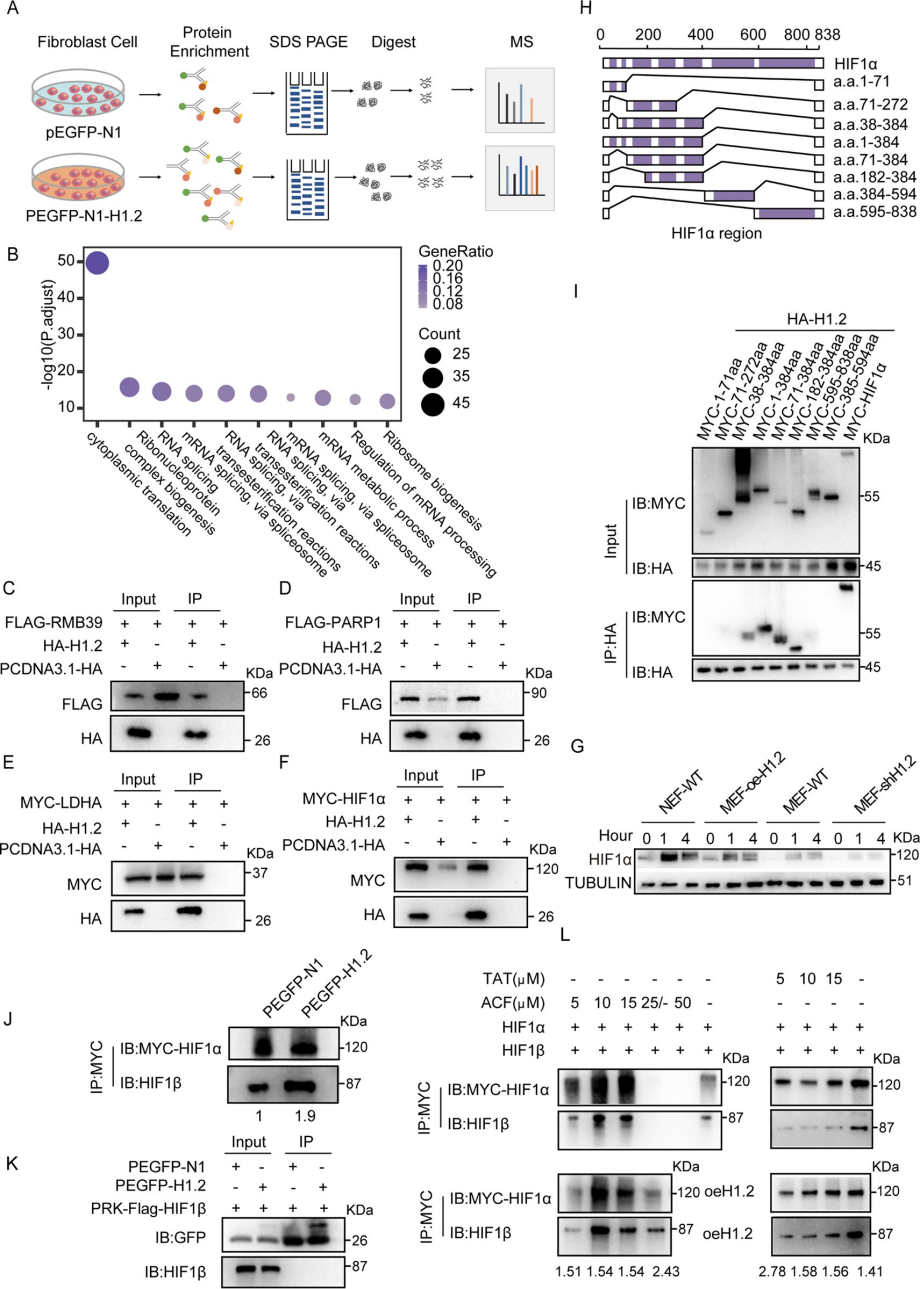
H1.2 overexpression induces high *HIF-1α* expression. **(A)** Schematic representation of Co-IP followed by LC-MS/MS. **(B)** BP GO term enrichment analysis of H1.2 interaction proteins identified via Co-IP/MS. **(C–F)** Co-IP analysis shows that exogenous H1.2 interacts with LDHA, RBM39, PARP1, and HIF-1α proteins in HEK293T cells. **(G)** Histone H1.2 overexpression in MEFs induced up-regulation of the HIF-1α protein level, whereas knockdown of histone H1.2 in MEFs significantly decreased HIF-1α protein levels. Times 0 represent normoxic condition, times 1, 4 represent at 1 and 4 h time points under anoxic conditions, respectively, hereinafter inclusive. **(H, I)** Co-IP using purified fragments of MYC-HIF-1α and HA-H1.2 revealed that the HIF-1α PAS domain interacts directly with HIST1H1C. **(J)** Co-IP analysis shows that exogenous H1.2 could enhance HIF-1α: HIF-1β interaction in HEK293T cells. **(K)** Co-IP analysis shows that exogenous H1.2 could not interact with HIF-1β protein in HEK293T cells. **(L)** Co-IP analysis shows that exogenous H1.2 could reduce the inhibition of HIF-1α: HIF-1β interaction by ACF and TAT. The data underlying the graphs shown in the figure can be found in S1 Data. BP, biological process; MEF, mouse embryonic fibroblast; MS, mass spectrometry.

We then pursued the identification of interaction partners of H1.2 by expressing GFP-tagged H1.2 in HEK293T cells and performing coimmunoprecipitation (Co-IP) and MS testing (**Fig 4A**). In total, 451 proteins were identified, which were enriched in “RNA Splicing and mRNA metabolic processing” (**Fig 4B**). The MS results confirmed several proteins that had previously interacted with H1.2, including RBM39 and PARP1. An interaction between H1.2 and LDHA, which may be associated with hypoxia [38], was also confirmed by our MS analyses (**Fig 4C–4E**). Importantly, Co-IP assay identified HIF-1α as an interacting partner of H1.2 (**Fig 4F**). Indeed, H1.2 overexpression in MEFs induced *HIF-1α* up-regulation, whereas knockdown of histone *H1*.*2* reduced *HIF-1α* expression (**Fig 4G**). Co-IP with purified fragments of MYC-HIF-1α and HA-H1.2 was also used to determine which fragment of HIF-1α directly interacts with H1.2. A fragment containing a specific region of the Per-ARNT-Sim (PAS) domain, which is recognized as a module for protein–protein interaction and stabilizing the HIF heterodimer, was also found to interact directly with H1.2 (**Fig 4H and 4I**).

It is well known that HIF-1 is a heterodimeric protein that is composed of HIF-1α and HIF-1β subunits, which belong to the family of basic helix–loop–helix (bHLH) transcription factors [39]. In hypoxic condition, HIF-1α transfer to nucleus and binds to HIF-1β to form a heterodimer, further activating downstream signaling pathways in response to hypoxia [39]. Co-IP with purified fragments of MYC-HIF-1α, FLAG-HIF-1β, and GFP-H1.2 shows that exogenous H1.2 could enhance HIF-1α: HIF-1β interaction in HEK293T cells (**Fig 4J**). Co-IP assay further shows that exogenous H1.2 could not interact with HIF-1β protein in HEK293T cells (**Fig 4K**). We then use Acriflavine (ACF) and TAT(TAT-cyclo-CLLFVY) to inhibit dimerization of HIF-1α and HIF-1β, whereas Co-IP analysis shows that exogenous H1.2 could reduce the inhibition of HIF-1α: HIF-1β interaction by ACF and TAT (**Fig 4L**). These results suggest that H1.2 overexpression could enhance the dimerization of HIF-1α with HIF-1β.

To further assess the impact of H1.2 overexpression, we conducted RNA-seq analysis on MEFs engineered to overexpress H1.2. Our findings reveal that H1.2 overexpression significantly boosts the expression of genes predominantly involved in the glycolytic and carbon metabolism pathways (**S9 and S10 Figs)**. Furthermore, a focused protein expression analysis of key glycolytic enzymes confirmed an up-regulation of *PKM*, *LDHA*, *GLUT1*, *HK2*, and *HK1* in H1.2-overexpressing MEFs relative to the control group (**S11 Fig**). Collectively, these results indicate that H1.2 overexpression potentially augments the expression of genes associated with autophagy, hypoxia sensing, and glycolytic pathways.

### H1.2 inhibits migration and invasion of cancer cells

**Fig 5:**
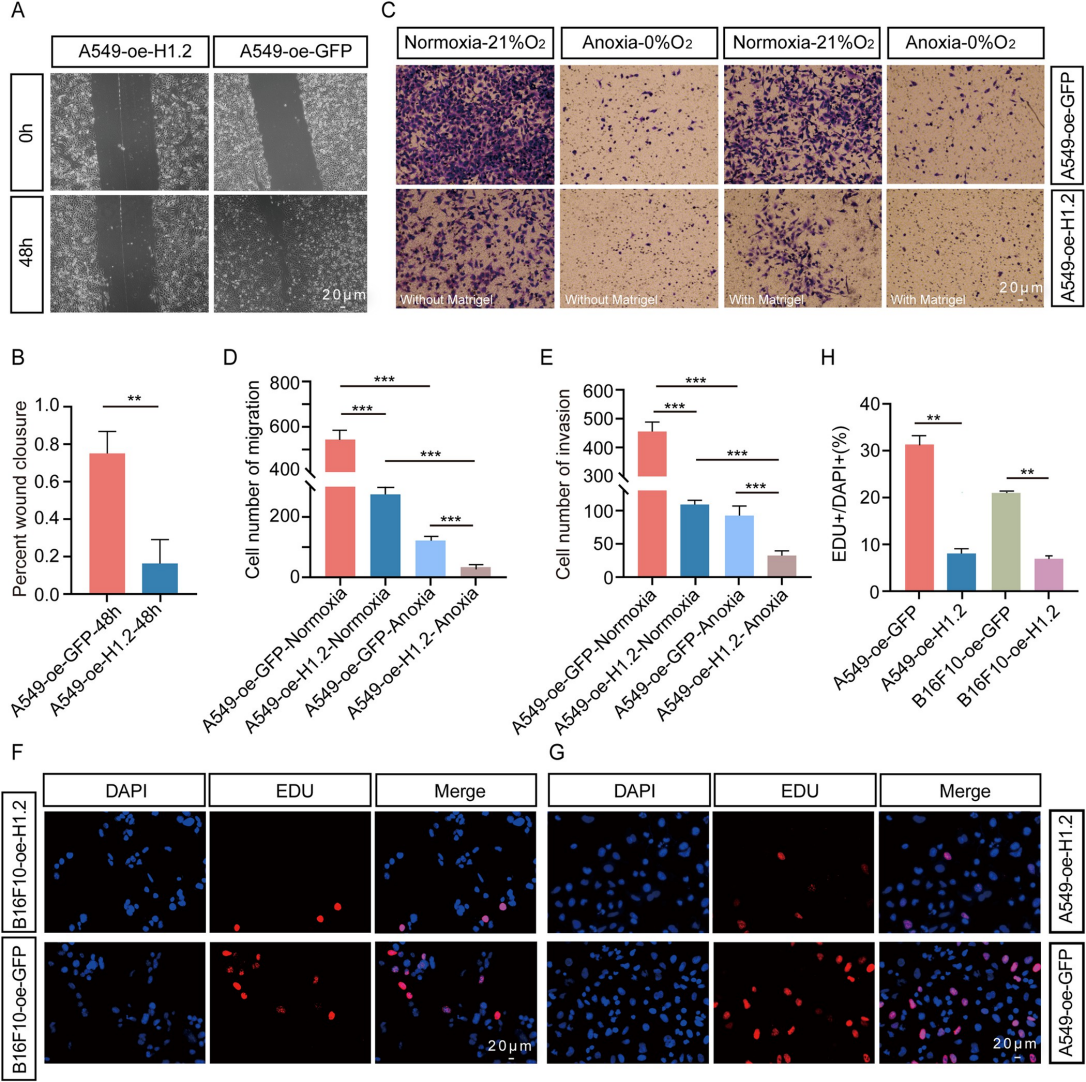
H1.2 inhibits migration and invasion of A549 cells. **(A, B)** Effect of H1.2 on A549 cell migration according to a wound-healing assay. **(C–E)** Effect of H1.2 on A549 cell migration under normoxic and anoxic conditions according to a Transwell assay. The number of migrated cells was normalized to that of the control group. *P < 0.050, **P < 0.010, ***P < 0.001. **(F, G)** H1.2 overexpression in A549 and B16F10 cells markedly decreased the ratio of EdU-positive cells. **(H)** Quantification data for the EDU assays in B16F10 and A549 cell lines. *P < 0.050, **P < 0.010, ***P < 0.001. The data underlying the graphs shown in the figure can be found in S1 Data.

**Fig 6:**
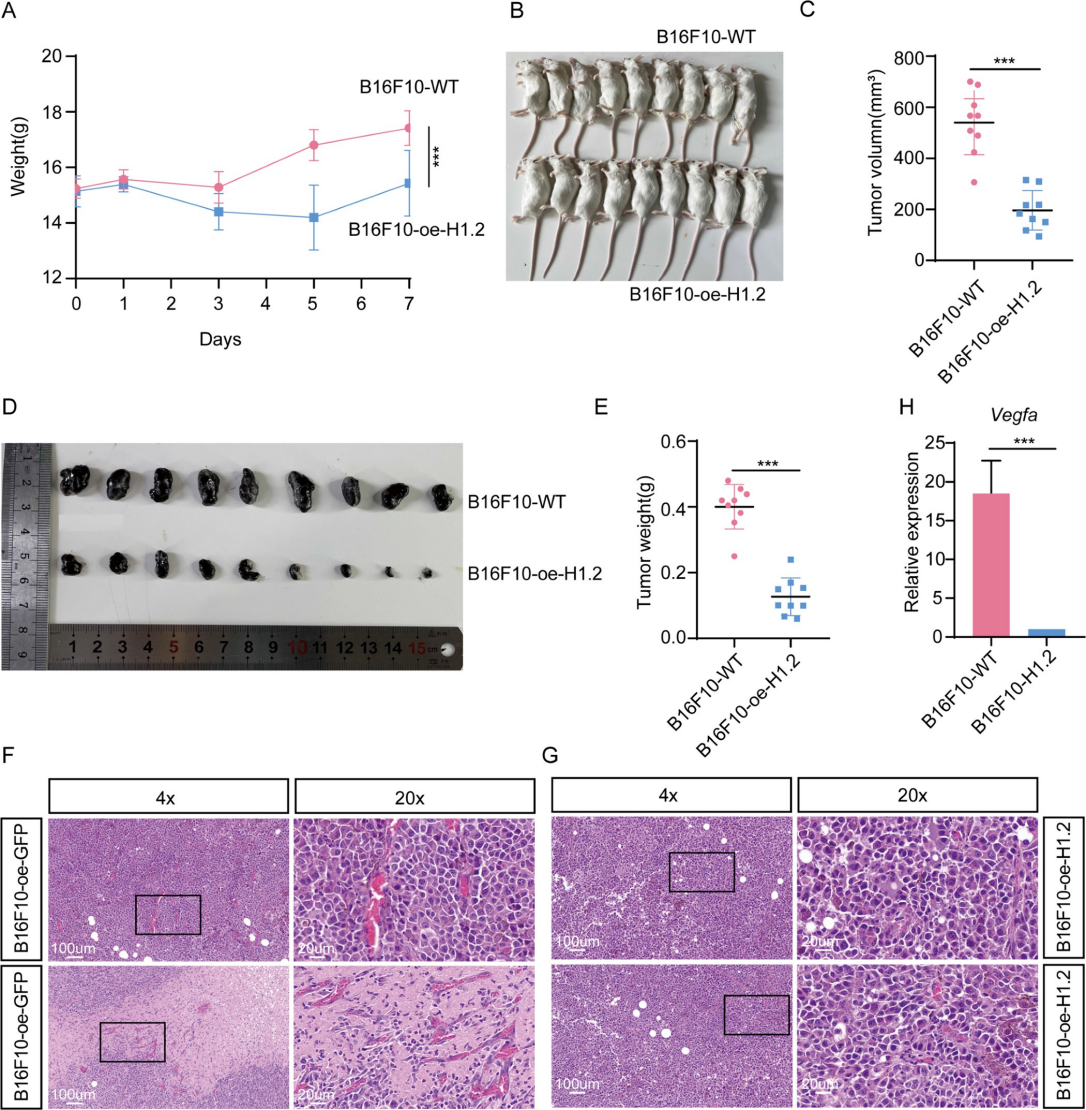
H1.2 overexpression inhibits tumor proliferation and formation. **(A)** Mice were weighed the day after the corresponding treatment. Days 0, 1, 2, 3, 4, 5, 6, and 7 represent the day after the corresponding treatment, respectively. **(B, C)** Mice were killed on day 7 using cervical dislocation, and tumor volume in the H1.2 overexpression and WT conditions were measured. **(D)** Tumors in the H1.2 overexpression and WT conditions were removed for observation. **(E)** Tumor weight in the H1.2 overexpression and WT conditions was measured on day 7. **(F)** HE staining revealed significant angiogenesis in the center of tumor tissues in mice treated with WT B16F10 cells. **(G)** No obvious angiogenesis was observed in the center of tumor tissues in mice treated with B16F10 cells overexpressing H1.2. **(H)** RT-PCR analysis of expression of the angiogenesis-related gene *VEGFA* in H1.2-overexpressing B16F10 cells and WT B16F10 cells. *P < 0.050, **P < 0.010, ***P < 0.001. The data underlying the graphs shown in the figure can be found in S1 Data. RT-PCR, reverse transcription-polymerase chain reaction; WT, wild-type.

We further examined the potential H1.2 function in cancer because H1.2 was found to induce autophagy, and autophagy was reported to have a context-dependent role in cancer [37,40]. To assess the *H1*.*2* effects on the migratory and invasive properties of cancer, ectopic expression of mouse GFP-tagged H1.2 in human lung adenocarcinoma cell line (A549) was forced. Wound healing assays indicated that the migration rate of A549 cells with H1.2 expression was 16.30% ± 12.76%, much lower than the 75.02% ± 11.77% of A549 cells with the control vector (**Fig 5A and 5B**). Transwell migration assays revealed that A549/H1.2 cells had a lower migration ability (274.70 ± 40.43 without Matrigel and 92.33 ± 14.29 with Matrigel) compared with that of A549/vector cells (543.70 ± 33.72 without Matrigel and 455.70 ± 32.50 with Matrigel; **Fig 5C–5E**). The development of cancer cells involves an anoxic microenvironment, and the results of the current study suggest that H1.2 contributes to the response of NMRs to anoxia. The transwell migration assays were then repeated under anoxic conditions, finding that A549/H1.2 cells had a lower migration ability (34.00 ± 3.46 without Matrigel and 32.33 ± 7.09 with Matrigel) compared with that of A549/vector cells (121.70 ± 6.43 without Matrigel and 109.00 ± 6.25 with Matrigel; **Fig 5C–5E**). DNA synthesis using Click-iT EdU Alexa Fluor Imaging was also examined, discovering that *H1*.*2* overexpression markedly decreased the ratio of EdU-positive cells in A549 cells, suggesting that *H1*.*2* overexpression potentially inhibits cell proliferation (**Fig 5F–5H**). Finally, a xenograft tumor formation was used to examine a H1.2 function in vivo. Specifically, 5-week-old female Balb/c mice were randomly divided into treatment groups and injected subcutaneously with WT mouse melanoma cell line (B16F10) or B16F10 cell lines that stably overexpressed *H1*.*2* (3 × 10^6^ cells/point). Treated mice and tumor growth were monitored and measured daily. Subcutaneous tumors were observed in the WT control group, whereas *H1*.*2* overexpression markedly retarded tumor formation, weight, and volume relative to that in WT B16F10 cells (**Fig 6A–6E**). After mice were killed, tumor samples were removed and stained with HE to quantify microscopic changes. Significant angiogenesis was observed in the center of tumor tissues in mice treated with WT B16F10 cells (**Fig 6F**), whereas no obvious angiogenesis was observed in mice treated with B16F10 cells that overexpressed *H1*.*2* (**Fig 6G**). RT-PCR was also used to measure the expression levels of the angiogenesis-related gene *VEGFA* in H1.2-overexpressing B16F10 and WT B16F10 cells, and the RNA level of *VEGFA* in WT B16F10 cells was increased relative to that in H1.2-expressing B16F10 cells (**Fig 6H**). In summary, H1.2 overexpression seemingly inhibits the proliferation and migration of cancer cells as well as xenograft tumor formation.

### H1.2 induced autophagic via NRF2/P62 pathway in A549 and B16F10 cells

**Fig 7:**
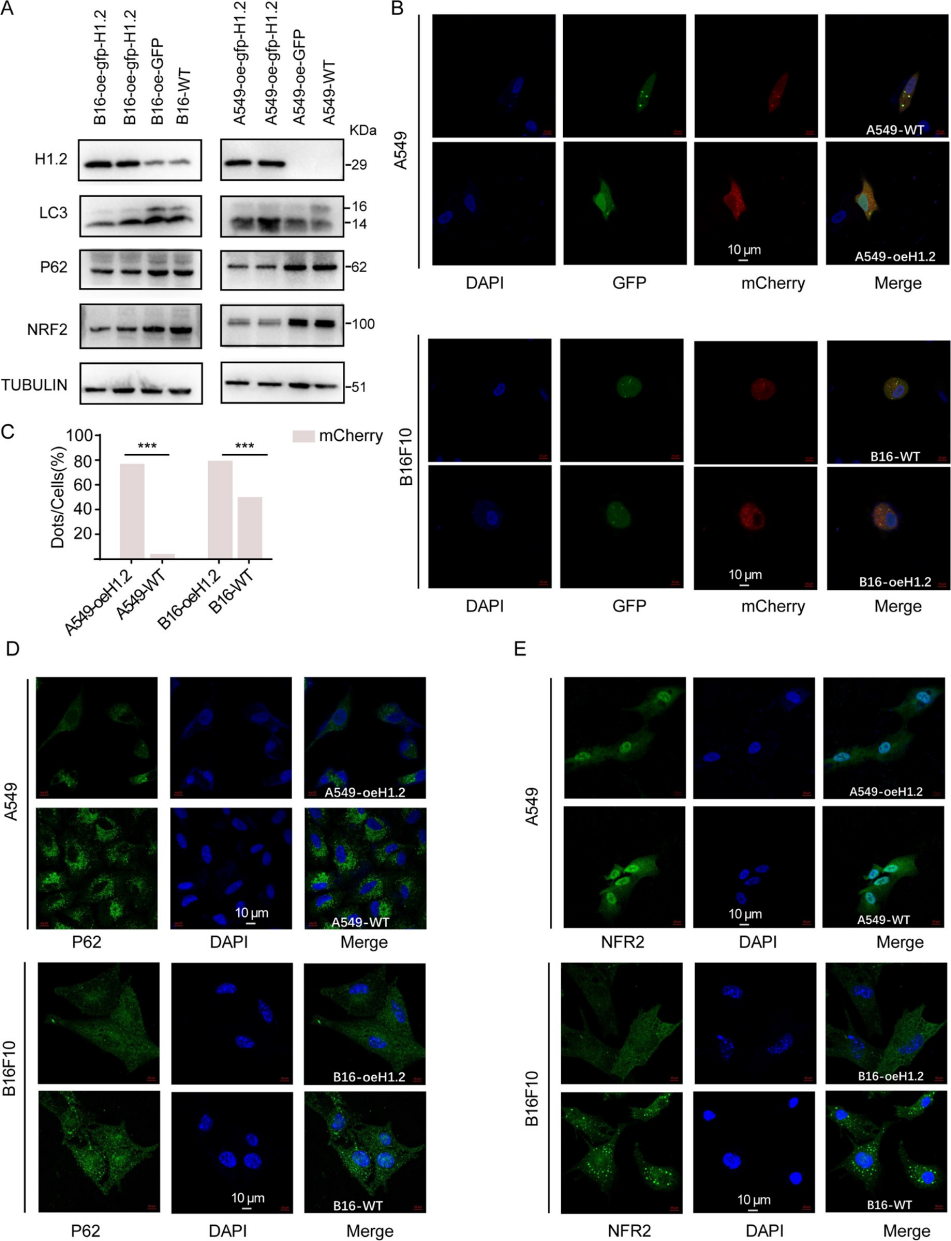
H1.2 induced autophagic via Nrf2/p62 pathway in A549 and B16F10 cells. **(A)** Western blot analysis of LC3, p62, and Nrf2 expressions in WT-A549, WT-B16F10, overexpression of H1.2 in A549 and B16F10 cells. **(B, D, E)** Immunofluorescence staining of LC3(B), p62 (C), and Nrf2 (D) in WT-A549, WT- B16F10, overexpression of H1.2 in A549 and B16F10 cells. (LC3: green and red; Nrf2 and p62: green; DAPI: blue) Scar bar = 10 μm. **(C)** The percentage of autophagic flux in WT-A549, WT-B16F10, overexpression of H1.2 in A549 and B16F10 cells. The data underlying the graphs shown in the figure can be found in S1 Data. WT, wild-type.

Previous studies have shown that suppression of autophagy could lead to p62 accumulation, which in turn promote tumor development through increased levels of ER-stress and DNA-damage-stress [41,42]. Consistently, loss of p62 in ATG5 or ATG7-depleted mouse models were found to suppress tumor growth, suggesting there is a correlation between p62 accumulation and adenoma formation [43,44]. At the same time, inhibition of nuclear factor (erythroid-derived 2)-like 2 (Nrf2) expression and activity increases the anticancer activity of erastin and sorafenib in HCC cells [45]. Moreover, apatinib treatment could induce autophagy and apoptosis by promoting ROS generation and inhibiting NRF2 and P62 expression [46]. We next sought to explore whether overexpression of H1.2 in A549 and B16F10 cells cause autophagy. Our results showed that the expression level of LC3-II form was markedly up-regulated in both A549/H1.2 and B16F10/H1.2 compared with that of A549/WT and B16F10/WT cells (**Fig 7A–7C**). Additionally, a decrease of p62 expression was significantly detected in both A549/H1.2 and B16F10/H1.2 compared with that of A549/WT and B16F10/WT cells (**Fig 7A and 7D**). We also observed that the protein level of NRF2 was eventually decreased in both A549/H1.2 and B16F10/H1.2 compared with that of A549/WT and B16F10/WT cells (**Fig 7A and 7E**). Collectively, these results suggested that overexpression of H1.2 induced autophagic and tumor inhibition through NRF2/P62 pathway.

### H1.2 transgenic mice show prolonged hypoxic survival time and retarded tumor formation

**Fig 8:**
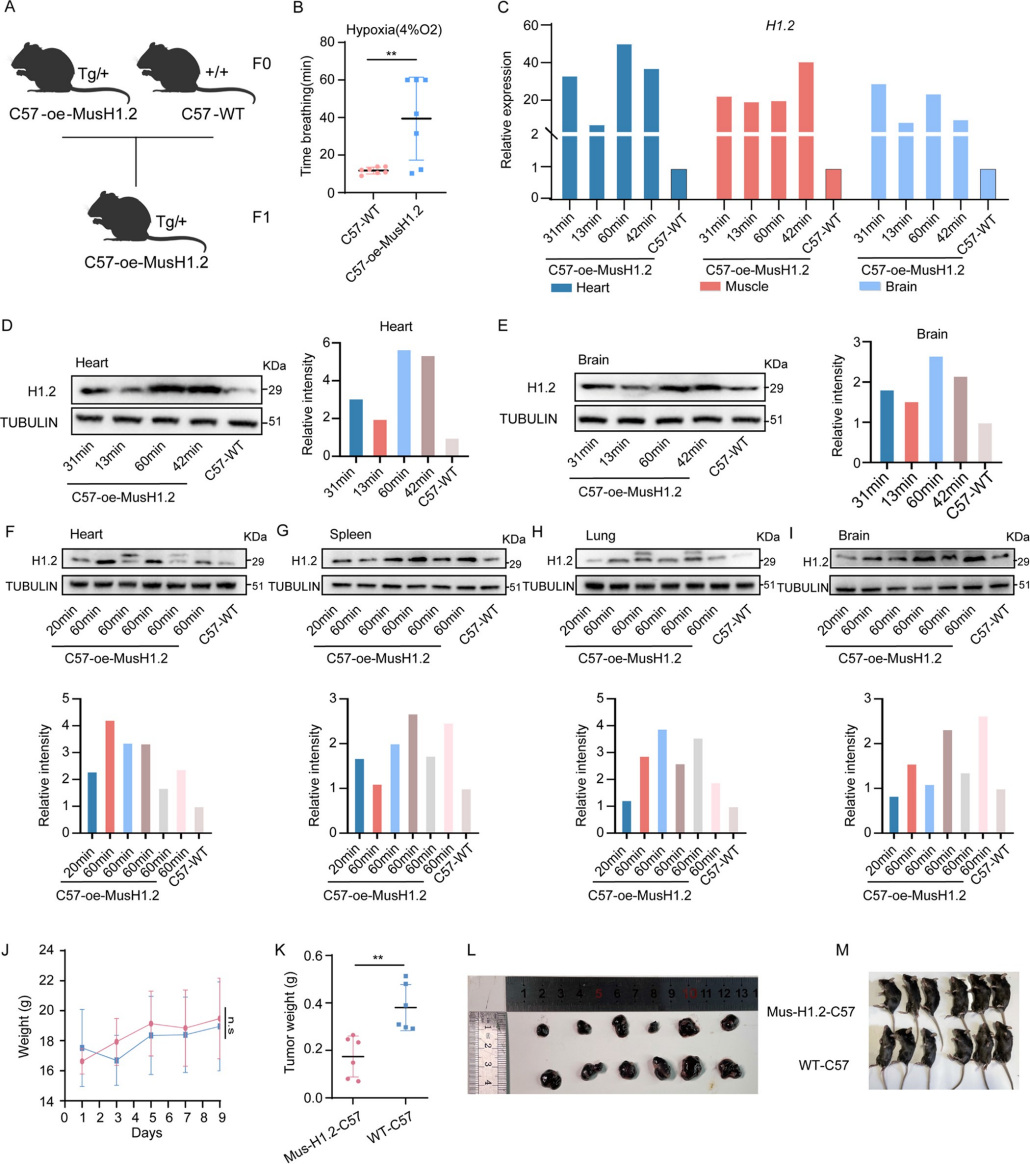
H1.2 transgenic mice show prolonged hypoxic survival time and retarded tumor formation. **(A)** Knock-in H1.2 transgenic mice (C57-oe-MusH1.2) were generated in C57BL background using a microinjection approach. This panel is created with BioRender.com. **(B)** Breathing time of WT-C57 and C57-oe-MusH1.2 female mice under 4% O_2_ conditions. **(C)** Relative expression level of *H1*.*2* according to different breathing times in C57-oe-MusH1.2 and WT-C57 female mice. **(D, E)** Western blot results (left panel) with the respective quantitative densitometric results (right panel) of H1.2 levels according to different breathing times in C57-oe-MusH1.2 and WT-C57 female mice. **(F–I)** Western blot results (upper panel) with the respective quantitative densitometric results (lower panel) of H1.2 levels according to different breathing times in brain of C57-oe-MusH1.2 and WT-C57 male mice. **(J, K)** Xenograft of cancer line into H1.2 transgenic mice. **(J)** Mice were weighed the day after the corresponding treatment. Days 0, 1, 2, 3, 4, 5, 6, 7, 8, and 9 represent the day after the corresponding treatment, respectively. **(K)** Mice were killed on day 9 using cervical dislocation, and tumor volume in C57-oe-MusH1.2 and WT-C57s mice were measured. **(L)** Tumors in in C57-oe-MusH1.2 and WT-C57s mice were removed for observation. **(M)** Mice were killed on day 9 via cervical dislocation. *P < 0.050, **P < 0.010, ***P < 0.001. The data underlying the graphs shown in the figure can be found in S1 Data. WT, wild-type.

Knock-in H1.2 transgenic mice (C57-oe-MusH1.2) were generated in C57BL background using a microinjection approach reported previously to explore the H1.2 functional consequences at the organismal level [47,48] **(Fig 8A)**. The expression of H1.2 in C57-oe-MusH1.2 and C57-WT was detected, and the high expression level of H1.2 in C57-oe-MusH1.2 compared with that in C57-WT was validated (**S12 Fig**). The daily energy expenditure the C57-oe-MusH1.2 was slightly lower than that of the C57-WT (**S13 Fig**). Then, 6-week-old WT C57 and C57-oe-MusH1.2 mice were subjected to controlled hypoxia at 4% O_2_ using atmospheric chambers (all procedures were approved by local ethics committees). C57-oe-MusH1.2 mice were able to tolerate this chronic hypoxic environment for 39 min and 53 min on average, for female and male groups, respectively, whereas WT C57 mice died at <12 min on average (**Figs 8B and S12**). The relative H1.2 expression levels in the heart and brain of female C57-oe-MusH1.2 mice were identified to determine whether the relative basal H1.2 expression led to different survival times, finding that they were positively correlated with survival time (heart, *r* = 0.957, *P* = 0.010; brain, *r* = 0.968, *P* = 0.006; **Fig 8C–8E**). Of the male C57-oe-MusH1.2 mice, the relative H1.2 expression levels in lung and brain were positively correlated with survival time (lung, *r* = 0.814, *P* = 0.030; brain, *r* = 0.613, *P* = 0.048; **Fig 8H and 8I**), but not in heart and spleen (**Fig 8F and 8G**).

**Fig 9:**
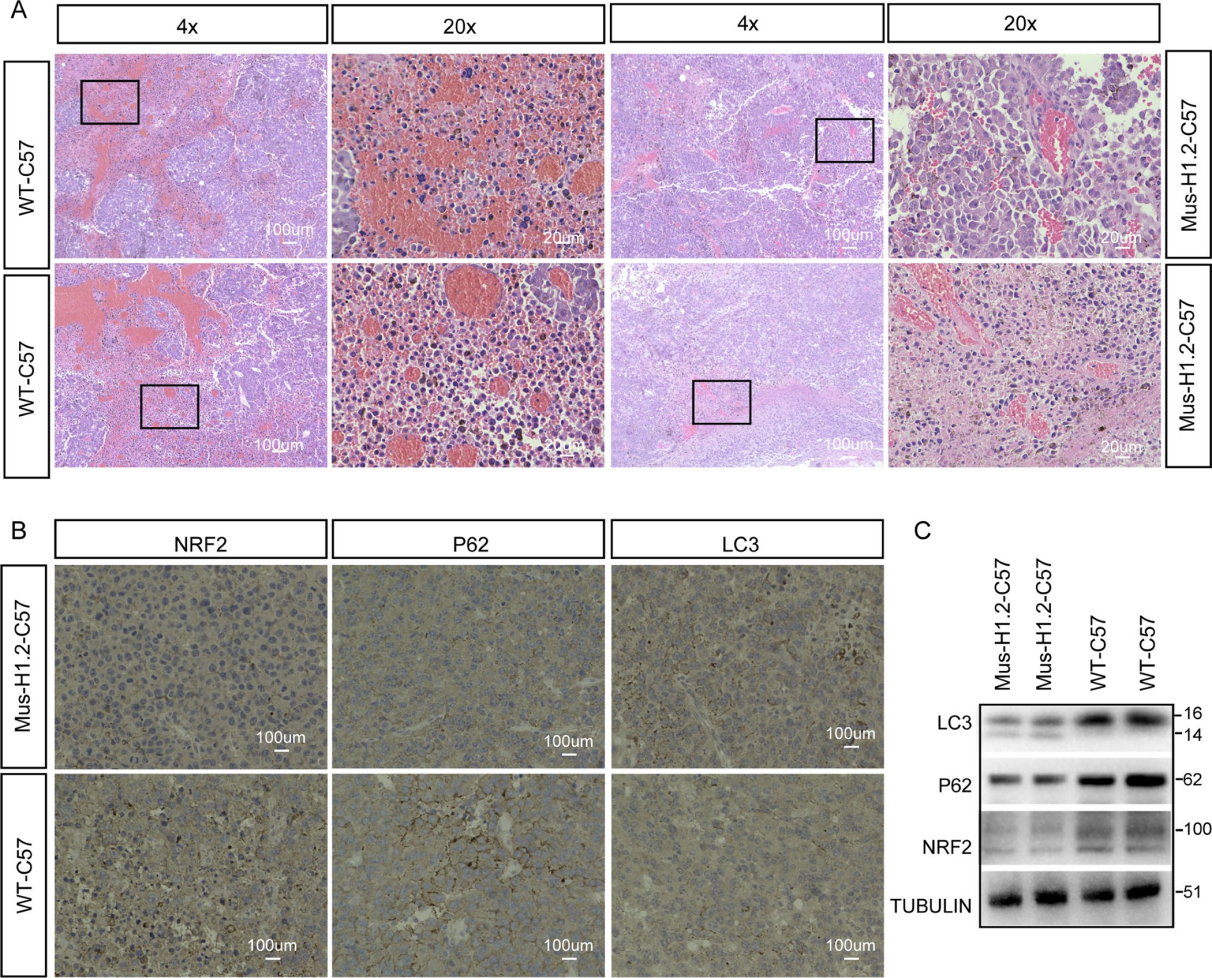
C57-oe-MusH1.2 inhibited tumor proliferation and formation through P62/NRF2 pathway. **(A)** HE staining revealed significant angiogenesis in the center of tumor tissues in WT C57. **(B)** Immunohistochemical results of LC3, p62, and Nrf2 expressions in WT C57 and C57-oe-MusH1.2. **(C)** Western blot analysis of LC3, p62, and Nrf2 expressions in WT C57 and C57-oe-MusH1.2. WT, wild-type.

We then examined xenograft tumor formation in the knock-in H1.2 transgenic mice. Five-week-old WT C57 mice and C57-oe-MusH1.2 mice were divided into 2 groups and injected subcutaneously with WT B16F10 cell lines (3 × 10^6^ cells/point), after which they were monitored daily. First, subcutaneous tumors were observed in WT C57 mice, whereas C57-oe-MusH1.2 mice showed markedly retarded tumor formation, weight, and volume relative to that in the control mice (**Fig 8J–8M**). Second, we observed that significant angiogenesis in the tumor tissues in WT C57 mice (**Fig 9A**), whereas no obvious angiogenesis in the tumor tissues of C57-oe-MusH1.2 mice (**Fig 9A**). Third, the expression level of LC3-II form was markedly up-regulated in WT C57 mice compared with C57-oe-MusH1.2 mice (**Fig 9B**).

Additionally, a decrease of autophagy related-protein p62 expression was significantly detected in C57-oe-MusH1.2 mice compared with WT C57 mice (**Fig 9B**). We also observed that the protein level of NRF2 was eventually decreased in WT C57 mice compared with that of C57-oe-MusH1.2 mice (**Fig 9B**). Western blotting results showed that the expression level of LC3-II form was markedly up-regulated in WT C57 mice compared with C57-oe-MusH1.2 mice, while the expression level of P62 and NRF2 expression were significantly down-regulated in WT C57 mice compared with C57-oe-MusH1.2 mice (**Fig 9C**). Collectively, these results evidenced that C57-oe-MusH1.2 mice shown autophagic and tumor inhibition through NRF2/P62 pathway.

## Discussion

The extraordinary hypoxic adaptation and cancer resistance observed in naked mole rats have piqued the scientific community’s interest; however, the question of whether these traits are interconnected and the identification of the molecular mechanisms that could link these 2 phenotypes remains largely unresolved. In this case, this study first provides a comparative time-series multi-omics analysis characterizing the cellular response of NEFs and MEFs to anoxia. Protein and RNA abundance levels were found only to be moderately correlated at different time points under anoxia (Pearson correlation: NEF mRNA versus protein, *r* = 0.455–0.500; MEF mRNA versus protein, *r* = 0.602–0.618; **S14 Fig**), indicating that protein abundances in NEFs and MEFs are determined by not only transcript levels and transcriptional activity but also posttranscriptional mechanisms. An inverse correlation was even observed between mRNA and protein levels of several genes in response to anoxia. For example, an increase in the mRNA levels of *Bsg* and *Polr2a* was observed in response to anoxia in MEFs, whereas the abundance of the corresponding proteins decreased (**S1 Table**). Similarly, *Hmbs* mRNA was observed to decrease in response to anoxia in MEFs, whereas the abundance of the corresponding protein increased (**S1 Table**). These results confirm the importance of measuring both mRNA and protein levels directly, rather than relying on a singular approach.

In addition to uncovering the involvement of several pathways known to be critical in hypoxic response—such as the HIF-1 signaling pathway, MAPK signaling pathway, and mTOR signaling pathway—our study identified the gene *HIST1H1C*, which encodes the histone H1.2 protein, as an anoxia-associated factor in NMRs for the first time. Notably, *HIST1H1C* exhibited elevated expression in NEFs in comparison to MEFs. Furthermore, H1.2 was shown to enhance the expression of HIF-1α, an essential molecule for cellular oxygen sensing. Our data demonstrated that high levels of H1.2 expression could promote the dimerization of HIF-1α with its partner HIF-1β, sustaining increased levels of HIF-1α. This mechanism likely contributes to NMRs’ efficient anoxic response, aligning with previous research indicating that HIF-1α translocates to the nucleus upon hypoxia and binds to HIF-1β, forming an active heterodimer that then initiates downstream hypoxia-responsive signaling [39]. Moreover, this aligns with observations that HIF-1α levels are significantly higher in the brain, lung, heart, liver, kidney, and muscle of NMRs even under normoxic conditions [49,50]. Previously genetic analysis uncovered 2 amino acid substitutions located in the VHL-binding domain (T407I and V166I) of NMR HIF-1α, which could prevent the ubiquitin-dependent degradation of HIF-1α, and, thus, with adaptation to low oxygen conditions [49,51]. Other studies have also suggested that, under hypoxic conditions, miR-335, a direct regulator of HIF-1α, was reduced in NMR brains, likely contributing to the overexpression of HIF-1α in NMR brains [52]. And, the hypoxia-induced down-regulation of miR-101 (HIF-1α negative regulator) in NMR brains could inhibit the expression of VHL and promote the expression of HIF-1α [52]. Thus, further research is needed to elucidate whether and how H1.2 communicate with those factors that influencing HIF-1α stability.

Importantly, in addition to anoxia adaptation, a potential role of H1.2 in modulating anticancer effects was revealed. Wound healing and transwell migration assays showed that H1.2 overexpression inhibits migration, invasion, and in vitro proliferation of cancer cell lines. This finding is concordant with previous studies, which reported that autophagy suppresses tumors and that the monoallelic autophagy genes *ATG6* and *BECN1* are absent in 40% to 75% of human prostate, breast, and ovarian cancers [40,53,54]. In contrast, suppression of autophagy promotes cancer cell growth and Beclin-1 heterozygous disruption increases spontaneous tumorigenesis [55]. Autophagy defects can also disable chromosome instability, which may promote tumorigenesis [56]. Our study shows that H1.2 was highly expressed in NEFs, and H1.2 overexpression in A549 and B16F10 cells promote autophagy and tumor inhibition through Nrf2/p62 pathway, consistent with previous research. However, it should be noted that the H1 histone protein family consists of 11 subtypes in mammals [57]. In this study, in addition to H1.2, H1.1, H1.3, H1.4, and H1.5, were differentially expressed between NEF and MEF (*P* < 0.050; **S15 Fig**). We also found that H1.2 overexpression could alter expression of other variants in MEFs, including H1.3, H1.4, and H1.5 (*P* < 0.050; **S16 Fig**). Though our experiment cannot rule out the contribution of other H1 variants, the role of H1.2 in tumor resistance is convincing. For example, double knockdown of H1.2 and H1.4 has been found to accelerate lymphomas in mouse and mutations in H1 drive malignant transformation of primarily through 3D genome reorganization [58]. Another analysis of the growth properties of the H1.2-/- U2OS and H1.2-/- MCF7 cells revealed that the H1.2-/- cells grow faster than WT U2OS and MCF7 cells [59]. The down-regulation of H1.2 in DU145 cells can increase the number of osteoclasts formation, and ectopic expression of H1.2 in H1.2-depleted DU145 cells can rescue this phenomenon [60]. Nevertheless, given the primary models used in our study were fibroblast cultures and xenograft tumor formation assays, we cannot categorically state that it possesses universal anticancer properties across diverse cell lines, as susceptibility to H1.2-mediated suppression may vary among different cancer types.

**Fig 10:**
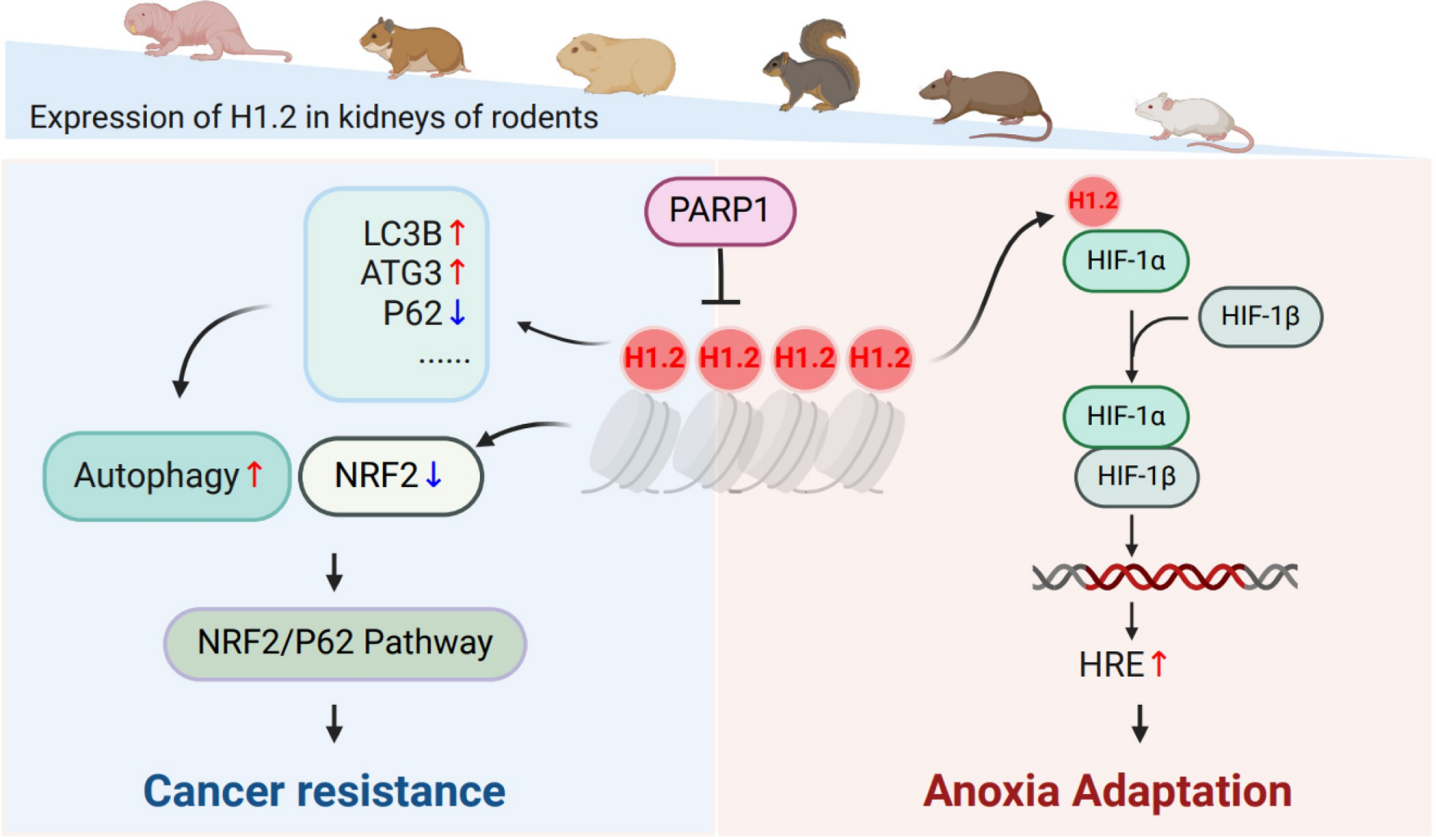
A schematic description of dual function of H1.2 in anoxia adaptation and cancer resistance. As proposed in this model, the increased H1.2 expression in NMRs was due to the inhibited *PARP1* expression. High expression of H1.2 induced an increase in autophagy under normoxic conditions as well as induced HIF-1α expression under anoxic conditions. In turn, H1.2 overexpression could enhance the dimerization of HIF-1α with HIF-1β, thereby regulating downstream signaling pathways in response to anoxia. Moreover, high H1.2 expression inhibited the migration and invasion of cancer cells as well as in vivo tumor formation, suggesting that the *H1*.*2* gene is a modulator of anoxic adaptation and cancer resistance. This figure is created with BioRender.com. NMR, naked mole rat.

In conclusion, an integrated comparative omics-based approach was used to explore the temporal effect of anoxia on gene expression in NMRs and mice, finding that the increased H1.2 expression in NMRs was due to the inhibited *PARP1* expression (**Fig 10**). High expression of H1.2 induced an increase in autophagy under normoxic conditions as well as induced HIF-1α expression under anoxic conditions. In turn, H1.2 overexpression could enhance the dimerization of HIF-1α with HIF-1β, thereby regulating downstream signaling pathways in response to anoxia. Moreover, high H1.2 expression inhibited the migration and invasion of cancer cells as well as in vivo tumor formation, suggesting that the *H1*.*2* gene is a modulator of anoxic adaptation and cancer resistance (**Fig 10**).

## Methods

### ATAC-seq

ATAC-seq was performed according to the approach described previously [61] on 3 MEF and 3 NEF lines at 0 h, 1 h, 2 h, 3 h, and 4 h time points. A total of 50,000 NEF and 50,000 MEF cells were washed twice with 500 μl cold PBS and dissociated in 50 μl lysis buffer (10 mM Tris-HCl (pH 7.4), 10 mM NaCl, 3 mM MgCl_2_, 0.1% (v/v) Nonidet P-40 Substitute). The lysis reaction was terminated by adding a 20-fold large volume of ATAC-RSB reagent, and cell debris was removed. In order to obtain better quality nuclei, NEF and MEF nuclei were centrifuged at 4°C and 500×g at low speed. Nuclei were resuspended in 1× TAG buffer and stained with DAPI. Under the 10× objective of the upright microscope, the morphology of the nuclei was examined and the nuclei were counted. About 1 × 10^5^ nuclei were added to ATAC-transposase reaction reagent (MDTN25U) to capture the open area of chromatin by transposition. The transposition reaction was carried out at 37°C for 30 min. Transposase-acted DNA fragments were purified using MinElute PCR Purification Kit (QIAGEN, cat. #28004), and the purified products were subjected to PCR amplification, and ATAC library adapters were added during the PCR process. In order to determine the number of PCR cycles that need to be performed, the qPCR method was used to confirm the number of cycles through the amplification curve to reduce PCR redundancy. The ATAC library fragments recovered by magnetic bead purification were used to prepare and sequence the single-chain loop library using the BGISEQ500 sequencing platform, and the sequencing category was PE 50 bp reads.

### RNA isolation and library preparation for transcriptomic analysis

Total RNA was isolated using the RNeasy Mini Kit (Qiagen). RNA samples were treated with Dnase I (Promega) at a concentration of 1 unit/μg of total RNA. Total mRNA was purified from 6 μg of total RNA using oligo (dT) magnetic beads. Following purification, the mRNA was fragmented into small pieces using divalent cations under elevated temperature and the cleaved RNA fragments were used for first strand cDNA synthesis using reverse transcriptase and random primers. This was followed by second strand cDNA synthesis using DNA polymerase I and RNaseH. These cDNA fragments then went through an end repair process and ligation of adapters. These products were purified and enriched by PCR to construct a final cDNA library, which was sequenced using the BGISEQ500 sequencing platform with a sequencing class of PE 100 bp reads.

### Protein sample preparation

Cells were harvested with lysis buffer and tip sonication for 10 min. Pelleted by centrifugation and all supernatant was collected. Protein concentration in the lysates was measured by BCA (bicinchoninic acid) using MDParadigm. Briefly, 200 μl 50 mmol/L NH_4_HCO_3_ (Sigma A6141) was mixed with 50 μg protein in Amicon-Ultra-15 (MWCO10kD, Millipore), and the samples were centrifuged at 4°C, 12,000 rcf for 10 min, repeat twice; add 100 μl 50 mmol/L NH_4_HCO_3_ and dithiothreitol (DTT, PlusOne), added up to a final concentration of DTT to 10 mmol/L, followed by incubation at 56°C for 1 h. Subsequently, iodoacetamide (IAA, Sigma) was added (50 mmol/L final concentration) for 40 min and samples were centrifuged at 4°C, 12,000 rcf for 10 min. Then, a new collection tube was used and 100 μl 50 mmol/L NH_4_HCO_3_ was added followed by trypsin (1% final concentration) (V5280, Promega) was mixed with samples and incubation at 37°C for 20 h. The samples were centrifuged at 4°C, 12,000 rcf for 10 min, and 100 μl ddH_2_O was added followed by vortexing, and the samples were centrifuged at 4°C, 12,000 rcf for 10 min, and repeat last operation. After the sample was desalted, the eluted peptides were further purified by vacuum drying. Finally, the peptide mixture was dissolved in a sample solution (0.1% FA, (Sigma)), fully vortexed and the samples were centrifuged at 4°C, 13,200 rpm for 10 min, and the supernatant was transferred to a new tube for label-free quantification by absolute protein abundance.

### Nano LC-MS/MS analysis

Prior to the analyses, peptides were dissolved in 10 μl of 0.1% formic acid. LC-MS/MS was performed on an Orbitrap Fusion Lumos Tribrid Mass Spectrometer (Thermo Scientific) coupled with EASY-nLC 1100 System. For each sample, 2 μl of volume was loaded onto C18 PepMap100 trapcolumn (300 μm × 5 mm) and then to a Thermo Acclaim PepMap RPLC analytical column (150 μm × 15 cm). A procedure of 120 min gradient for each single-shot analysis was performed as following: 4% to 10% B in 5 min, 10% to 22% B in 80 min, 22% to 40% B in 25 min, 40% to 95% B in 5 min, 95% to 95% B in 5 min (A = 0.1% formic acid in water, B = 0.1% formic acid in 90% acetonitrile). The flow rate was 0.3 μl/min. Data-dependent mode was operated for the mass spectrometer, with a full MS scan (300 to 1,400 *m/z*) and 3 s cycle time was set. The MS spectra were acquired at a resolution of 60,000 with an automatic gain control (AGC) target value of 5 × 10^5^ ions or a maximum integration time of 50 ms. High energy collision dissociation (HCD) with the energy set at 35 NCE was used to perform peptide fragmentation. The MS/MS spectra were acquired in the top 15 or 20 most intense precursors at a resolution of 15,000 with an AGC target value of 1 × 10^4^ ions or a maximum integration time of 35 ms.

### Chromatin accessibility analysis

For ATAC-seq data analysis, low-quality reads and Illumina adapters were removed by NGSQCToolkit (v2.3.3) [62]. The remaining clean reads were mapped to the NMR (Ensembl 94) and mouse genome (Ensembl 94) using Bowtie2 (v2.2.9) [63] with default parameters. We used sambamba (v0.7.0) [64] to remove PCR duplications. Mapped reads from mitochondrial DNA and reads with low mapping quality (MAPQ score <30) were filtered using samtools (v1.7). Peak calling was performed with MACS2 (v1.4.2) [65] with parameters “—shift -100—extsize 200—nomodel -B -g 2.34e9.” To identify differential expression peaks between each time points, we obtained a set of open chromatin peaks in the promoter region (< = 2 kb) and analyzed the differential ATAC-seq peaks between each time points using DiffBind [66] defined by abs(log2FC) > 1 and BH-adjusted FDR < 0.05. Genome annotation was performed with ChIPseeker (v1.26.2) [67] using the “annotatePeak” function. We retrieved orthologous genes between NMRs and mice from biomart to compare the difference peaks in the promoter regions between NEF and MEF at same time points. The locations of ATAC-seq peaks are listed in **S6 Table.**

### Comparative transcriptomic analysis of MEFs and NEFs

For RNA-seq data analysis, low-quality reads and Illumina adapters were first removed by NGSQCToolkit (v2.3.3) [62]. Clean reads were aligned against the reference genome of the NMR (Ensembl 94) and mouse (Ensembl 94) using STAR (v2.7.1a) [68] with default parameters. We used featureCounts (v1.6.5) [69] to quantify gene expression. The RPKM (reads per kilobase of transcript per million mapped reads) values were calculated using edgeR [70]. Finally, the function voom in limma [71] was applied to identify DEGs. We considered the FDR (false discovery rate) cutoff value of <0.05 to identify genes that were significantly differentially expressed between each time points. We retrieved orthologous genes between NMRs and mouse from biomart, and identified genes that were significantly differentially expressed between NEF and MEF at same time points. The counts of RNA-seq are listed in **S7 Table.**

### Proteomic analysis of MEFs and NEFs

Protein identification and quantification was performed using MaxQuant (v1.6.7.0) [72] with parameters “first search peptide: 2 ppm; main search peptide: 4.5 ppm; enzyme: Trypsin/P; max. missed: 2; min. peptide length: 7; variable modifications: acetylation (protein N-terminal) and oxidation (Met); fixed modification: carbamidomethyl (Cys); min. score for modified peptide: 40; FDR: 0.01; match time window: 0.7.” During the database search, mouse peptide sequences were downloaded from UniProt and used. We used DEP (v1.10.0) [73] to identify differentially expressed proteins. We considered the *P* value cutoff value of <0.05 to identify proteins that were significantly differentially expressed between various time points and the proteins that were significantly differently expressed at the same time point between NEF and MEF **(S1 Table)**.

### Bioinformatics analysis

To detect differential patterns between different treatments, we performed principal component analysis (PCA) with FactoMineR (v2.3) [74] and used factoextra (v1.0.7) extract and visualize the results. We used clusterProfiler (v3.16.1) [75] for gene enrichment analysis. The significant GO term and KEGG pathways were selected using Fisher’s exact test, and the *p* value and FDR were used to define the threshold of significance (<0.05). To identify dynamic differences in how NEF and MEF respond to anoxia, we calculated a score [28] based on both the change in abundance over time and the difference in the degree of chromatin openness, mRNA abundance and protein abundance between NEF and MEF:

Score=(1−|A|)×B

where A is the Pearson correlation between the temporal response curves for a given abundance over time between NEF and MEF, and B is the integrated numerical difference between the same curves. Thus, for a certain gene, whose abundance does not correlate over time between species, A will tend to 0. Since B describes the absolute area between the NEF and MEF over time, a high value of B suggests a large difference in abundance for a given gene between the 2 species. To diminish the impact from a potentially large difference in abundance between 2 specific gene in the 2 species, we normalized abundance at each time point and for both conditions with respect to abundance in each species at time point 0 and transformed them to log2 ratios. Thus, the log2 ratio is 0 at the first time point **(S8 Table)**.

### Quantitative real-time PCR (qPCR)

Gene expression assay was performed by qRT-PCR as previously described [76]. Briefly, RNA was isolated from harvested cells with Trizol reagent according to the manufacturer’s instruction (Invitrogen, California, United States of America). Total RNA purity and degradation were checked on 1% agarose gels before proceeding. cDNA synthesis was performed using the M-MLV First Strand Kit (Invitrogen, 28025–021). Each gene was analyzed in duplicate and normalized to the housekeeping gene βactin [77]. Primers used in this study are described in **S9 Table**. The reaction mixture contained 4.5 μl cDNA, 0.5 μl primers (10 μm), 12.5 μl 2× SYBR Premix Ex Taq, and ddH_2_O up to 20 μl. The thermal cycling conditions were: 95°C for 1 min; followed by 40 cycles of 10 s at 95°C, 34 s at 60°C, and 60°C for 1 min. Three biological replicates were used. The relative difference is expressed as the fold change calculated using the 2^-△△CT^ method.

### Cell culture and anoxic treatment

All animal experiments were approved and performed in accordance with guidelines set up by the Institute of Zoology, Chinese Academy of Sciences Committee on Animal Resources. C57BL/6 mice were purchased from SPF (Beijing) Biotechnology Co. Primary MEFs and NEFs were isolated according to the methods reported previously [78]. Then, we used SV40-mediated MEF and NEF immortalization [79]. Immortalized MEFs and NEFs were cultured in DMEM medium containing 10% FBS, 100 units/ml penicillin, and 100 mg/ml streptomycin at 5% CO_2_ and 20% O_2_ and as previously optimized [13], MEFs and NEFs were cultured in 37°C and 32°C, respectively. For anoxic conditions, MEFs were maintained under 95% N_2_, 5% CO_2_, and 0% O_2_ at 37°C. NEFs were maintained under 95% N_2_, 5% CO_2_, and 0% O_2_ at 32°C. Cell lines were used at early passages (<12–15 population doublings).

### Cell proliferation assay

For the cell proliferation assay, 1 × 10^4^ MEF cells and MEF cells that overexpression of H1.2 were plated into 96-well plates, cultured in DMEM medium containing 10% fetal bovine serum. Control cells continue to culture in a normoxic incubator for 1 h, 2 h, 3 h, and 4 h, and the experimental cells were transferred to a anoxia workstation (0% O_2_, 5% CO_2_) for 1 h, 2 h, 3 h, and 4 h. After treated as indicated for 1 h, 2 h, 3 h, and 4 h, cells were co-incubated with 10 μl of WST-1 reagent (Beyotime, C0026L) and incubate for 2 h. Absorbance was measured at 450 nm with a microplate reader (Thermo Scientific, Waltham, Massachusetts, USA).

### Plasmid construction

All plasmids were transfected with Lipofectamine 3000 (Life Technologies-Invitrogen, USA) according to the manufacturer’s instructions. cDNAs of *LDHA*, *RBM39*, *PARP1*, and *H1*.*2*, *HIF1α* and its fragments (1-71aa; 71-272aa; 38-384aa; 1-384aa; 71-384aa; 182-384aa; 385-594aa; 595-838aa) were generated from MEF cDNA via PCR amplification and then cloned into different vector. cDNAs of *RBM39* and *PARP1* were cloned into PRK-flag vector; cDNAs of *H1*.*2* was cloned into pEGFP-N1vector; cDNAs of *LDHA*, *HIF1α* and its fragments were cloned into pCSII-MT vector. Site-specific mutations of H1.2-385-594aa and H1.2-595-838aa were synthesized by BGI. Cloning primers are listed in **S10 Table**.

### Overexpression and knockdown of H1.2 in MEF cells

Mouse histone HIST1H1C/H1.2 cDNA were amplified from MEF cDNA via PCR amplification and then cloned into pEGFP-N1vector. pEGFP-N1 or pEGFP-N1- HIST1H1C was transfected into MEF cells. After 48 h of transfection, cells were collected for qPCR and western blot analysis. Mouse H1.2 shRNA were designed and cloned into PLKO.1-puro vector, PLKO.1-puro-shH1.2 plasmids were co-transfected with lentiviral vectors psPAX2 (Addgene#12260) and pMD2.G (Addgene #12259) into HEK293 cells. The viral particles were collected at 48 h and 72 h after transfection and concentrated by ultracentrifugation at 19,400 *g* for 2.5 h. Add viral particles into MEF cells for 48 h, then add puromycin to screen for positive cells, after cells up to 90% confluence collected for qPCR and western blot analysis.

### Autophagy analysis

To assess autophagy, NEF, MEFs, MEFs with overexpression H1.2, and MEFs with knock down H1.2 were transfected with plasmid encoding GFP-RFP-LC3 for 24 h. The cultured cells were fixed with 4% paraformaldehyde for 30 min, followed by DAPI staining, and imaged with an Olympus FV1000 Viewer confocal microscope.

### Co-immunoprecipitation (Co-IP)

The Co-IP experiments were performed as protocol described previously [80]. Briefly, HEK293T cells were transfected with pEGFP-N1 and pEGFP-N1- H1.2 plasmids, collected and lysed in IP lysis solution (containing 50 mM Tris-HCl, 150 mM NaCl, 1% Triton-100, 1 mM EDTA, 1 mM PMSF, 5 μg/ml Aprotinin, and 5 μg/ml Leupeptin at pH 7.5) at 4°C for 2 h, following which the samples were centrifuged at 16,000 *g* at 4°C for 10 min. The supernatants were collected and mixed with anti-GFP(A) beads (KT, KTSM1301) and rotated 3 h at 4°C. After centrifugation at 1,200 *g* at 4°C for 3 min, the supernatant was discarded and the beads were washed with PBST buffer 3 times. After centrifugation, the beads were collected, mixed with 1× SDS loading buffer and denatured at 95°C for 10 min and centrifugation at 1,200 *g* at 4°C for 3 min, then the samples preparation for western blot analysis.

### Western blot analysis

Cells were lysed using RIPA Lysis and Extraction Buffer (Thermo Fisher, 89901) at 4°C for 30 min, following which the samples were centrifuged at 16,000 *g* at 4°C for 10 min. The supernatants were collected mixed with 5× SDS loading buffer and denatured at 95°C for 10 min. Protein samples concentration was measured by BCA kit. About 15 g protein per sample was subjected to SDS-PAGE and electrotransferred to a PVDF membrane (Bio-Rad, LG1620177). The membrane was blocked with 5% blotting grade blocker (Bio-Rad, 1706404) for 1 h and incubated with primary antibodies overnight at 4°C, then with horseradish peroxidase (HRP)-conjugated secondary antibodies. The visualization and data processing were performed by chemiluminescent HRP substrate (Millipore, WBKIS0100) with ChemiDoc XRS system (Bio-Rad). Antibodies used in this study were as follows: anti-H1.2 (Santa Cruz, sc-49712), anti-PARP1 (Cell Signaling Technology, #9718), anti-HIF1α (Abclonal, A16873), anti-HIF1β (Abclonal, A14705), anti-LC3B (Abclonal, A19665), anti-ATG3 (Abclonal, A19700), anti-ATG5 (Abclonal, A0203), anti-P62 (Cell Signaling Technology, #2947), anti-NRF2 (Abclonal, A16873), anti-Myc (Abclonal, AE010), anti-Flag (Sigma, F1804), anti-HA (Abclonal, AE008), anti-Tubulin (Santa Cruz, sc-69879), and anti-GFP (Sigma, G8795).

### Immunofluorescence

A549-oe-gfp-H1.2, B16F10-oe-gfp-H1.2, A549-WT, and B16F10-WT in cell slides were washed 3 times with PBS. Next, the cells were fixed with 4% paraformaldehyde for 5 min. The cells slides were washed with PBS 3 times, treated with 0.5% Triton X-100 for 5 min, washed with PBS 3 times again and finally blocked with 5% bovine serum albumin for 30 min. All samples were incubated with corresponding primary antibodies (anti-NRF2 (1:200 dilution, Abclonal), anti-P62 (1:200 dilution, Abclonal), anti-LC3 (1:200 dilution, Abclonal)) at 4°C overnight, after washed with PBS 3 times, incubation with secondary antibodies for 1 h at 37°C, followed by washed with PBS and DAPI staining. Images were taken using LSM780 (Zeiss) microscopes.

### LC-MS*/*MS analysis and protein identification

The eluted proteins from Co-IP were separated on a 12% SDS-PAGE gel and stained with Coomassie brilliant blue. After decolouration, the gel slice containing proteins of interest was cut and subjected to dehydration (in 100% acetonitrile), reduction (with 10 mM DTT in 25 mM NH_4_HCO_3_ for 45 min at 56°C), and alkylation (with 40 mM iodoacetamide in 25 mM NH_4_HCO_3_ for 45 min at RT in the dark) [16,28]. Proteins were then digested into peptides by sequencing grade trypsin (Worthington) overnight at 37°C. The resultant peptides were homogenized in 0.1% formic acid and separated by the online Easy-nLC 1000 system (Thermo Fisher Scientific) with a C18 reverse-phase column. The column was eluted with a linear gradient of 5% to 30% acetonitrile in 0.2% formic acid at a rate of 300 nl*/*min for 100 min. The mass spectra were acquired by nanoLC-Q EXACTIVE (Thermo Fisher Scientific) equipped with a nano-ES ion source (Proxeon Biosystems). Full scan spectra (from *m/z* 300 to 1,600) were acquired in the Orbitrap analyzer with a resolution of 60,000 at 400 *m/z* after the accumulation of 1,000,000 ions. The 5 most intense ions in each scan were selected for collision-induced dissociation (CID) fragmentation in the linear ion trap after 3,000 ions were accumulated. The maximal filling time was set at 500 ms for the full scans and 150 ms for the MS*/*MS scans. The dynamic exclusion list was defined as a maximum of 500 entries with a maximum retention period of 60 s and a relative mass window of 10 ppm. The raw files were processed using MaxQuant software (v1.3.0.5). The generated peak list files were analyzed with Thermo Proteome Discoverer (1.4.0.288) based on theUniprot_proteome_human/mouse/Heterocephalus glaber (update_20171001). The parameters for analyses were set as follows: trypsin enzyme; up to 2 missed cleavages; alkylated cysteine as fixed modification; oxidized methionine as variable modifications. MS tolerance was 10 ppm while MS*/*MS tolerance was 0.02 Da. The required FDR was set to 1% at peptide and protein levels, and the minimum length for the acquired peptide was set to 7 amino acids. At least 2 unique peptides (peptides that only match 1 protein sequence) or razor peptides (peptides that might match to more than 1 protein sequences) per protein group was required for protein identification.

### Wound healing assay

The migratory potential of overexpression PEGFP-N1 and PEGFP-N1-H1.2 A549 cells that were maintained in DMEM medium under normoxic conditions for up to 48 h was assessed using the wound healing assay as described previously with little modification [81]. The wound areas on cell monolayers were created using a sterile 200 μl pipette tip.

### Cell migration assay

Migration assay was determined using 24-well modified Boyden chambers with 8-μm pore polycarbonate filters (Corning Costar, Cambridge, Massachusetts, USA) in accordance with the manufacturer’s instructions; 5 × 10^4^ cells were seeded per well in the upper well of the migration chamber in DMEM without serum, the lower chamber well contained DMEM supplemented with 30% FBS to stimulate cell migration. After incubation for 24 h, noninvading cells were removed from the top well with a cotton swab while the bottom cells were fixed with absolute methanol for 30 min, stained with 0.1% crystal violet for 1 h, and photographed in 3 independent fields for each well. Three independent experiments were conducted in triplicate.

### Cell invasion assay

Cell invasion assay was determined using 24-well modified Boyden chambers with 8-μm pore polycarbonate filters (Corning Costar, Cambridge, Massachusetts, USA) were pre-coated with Corning Matrigel Matrix (Cat. No. 354230. Corning, Tewksbury, Massachusetts, USA) in accordance with the manufacturer’s instructions; 5 × 10^4^ cells were seeded per well in the upper well of the invasion chamber in DMEM without serum, the lower chamber well contained DMEM supplemented with 30% FBS to stimulate cell invasion. After incubation for 24 h, noninvading cells were removed from the top well with a cotton swab while the bottom cells were fixed with absolute methanol for 30 min, stained with 0.1% crystal violet for 1 h, and photographed in 3 independent fields for each well. Three independent experiments were conducted in triplicate.

### In vivo tumor growth

Female BALB/c mice (6 weeks old) were purchased from SPF (Beijing) Biotechnology Co. All animal handling and experimental procedures were approved and performed in accordance with guidelines set up by the Institute of Zoology, Chinese Academy of Sciences Committee on Animal Resources. B16F10 cells stably expressing H1.2 or WT were injected subcutaneously into both BALB/c mice (3 × 10^6^ cells in 100 μl). Tumor sizes were measured using vernier caliper every day when the tumors were apparently seen and tumor volume was calculated according to the formula: volume = 0.5 × Length × Width^2^. When tumors reached the maximum legal size allowed, mice were killed, tumor sizes measured and tumor samples were formalin-fixed, paraffin-embedded, and sectioned at 5 μm for VEGF (Santa Cruz, California, USA) immunohistochemical staining under the standard procedure as described before [82].

### Immunohistochemical staining

The WT-C57 and Mus-H1.2-C57 tumor tissues were fixed with 4% paraformaldehyde, dehydrated in different gradient ethanol solutions, followed by embedded in paraffin for slicing into 5 μm slides. The antibodies used for immunohistochemistry were anti-NRF2 (1:200 dilution, Abclonal), anti-P62 (1:200 dilution, Abclonal), anti-LC3 (1:200 dilution, Abclonal). Images were taken using KEYENCE microscopes.

### Hematoxylin-eosin staining

Samples of expressing H1.2 or WT B16F10 tumor were fixed in 4% formalin for 48 h and processed and embedded into paraffin blocks according to routine procedures. Briefly, sections were deparaffinized in xylene (2 × 5 min) and rehydrated with successive 1-min washes in 100%, 96%, 80%, and 70% ethanol. They were then stained with hematoxylin (2 min), rinsed with distilled water, rinsed with 0.1% hydrochloric acid in 50% ethanol, rinsed with tap water for 15 min, stained with eosin for 1 min, and rinsed again with distilled water. The slides were then dehydrated with 95% and 100% ethanol successively followed by xylene (2 × 5 min) and mounted with coverslips [83].

### Generation and characterization of MusH1.2 transgenic mice

Transgenic (Tg) mice with C57BL background were generated as previously reported [47,48]. The generation of transgenic mice expressing Mus H1.2 was conducted by microinjection as previously described [47]. For expression of Mus H1.2, a 639 bp cDNA of the (NM_015786) Mus H1.2 gene was cloned into PCAGGS-mCherry. The resultant fragment was injected into fertilized mouse zygotes and then transplanted into recipient mice. The transgenic mice were confirmed by PCR and then bred into F1 progeny. Non-Tg C57BL/6 mice were purchased from SPF (Beijing) Biotechnology Co. Six-week-old Tg and non-Tg mice were used for the current study. Both Tg mice and non-Tg mice were transferred to hypoxia workstation (4% O_2_) for experiment.

### Statistics

The statistical comparisons, unless otherwise stated, were mainly conducted using the Mann–Whitney U test. A *P* value of less than 0.050 was considered statistically significant, with the following notations: not significant (n.s., *P* > 0.050), *P < 0.050, **P < 0.010, ***P < 0.001. Moreover, adjustments for multiple testing were taken into account. All experiments were performed independently at least 3 times, and data are represented as mean ± standard deviation (SD). In Fig 3J, we initially applied repeated measures ANOVA to analyze data that included 3 cell types (MEF/H1.2, MEF/shH1.2, MEF/Control) and 2 time points (0 h and 4 h). The analysis revealed that the main effect of cell type was significant (F value = 233.988, *P* = 0.004, S11 Table), as was the main effect of time (F value = 40.24, *P* = 0.002). The interaction effect was also significant (F value = 8.109, *P* = 0.039, S11 Table). In Fig 5D and 5E, we employed two-way ANOVA to facilitate proper comparison not only between the cell lines but also across conditions of normoxia and anoxia. The results indicated a significant main effect of cell line (Fig 5D and 5F value = 367.5, *P* < 0.0001; Fig 5E and 5F value = 286.74, *P* = 1.50E-07, S12 Table) and a significant main effect of condition/time (Fig 5D and 5F value = 430.2, *P* = 3.068E-08; Fig 5E and 5F value = 951.05, *P* = 1.33E-09, S12 Table). Furthermore, the interaction effect was significant (Fig 5D and 5F value = 182.6, *P* < 0.0001; Fig 5E and 5F value = 25.65, *P* = 0.001, S12 Table). Additional methodologies and experimental details are available in the S1 Data and S2 Data.

### Ethics statement

Samples were obtained with informed consent and all protocols were approved by Institute of Zoology, Chinese Academy of Sciences Ethics Review Board (Scientific and Research Ethics Committee, IOZ-IACUC-2021-149, IOZ-IACUC-2022-136).

## Supporting information

S1 Fig Apoptosis of MEFs and NEFs treated with anoxia at different times. **(A)** Microscopy analysis of the live/dead assay for NEFs and MEFs were exposed at the 1-, 2-, 3-, and 4-h time points under anoxic conditions. Live cells fluoresce green. Necrotic or dead cells containing nucleic acid-bound 7-AAD fluoresce red. **(B)** Quantification data for live/dead assay for NEFs and MEFs were exposed at the 1-, 2-, 3-, and 4-h time points under anoxic conditions. *P < 0.05, **P < 0.01, and ***P < 0.001. The data underlying the graphs shown in the figure can be found in S2 Data.
(TIF)

S2 Fig Pearson correlation analyses of RNA-seq data and proteomics. **(A)** Matrix representation of Pearson correlation values of the mRNA of each of the replicates compared to every other replicate, 3 at a time, for both NEFs and MEFs. Correlations are indicated in the color gradient to the right of each plot (*r* > 0.9700, Pearson). **(B)** Matrix representation of Pearson correlation values of the proteins of each of the replicates compared to every other replicate, 3 at a time, for both NEFs and MEFs. Correlations are indicated in the color gradient to the right of each plot (*r* > 0.9300, Pearson). The data underlying the graphs shown in the figure can be found in S2 Data.
(TIF)

S3 Fig Pearson component analysis of RNA-seq data and proteomics data. **(A)** PCA plot using the normalized counts for the RNA-seq data between NEFs and MEFs at the 1-, 2-, 3-, and 4-h time points under anoxic conditions. The PCA explain 62.4% (PC1), 9.5% (PC2), and 6.5% (PC3) of total variance. **(B)** PCA plot using the normalized counts for the proteome data between NEFs and MEFs at the 1-, 2-, 3-, and 4-h time points under anoxic conditions. The PCA explain 36.1% (PC1), 7.6% (PC2), and 3.9% (PC3) of total variance. The data underlying the graphs shown in the figure can be found in S2 Data.
(TIF)

S4 Fig Temporal profiling of anoxic response in mRNA. **(A)** Heatmap illustrating the temporal changes between NEFs and MEFs at the 1-, 2-, 3-, and 4-h time points under anoxic conditions. Genes were divided into 4 groups based on relative expression under anoxic conditions compared with under normoxic conditions. **(B)** GO terms of biological process (BP) for each cluster. *X*-axis represents enrichment and the size depicts the log_10_ (*p* value) of each GO term; *y*-axis represents the different BP terms. Data were scaled across rows before mapping to colors. The data underlying the graphs shown in the figure can be found in S2 Data.
(TIF)

S5 Fig Temporal profiling of anoxic response in proteome. **(A)** Protein density plots showing the distribution of log_2_ ratios for each time point relative to normoxia for both NEFs and MEFs. **(B)** Volcano plot of the identified proteins for each time point and both NEFs and MEFs. Each point represents difference in the expression (fold change) relative to normoxia (time = 0) plotted against the level of statistical significance (*q* values). Different colors indicate density of data points. **(C)** Heatmap illustrating temporal changes between NEFs and MEFs at 1-, 2-, 3-, and 4-h time points under anoxic conditions. Genes were divided into 4 groups based on the relative expression under anoxic conditions compared with that under normoxic conditions. **(D)** GO terms of biological process (BP) for each cluster. *X*-axis represents enrichment and the size depicts the log_10_ (*p* value) of each GO term; *y*-axis represents the different BP terms. Data were scaled across rows before mapping to colors. **(E)** GO terms of molecular function (MF) for each cluster. *X*-axis represents enrichment and the size depicts the log_10_ (*p* value) of each GO term; *y*-axis represents the different BP terms. Data were scaled across rows before mapping to colors. **(F)** KEGG pathways for each cluster. *X*-axis represents enrichment and the size depicts the log_10_ (*p* value) of each pathway; *y*-axis represents the different KEGG pathways. Data were scaled across rows before mapping to colors. The data underlying the graphs shown in the figure can be found in S2 Data.
(TIF)

S6 Fig The absolute integral shown as density plot. The density is plotted in x-axis and the integral is plotted on the y-axis. Box plots including Turkey’s 5 numbers for proteins according to the score (A) or the absolute integral score (B). Totally identified 667 proteins shown significant scores changes, had a score >0.87, corresponding to the 75th percentile. The data underlying the graphs shown in the figure can be found in S2 Data.
(TIF)

S7 Fig Temporal profiling of SH3BGRL3 in RNA-seq data, proteomics, and ATAC-seq data. **(A)** RNA level of *Sh3bgrl3* in NEFs and MEFs according to RNA-seq analysis. **(B)** Protein level of *SH3BGRL3* in NEFs and MEFs according to proteomics analysis. **(C)** Chromatin opening peaks of *Sh3bgrl3* in NEFs and MEFs according to ATAC-seq analysis. The data underlying the graphs shown in the figure can be found in S2 Data.
(TIF)

S8 Fig Expression levels of *H1*.*2* in tissues of different mammals. **(A)** *H1*.*2* mRNA expression levels in tissues of different mammals. Brain (purple), kidney (green), and liver (orange). *H1*.*2* were highly expressed in the liver, kidney, and brain of naked mole rats compared with other mammals. The data underlying the graphs shown in the figure can be found in S2 Data.
(TIF)

S9 Fig The expression of genes related to the glycolytic pathway in MEF-oe-H1.2 and MEF-WT cell. Different colors reflect the change of gene expression of MEF-oe-H1.2 compared with MEF-WT at 0, 1, and 4 h. The gene names represented by the numbers are listed at the bottom of the figure.
(TIF)

S10 Fig The expression of genes related to the carbon metabolism pathway in MEF-oe-H1.2 and MEF-WT cell. Different colors reflect the change of gene expression of MEF-oe-H1.2 compared with MEF-WT at 0, 1, and 4 h.
(TIF)

S11 Fig The protein expression of PKM, LDHA, GLUT1, HK2, and HK1 in NEF, MEF-oe-H1.2, and MEF-WT cells. Western blot analysis of PKM, LDHA, GLUT1, HK2, and HK1 expressions in NEF, MEF-oe-H1.2, and MEF-WT cells.
(TIF)

S12 Fig Results of H1.2 transgenic mice. **(A)** RT-PCR results of *H1*.*2* in heart, liver, spleen, lung, kidney, muscle, and tail of C57-oe-MusH1.2 and C57-WT. **(B)** Breathing time of WT-C57 and C57-oe-MusH1.2 male mice under 4% O_2_ conditions. The data underlying the graphs shown in the figure can be found in S2 Data.
(TIF)

S13 Fig Results of energy expenditure in C57-WT and C57-oe-MusH1.2. Daily energy expenditure of WT-C57 and C57-oe-MusH1.2 male mice. The data underlying the graphs shown in the figure can be found in S2 Data.
(TIF)

S14 Fig Transcriptome and proteome profiling, Open chromatin and Transcriptome profiling of anoxic responses in NEF and MEF. Correlation of log-transformed protein intensities and log-transformed FPKM (fragments per kilobase per million mapped reads) values (RNA intensities) from MEF and NEF for each time point. The data underlying the graphs shown in the figure can be found in S2 Data.
(TIF)

S15 Fig Results of H1.1, H1.3, H1.4, and H1.5 expressed in NEF and MEF. **(A)** RNA level of H1.1 in NEFs and MEFs according to RNA-seq analysis. Times 0 represent normoxic condition, times 1, 2, 3, 4 represent at 1, 2, 3, and 4 h time points under anoxic conditions, respectively, hereinafter inclusive. **(B)** RNA level of H1.3 in NEFs and MEFs according to RNA-seq analysis. **(C)** RNA level of H1.4 in NEFs and MEFs according to RNA-seq analysis. **(D)** RNA level of H1.5 in NEFs and MEFs according to RNA-seq analysis. The data underlying the graphs shown in the figure can be found in S2 Data.
(TIF)

S16 Fig Results of H1.1, H1.3, H1.4, and H1.5 in overexpression H1.2 MEFs and WT MEFs. **(A)** RNA level of H1.1 in overexpression H1.2 MEFs and WT MEFs according to RNA-seq analysis. Times 0 represent normoxic condition, times 1, 2, 3, 4 represent at 1, 2, 3, and 4 h time points under anoxic conditions, respectively, hereinafter inclusive. **(B)** RNA level of H1.3 in overexpression H1.2 MEFs and WT MEFs according to RNA-seq analysis. **(C)** RNA level of H1.4 in overexpression H1.2 MEFs and WT MEFs according to RNA-seq analysis. **(D)** RNA level of H1.5 in overexpression H1.2 MEFs and WT MEFs according to RNA-seq analysis. The data underlying the graphs shown in the figure can be found in S2 Data.
(TIF)

S1 Table Differential expression analysis of epigenetics, transcriptomics, and proteomics in NEFs and MEFs in the present study. ^1^ Log fold change of NEF protein in anoxia 1 h compared with 0h. ^2^ *P* value of NEF protein in anoxia 1 h compared with 0 h. ^3^ Log fold change of NEF RNA in anoxia 1 h compared with 0 h. ^4^ *P* value of NEF RNA in anoxia 1 h compared with 0 h. ^5^ Log fold change of NEF open chromatin in anoxia 1 h compared with 0 h. ^6^ *P* value of NEF open chromatin in anoxia 1 h compared with 0 h. ^7^ There is no value.
(XLSX)

S2 Table Overlap in the DEGs identified among the RNA-seq and proteomics data in NEFs. ^1^ Overlap gene name in the DEGs identified among the ATAC-seq, RNA-seq, and proteomics data in NEFs. ^2^ Log fold change of NEF RNA in anoxia 1 h compared with 0 h. ^3^ Log fold change of NEF protein in anoxia 1 h compared with 0 h. ^4^ There is no value.
(XLSX)

S3 Table Overlap in the DEGs identified among the RNA-seq and proteomics data in MEFs. (XLSX)

S4 Table Genes with known functions in hypoxia. ^1^ Score for each gene were calculated using the following equation: score = (1 − |*A*|) × *B*, where *A* is the Pearson correlation between the temporal response curves for a given protein over time in NEFs and MEFs, and *B* is the integrated numerical difference between the same curves.
(XLSX)

S5 Table KEGG pathways among genes with known functions in hypoxia. (XLSX)

S6 Table The difference peaks in the promoter regions between NEF and MEF at same time points. ^1^ Mus represent Mus _musculus. ^2^ Nmr represent Heterocephalus_glaber. ^3^ Peak counts of MEF open chromatin in anoxia 1 h compared with 0 h.
(XLSX)

S7 Table The gene expression normalized value of NEFs and MEFs. ^1^ The gene expression normalized value at each time point in NEFs and MEFs.
(XLSX)

S8 Table Plotted scores of proteins in NEFs and MEFs. ^1^ Scores for all proteins were calculated using the following equation: score = (1 − |*A*|) × *B*, where *A* is the Pearson correlation between the temporal response curves for a given protein over time in NEFs and MEFs, and *B* is the integrated numerical difference between the same curves.
(XLSX)

S9 Table Quantitative real-time PCR primers used in this study. (XLS)

S10 Table Cloning primers used in this study. (XLS)

S11 Table Anova results for Fig 3J. (XLSX)

S12 Table Anova results for Fig 5D and 5E. (XLSX)

S1 Data The individual numerical values in Figs 1C–1F, 2A–2D, 3A–3E, 3J, 4B, 5B, 5D, 5E, 5H, 6A, 6C, 6E, 6H, 7C and 8B–8K. (XLSX)

S2 Data The individual numerical values in S1B, S2A, S2B, S3A, S3B, S4A, S4B, S5A–S5F, S6, S7A–S7C, S8, S12A, S12B, S13, S14, S15A–S15D and S16A–S16D Figs. (XLSX)

S1 Raw Images Raw images. (PDF)

## Abbreviations

AGC: automatic gain control
BCA: bicinchoninic acid
bHLH: basic helix–loop–helix
CID: collision-induced dissociation
DEG: differentially expressed gene
FDR: false discovery rate
HCD: high energy collision dissociation
HMM-HA: high molecular weight hyaluronic acid
HRP: horseradish peroxidase
MEF: mouse embryonic fibroblast
NEF: NMR embryonic fibroblast
NMR: naked mole rat
MS: mass spectrometry
PCA: principal component analysis
RT-PCR: reverse transcription-polymerase chain reaction
SD: standard deviation
snRNA-seq: single-nucleus RNA sequencing
WT: wild-type
