# Supplementary figures and images for: Comparative time-series multi-omics analyses suggest H1.2 involvement in anoxic adaptation and cancer resistance
Source: PLoS Biol. 2024 Aug 23;22(8):e3002778. doi: 10.1371/journal.pbio.3002778 (PMC11376556; doi:10.1371/journal.pbio.3002778)

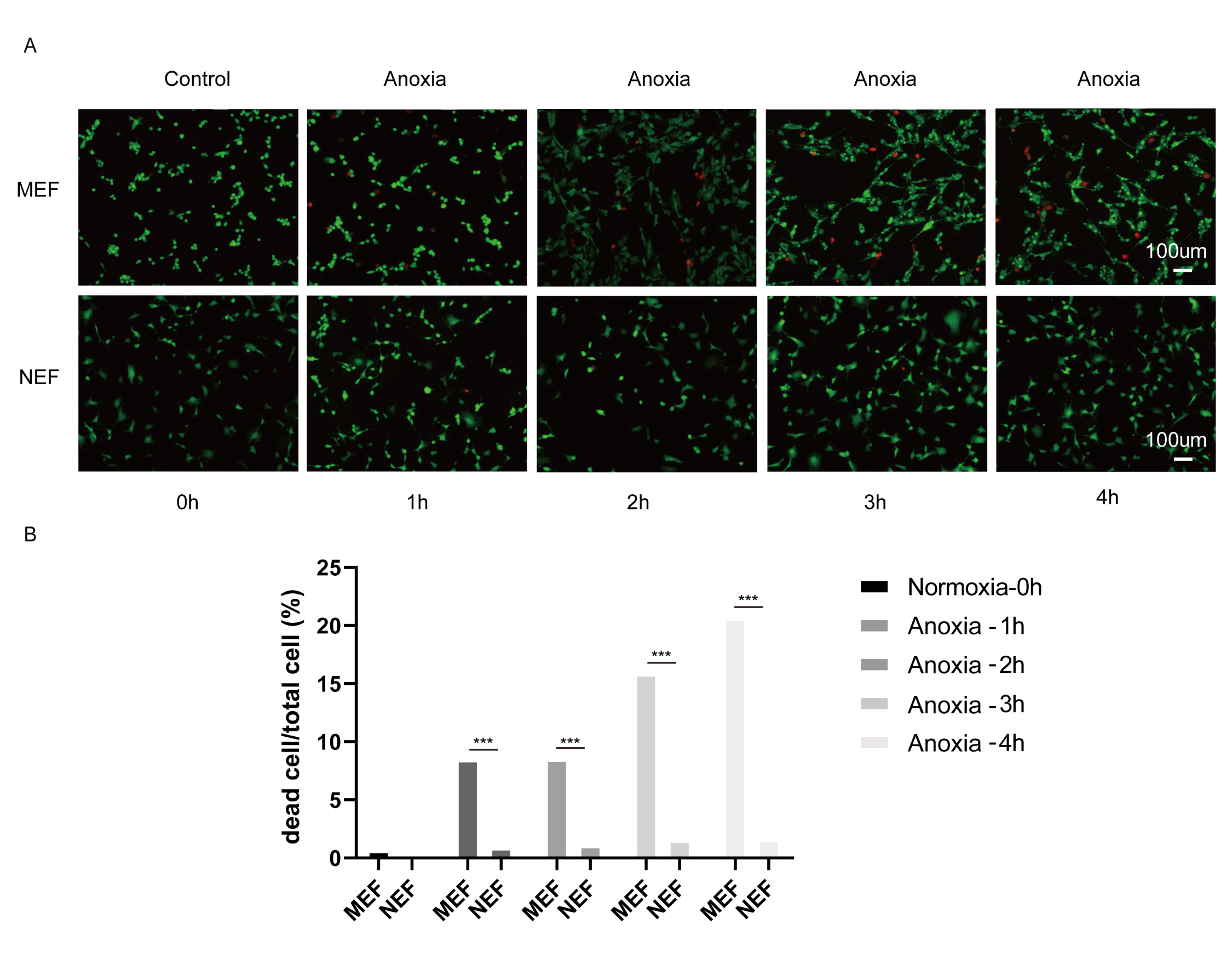

Supplement: S1 Fig — (A) Microscopy analysis of the live/dead assay for NEFs and MEFs were exposed at the 1-, 2-, 3-, and 4-h time points under anoxic conditions. Live cells fluoresce green. Necrotic or dead cells containing nucleic acid-bound 7-AAD fluoresce red. (B) Quantification data for live/dead assay for NEFs and MEFs were exposed at the 1-, 2-, 3-, and 4-h time points under anoxic conditions. *P < 0.05, **P < 0.01, and ***P < 0.001. The data underlying the graphs shown in the figure can be found in S2 Data. (TIF) [file pbio.3002778.s001.tif]

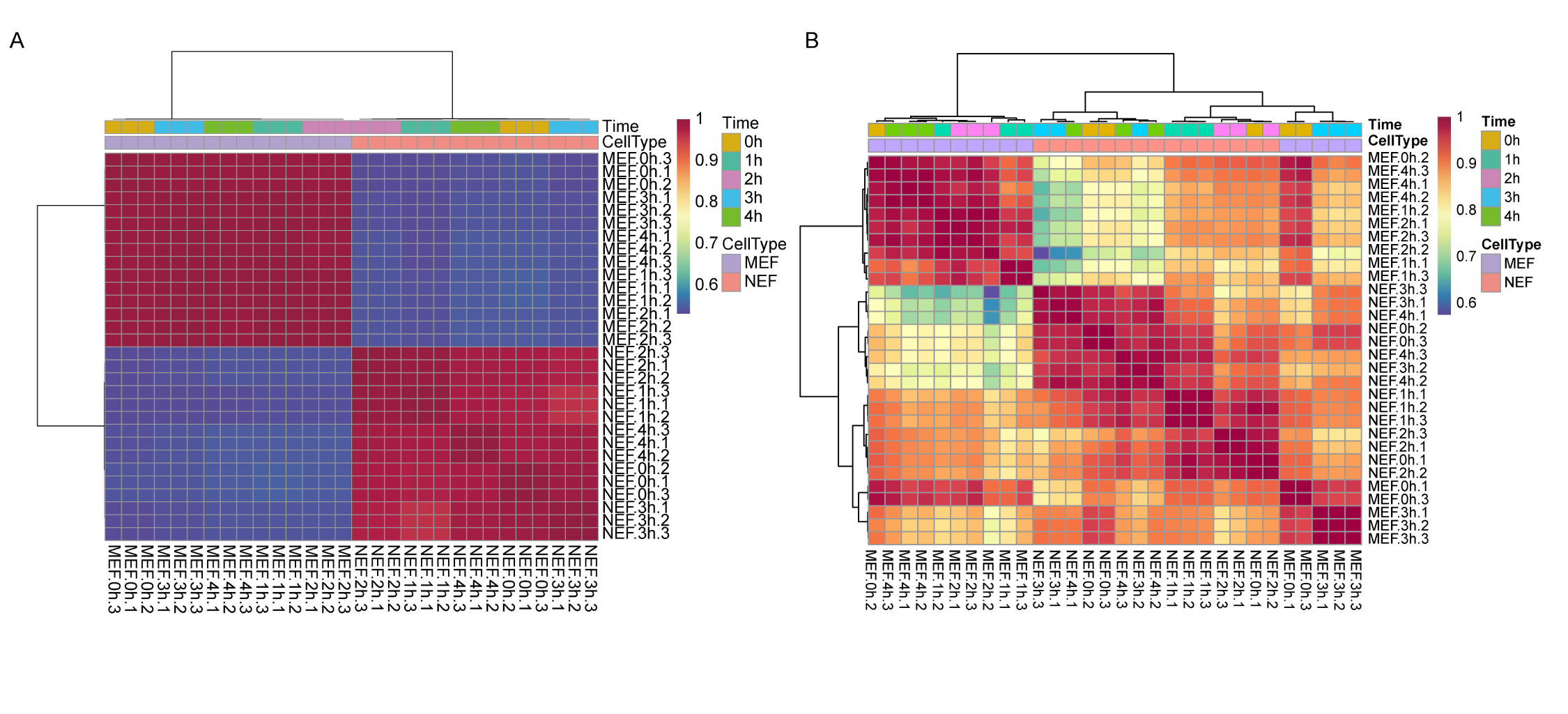

Supplement: S2 Fig — (A) Matrix representation of Pearson correlation values of the mRNA of each of the replicates compared to every other replicate, 3 at a time, for both NEFs and MEFs. Correlations are indicated in the color gradient to the right of each plot (r > 0.9700, Pearson). (B) Matrix representation of Pearson correlation values of the proteins of each of the replicates compared to every other replicate, 3 at a time, for both NEFs and MEFs. Correlations are indicated in the color gradient to the right of each plot (r > 0.9300, Pearson). The data underlying the graphs shown in the figure can be found in S2 Data. (TIF) [file pbio.3002778.s002.tif]

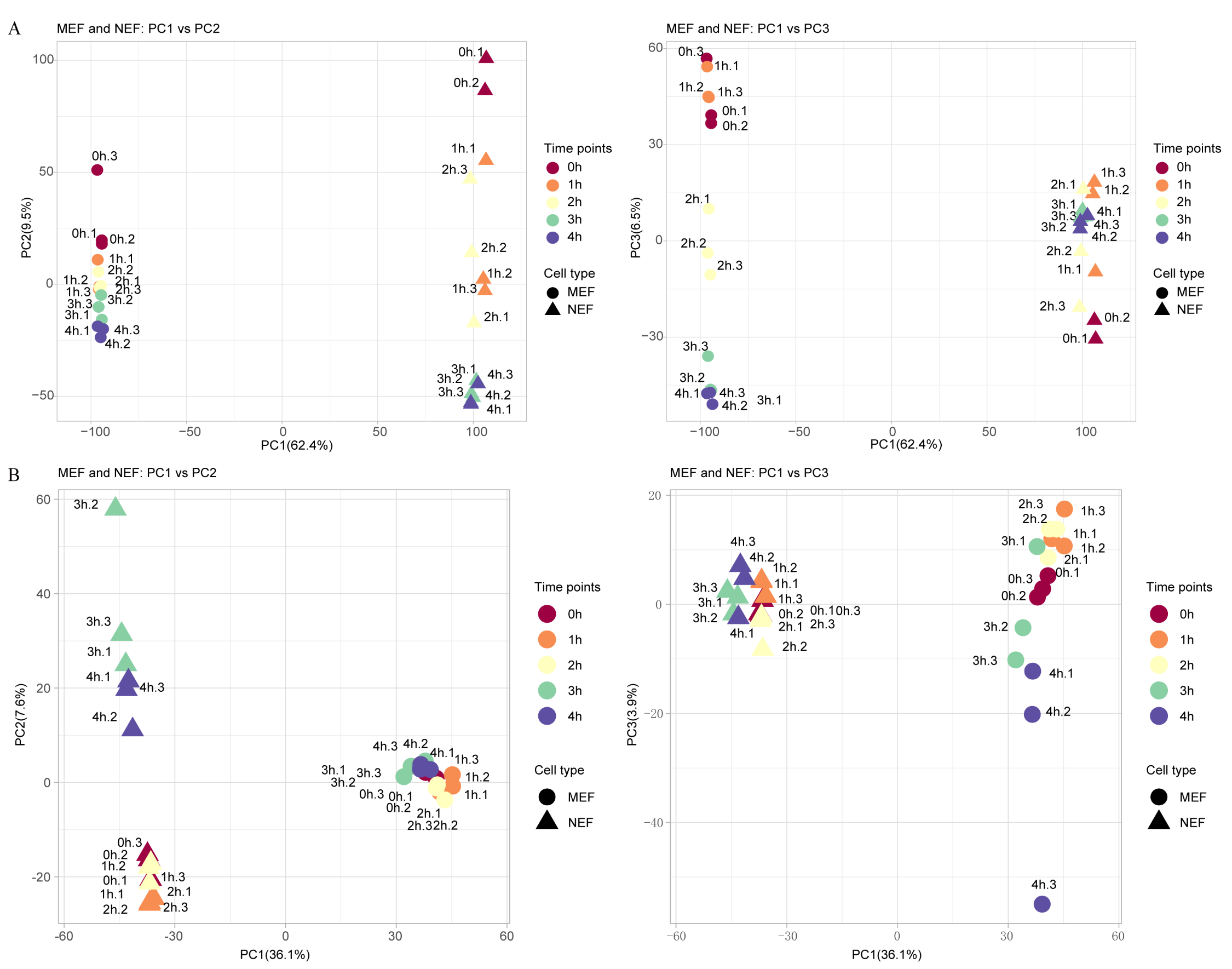

Supplement: S3 Fig — (A) PCA plot using the normalized counts for the RNA-seq data between NEFs and MEFs at the 1-, 2-, 3-, and 4-h time points under anoxic conditions. The PCA explain 62.4% (PC1), 9.5% (PC2), and 6.5% (PC3) of total variance. (B) PCA plot using the normalized counts for the proteome data between NEFs and MEFs at the 1-, 2-, 3-, and 4-h time points under anoxic conditions. The PCA explain 36.1% (PC1), 7.6% (PC2), and 3.9% (PC3) of total variance. The data underlying the graphs shown in the figure can be found in S2 Data. (TIF) [file pbio.3002778.s003.tif]

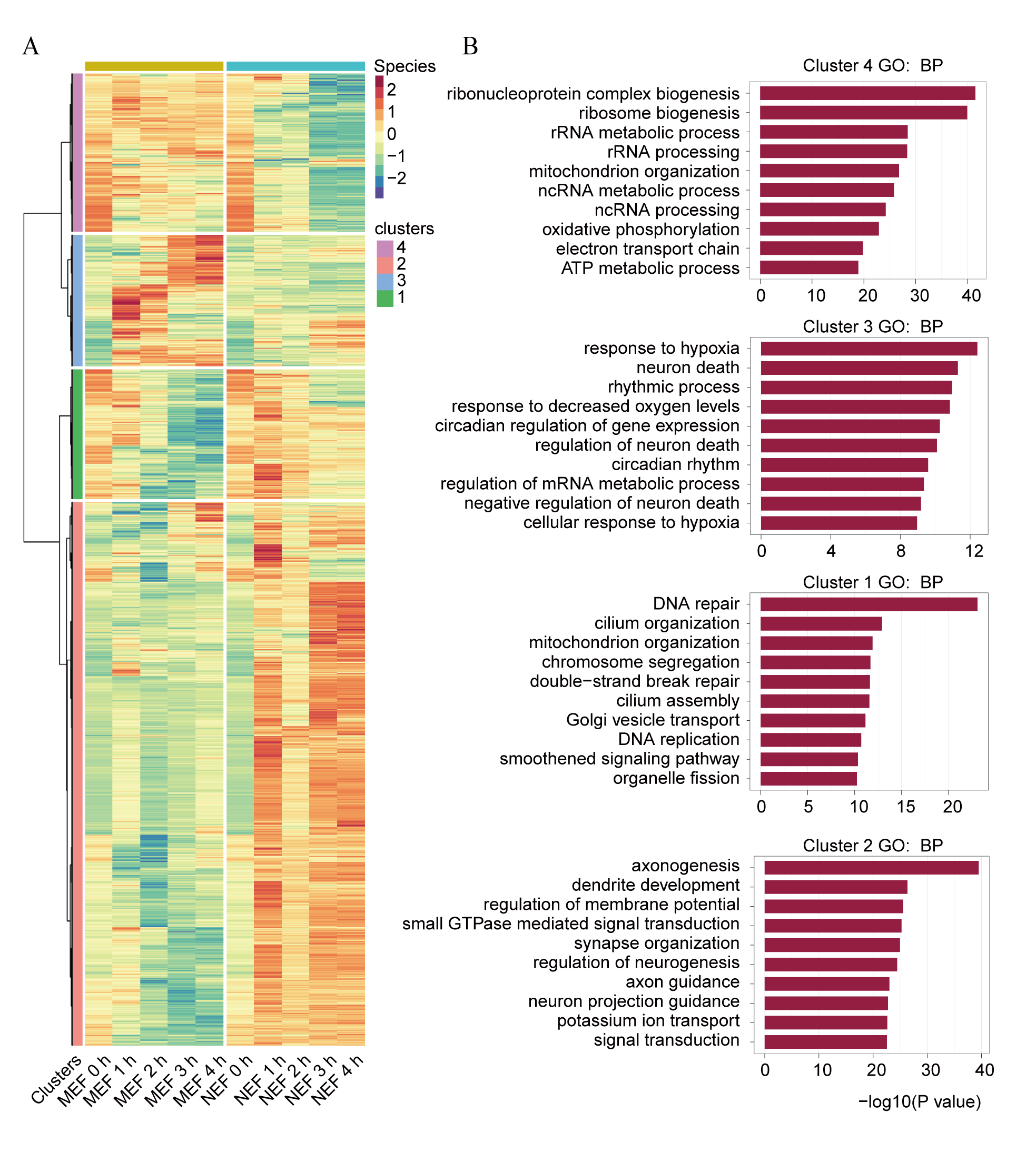

Supplement: S4 Fig — (A) Heatmap illustrating the temporal changes between NEFs and MEFs at the 1-, 2-, 3-, and 4-h time points under anoxic conditions. Genes were divided into 4 groups based on relative expression under anoxic conditions compared with under normoxic conditions. (B) GO terms of biological process (BP) for each cluster. X-axis represents enrichment and the size depicts the log10 (p value) of each GO term; y-axis represents the different BP terms. Data were scaled across rows before mapping to colors. The data underlying the graphs shown in the figure can be found in S2 Data. (TIF) [file pbio.3002778.s004.tif]

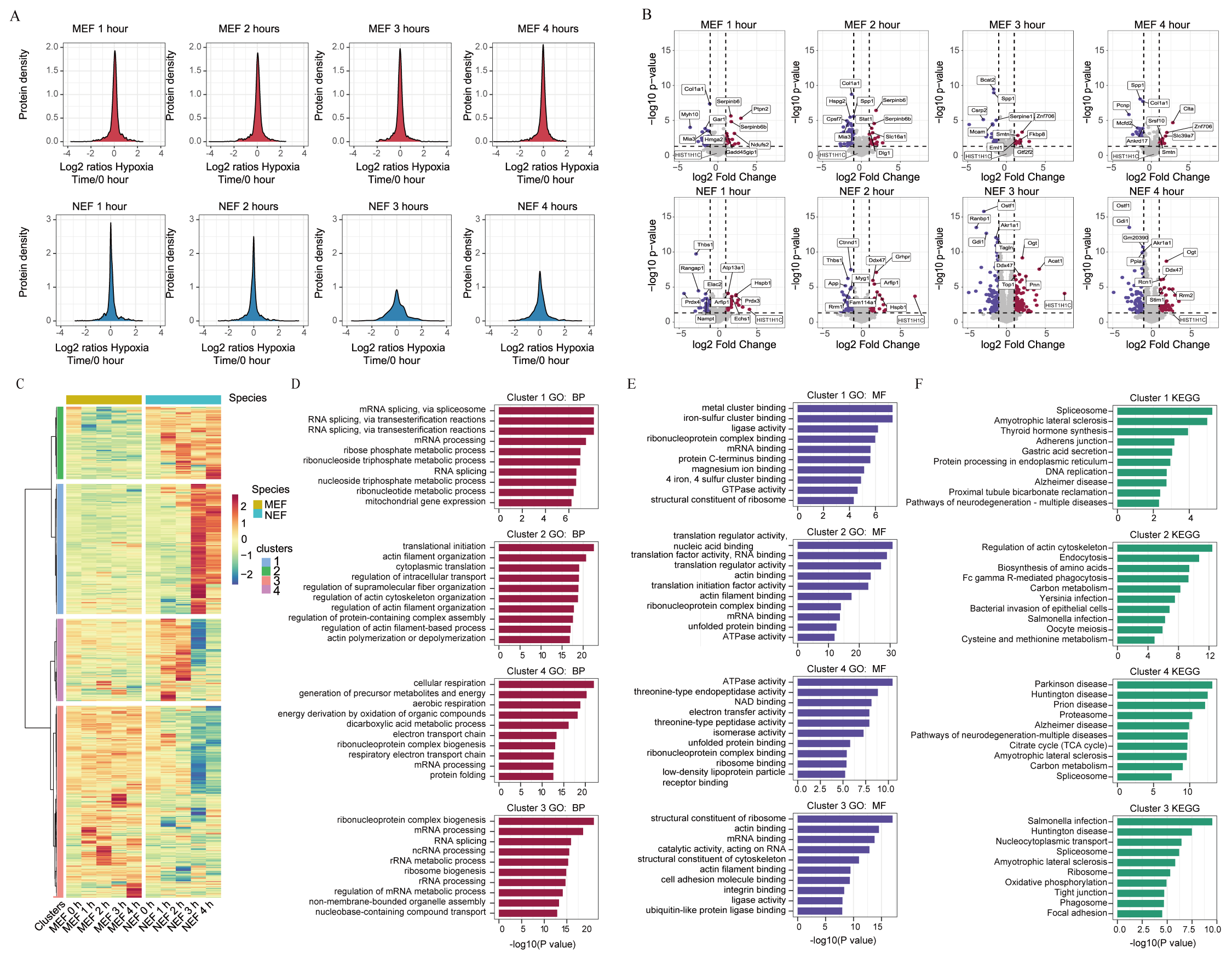

Supplement: S5 Fig — (A) Protein density plots showing the distribution of log2 ratios for each time point relative to normoxia for both NEFs and MEFs. (B) Volcano plot of the identified proteins for each time point and both NEFs and MEFs. Each point represents difference in the expression (fold change) relative to normoxia (time = 0) plotted against the level of statistical significance (q values). Different colors indicate density of data points. (C) Heatmap illustrating temporal changes between NEFs and MEFs at 1-, 2-, 3-, and 4-h time points under anoxic conditions. Genes were divided into 4 groups based on the relative expression under anoxic conditions compared with that under normoxic conditions. (D) GO terms of biological process (BP) for each cluster. X-axis represents enrichment and the size depicts the log10 (p value) of each GO term; y-axis represents the different BP terms. Data were scaled across rows before mapping to colors. (E) GO terms of molecular function (MF) for each cluster. X-axis represents enrichment and the size depicts the log10 (p value) of each GO term; y-axis represents the different BP terms. Data were scaled across rows before mapping to colors. (F) KEGG pathways for each cluster. X-axis represents enrichment and the size depicts the log10 (p value) of each pathway; y-axis represents the different KEGG pathways. Data were scaled across rows before mapping to colors. The data underlying the graphs shown in the figure can be found in S2 Data. (TIF) [file pbio.3002778.s005.tif]

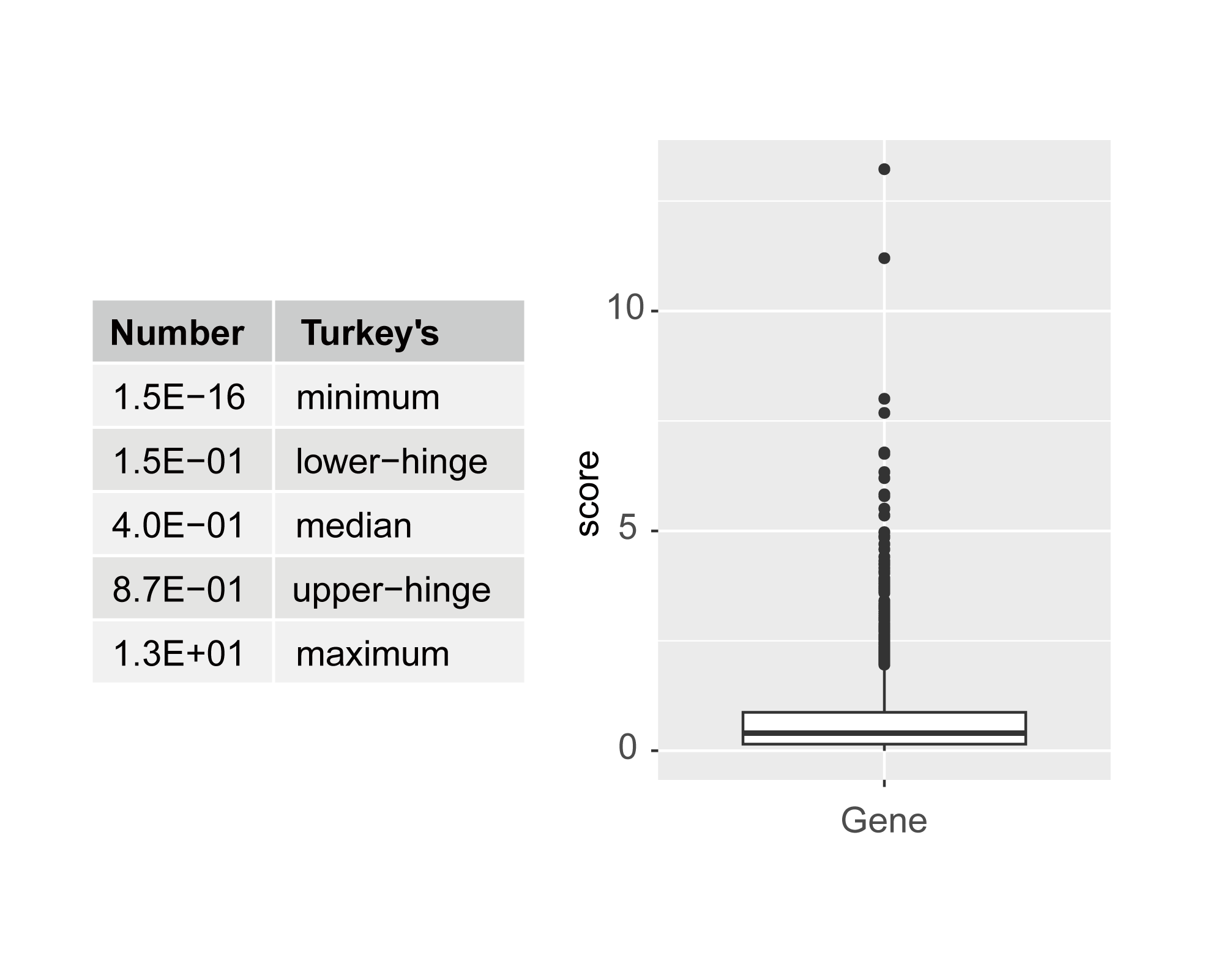

Supplement: S6 Fig — The density is plotted in x-axis and the integral is plotted on the y-axis. Box plots including Turkey’s 5 numbers for proteins according to the score (A) or the absolute integral score (B). Totally identified 667 proteins shown significant scores changes, had a score >0.87, corresponding to the 75th percentile. The data underlying the graphs shown in the figure can be found in S2 Data. (TIF) [file pbio.3002778.s006.tif]

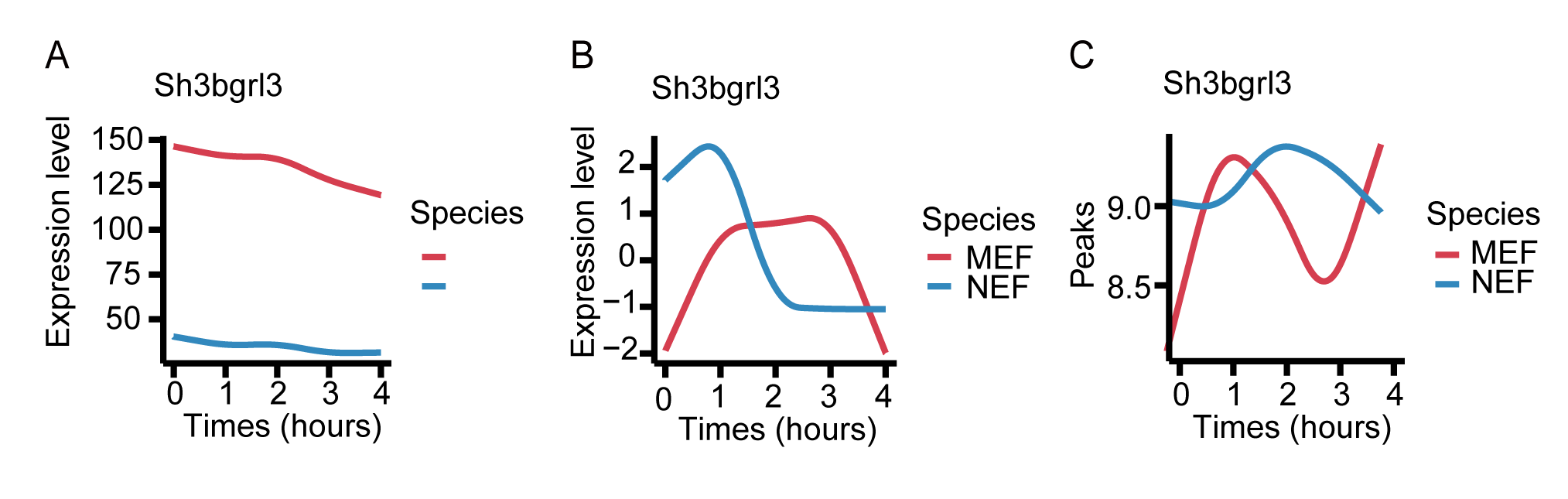

Supplement: S7 Fig — (A) RNA level of Sh3bgrl3 in NEFs and MEFs according to RNA-seq analysis. (B) Protein level of SH3BGRL3 in NEFs and MEFs according to proteomics analysis. (C) Chromatin opening peaks of Sh3bgrl3 in NEFs and MEFs according to ATAC-seq analysis. The data underlying the graphs shown in the figure can be found in S2 Data. (TIF) [file pbio.3002778.s007.tif]

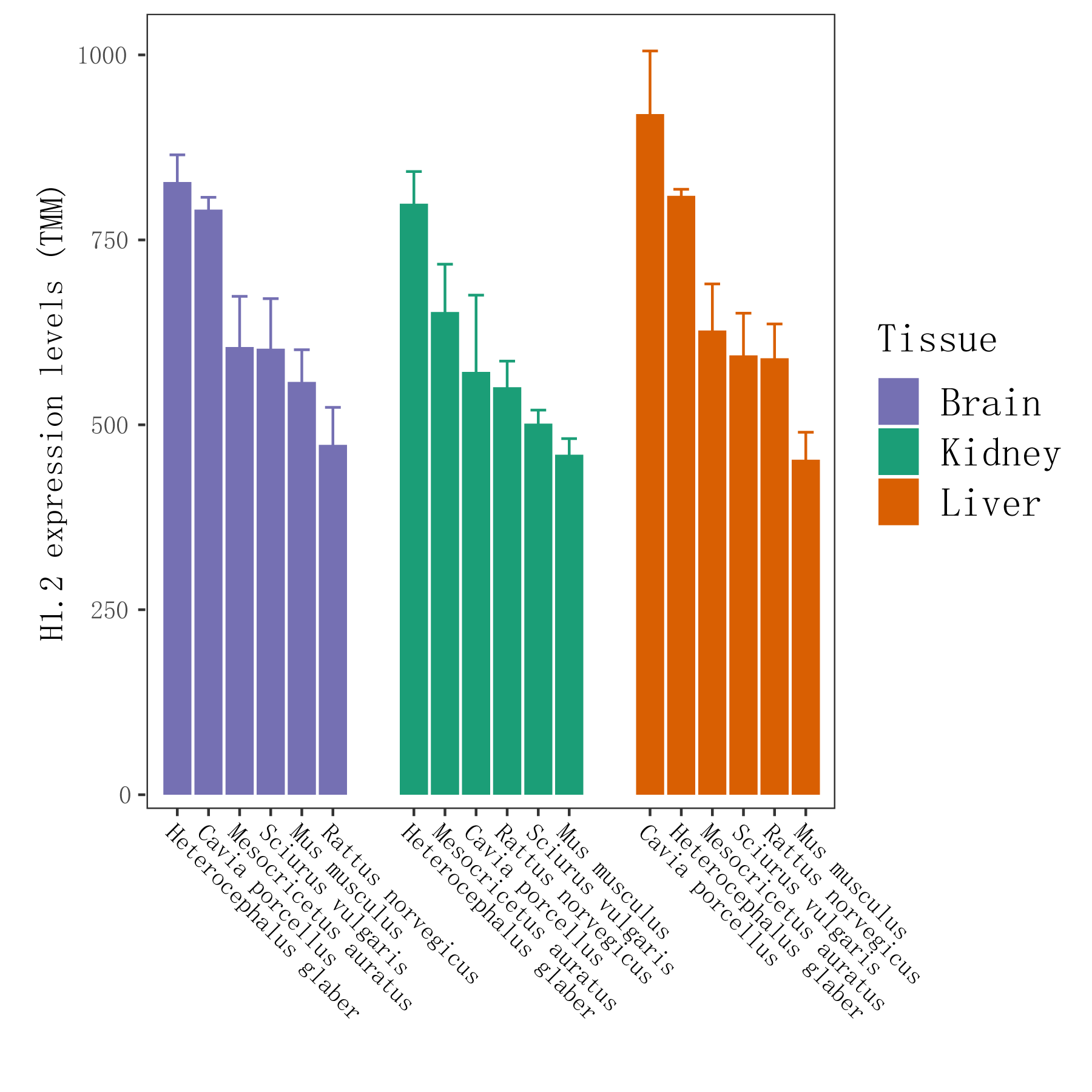

Supplement: S8 Fig — (A) H1.2 mRNA expression levels in tissues of different mammals. Brain (purple), kidney (green), and liver (orange). H1.2 were highly expressed in the liver, kidney, and brain of naked mole rats compared with other mammals. The data underlying the graphs shown in the figure can be found in S2 Data. (TIF) [file pbio.3002778.s008.tif]

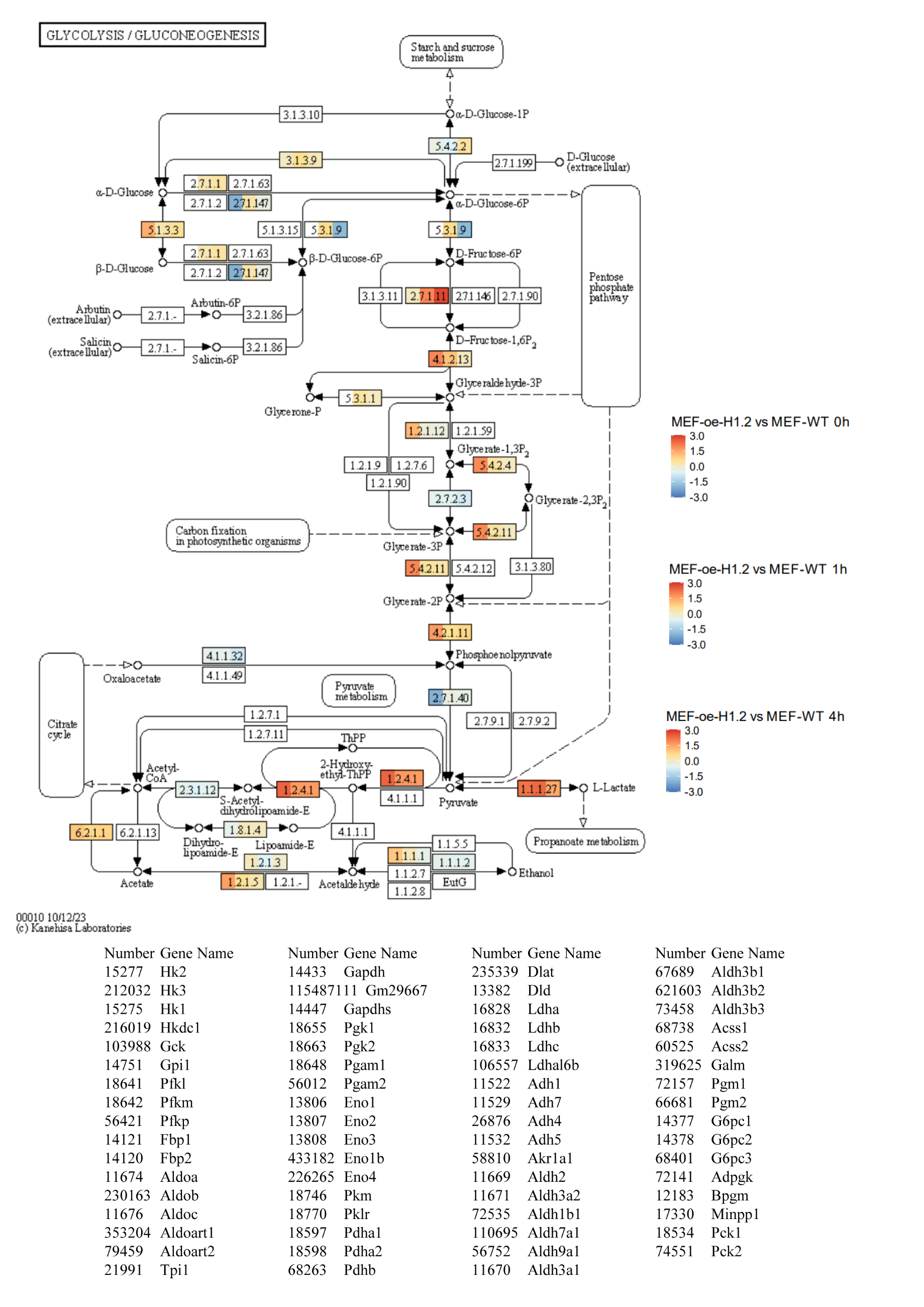

Supplement: S9 Fig — Different colors reflect the change of gene expression of MEF-oe-H1.2 compared with MEF-WT at 0, 1, and 4 h. The gene names represented by the numbers are listed at the bottom of the figure. (TIF) [file pbio.3002778.s009.tif]

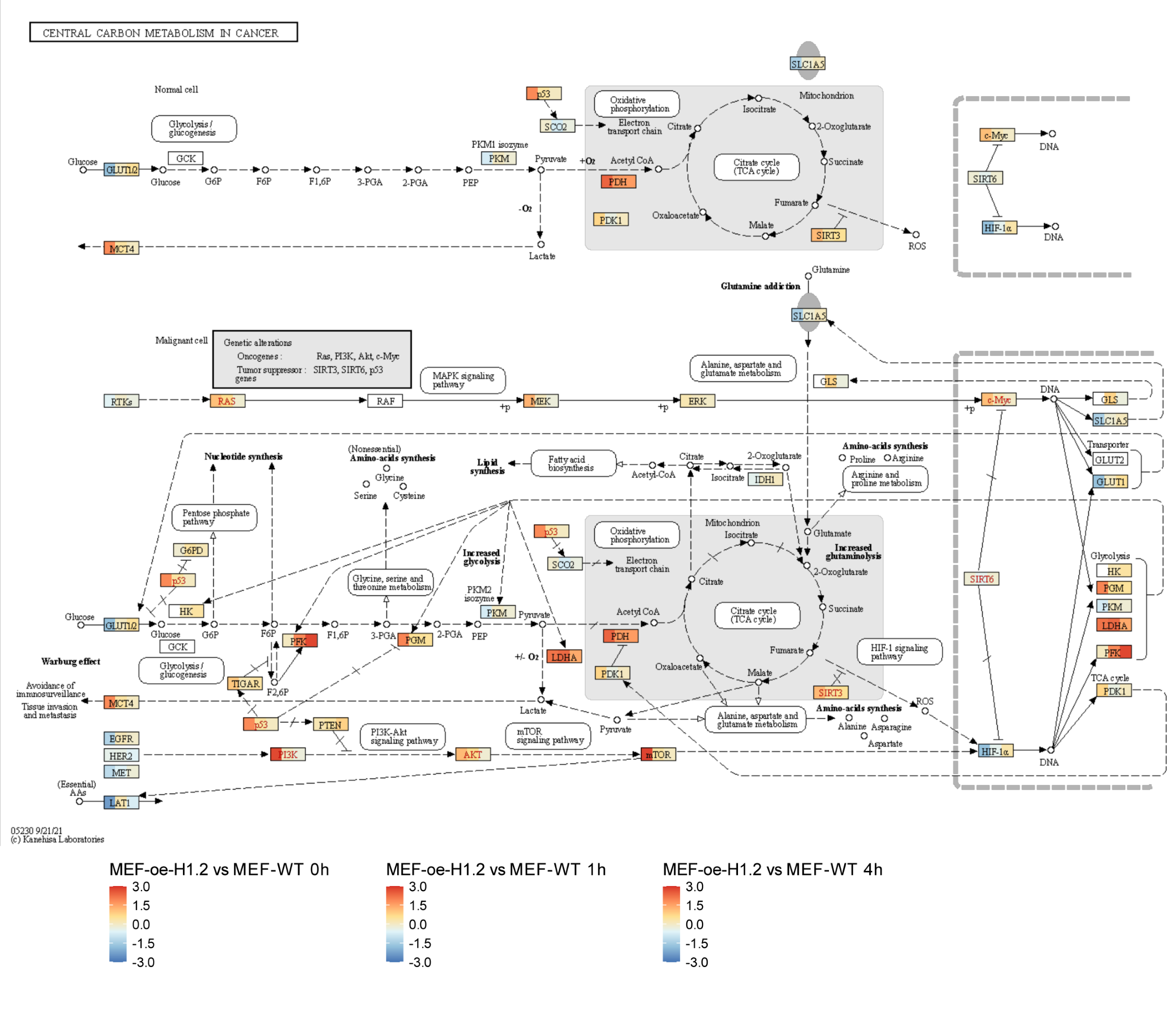

Supplement: S10 Fig — Different colors reflect the change of gene expression of MEF-oe-H1.2 compared with MEF-WT at 0, 1, and 4 h. (TIF) [file pbio.3002778.s010.tif]

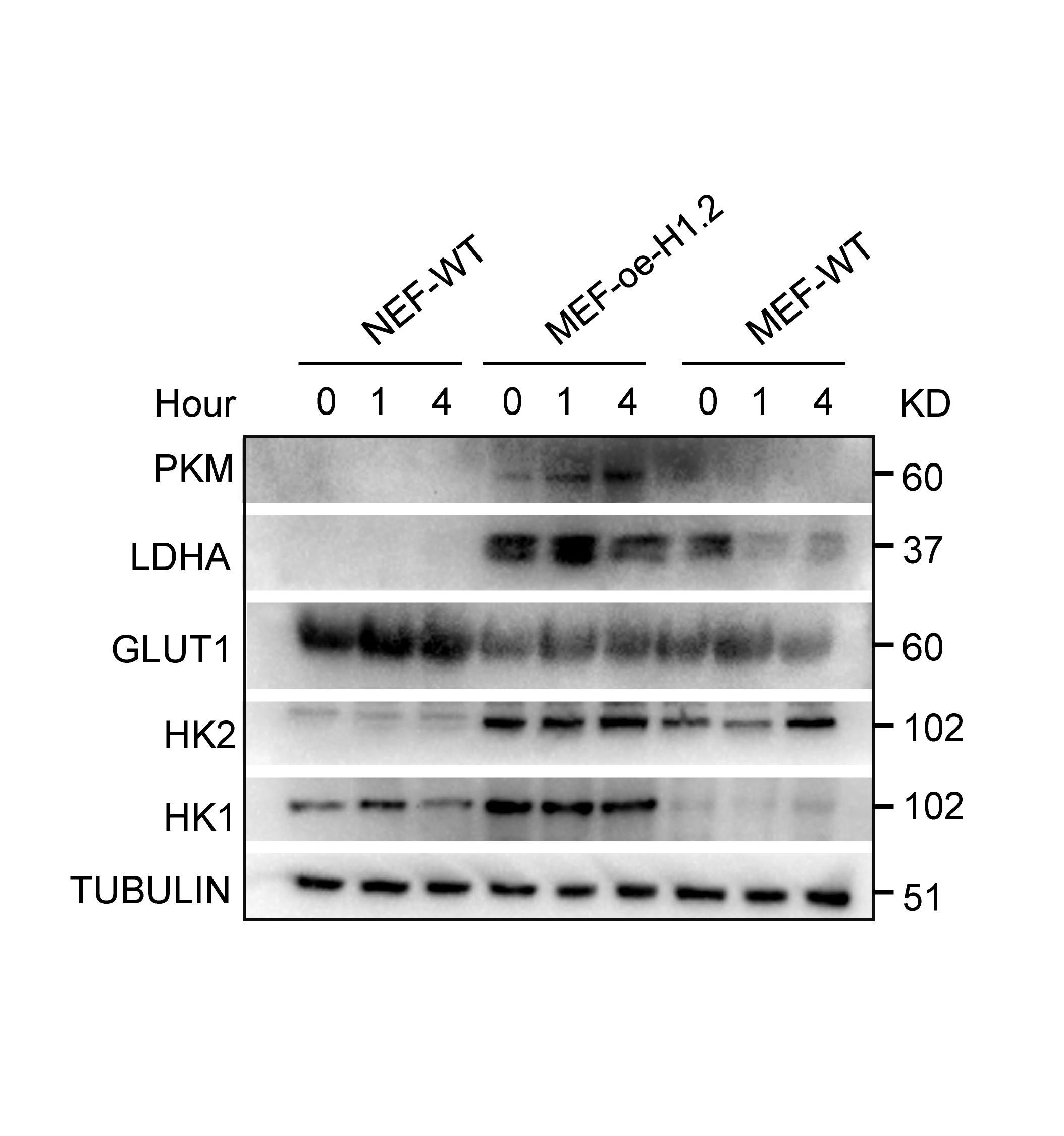

Supplement: S11 Fig — Western blot analysis of PKM, LDHA, GLUT1, HK2, and HK1 expressions in NEF, MEF-oe-H1.2, and MEF-WT cells. (TIF) [file pbio.3002778.s011.tif]

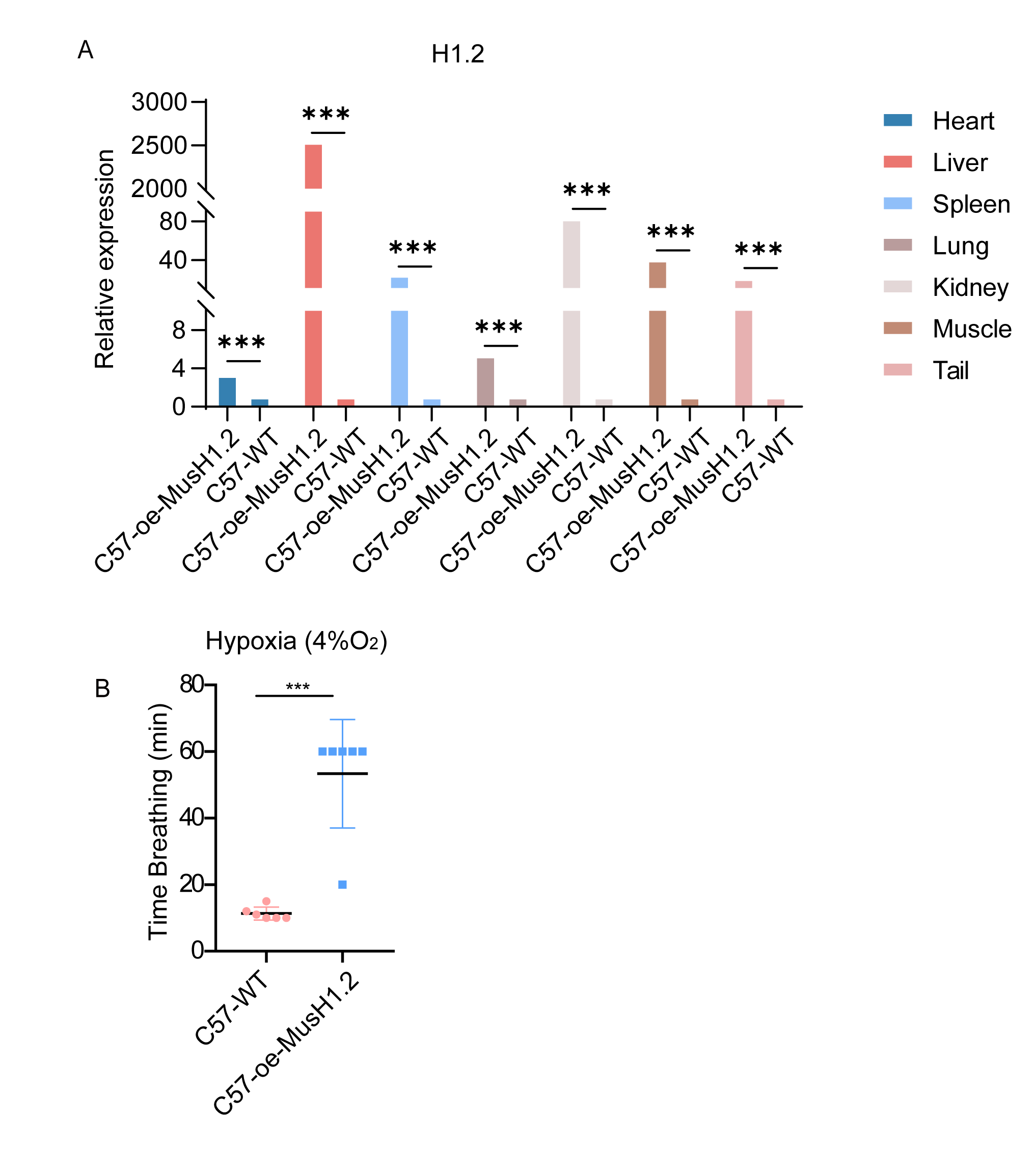

Supplement: S12 Fig — (A) RT-PCR results of H1.2 in heart, liver, spleen, lung, kidney, muscle, and tail of C57-oe-MusH1.2 and C57-WT. (B) Breathing time of WT-C57 and C57-oe-MusH1.2 male mice under 4% O2 conditions. The data underlying the graphs shown in the figure can be found in S2 Data. (TIF) [file pbio.3002778.s012.tif]

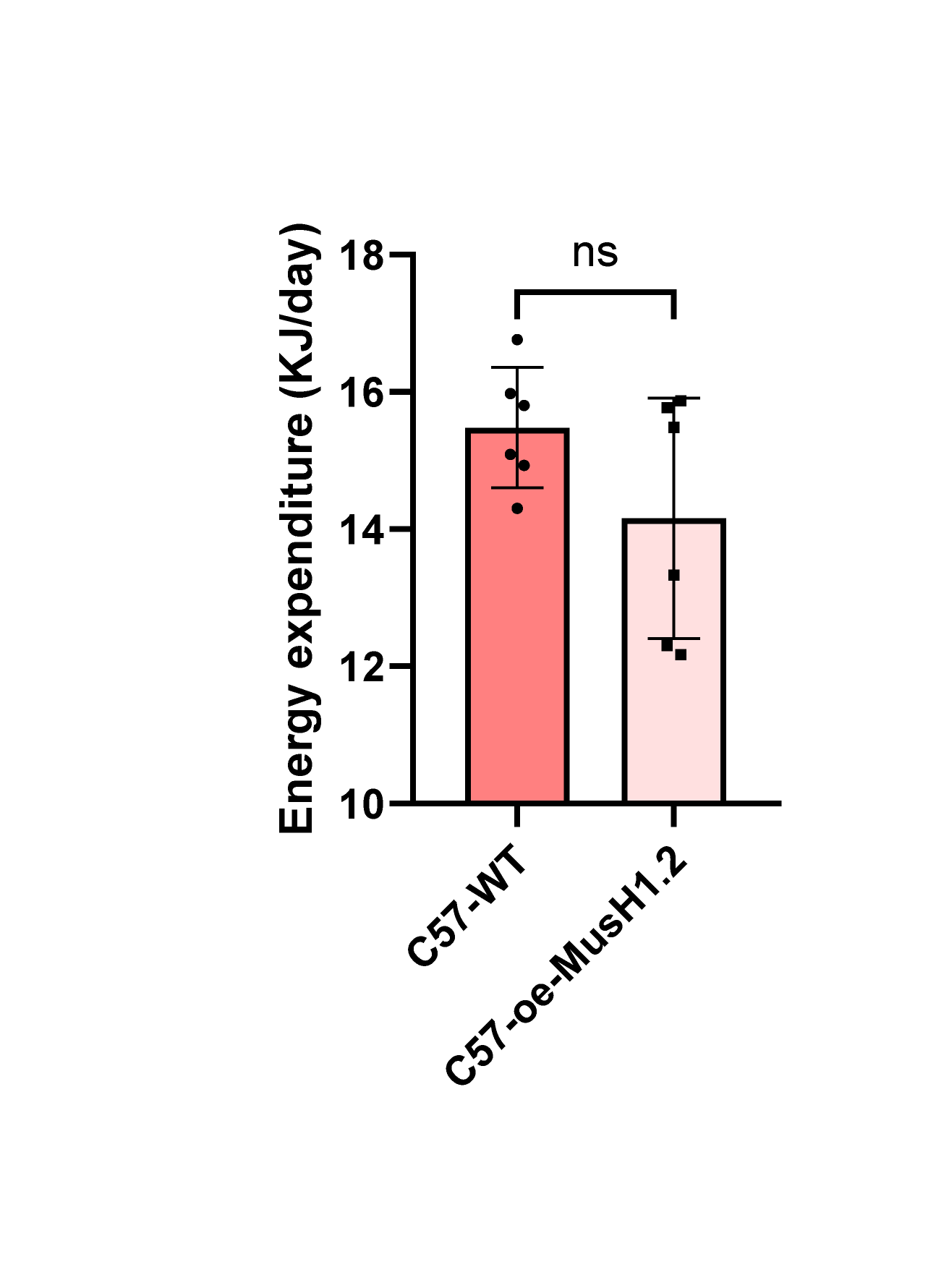

Supplement: S13 Fig — Daily energy expenditure of WT-C57 and C57-oe-MusH1.2 male mice. The data underlying the graphs shown in the figure can be found in S2 Data. (TIF) [file pbio.3002778.s013.tif]

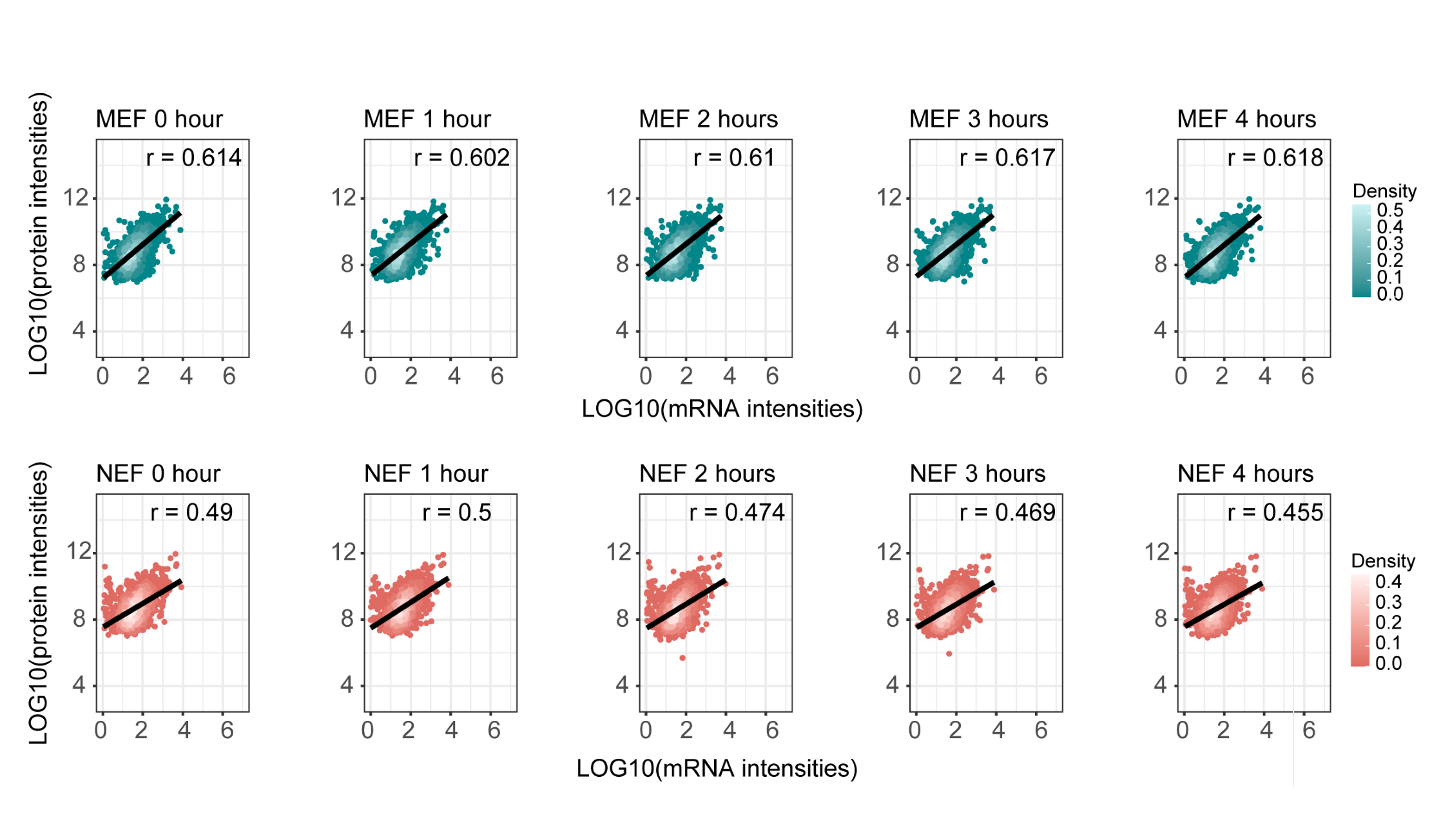

Supplement: S14 Fig — Correlation of log-transformed protein intensities and log-transformed FPKM (fragments per kilobase per million mapped reads) values (RNA intensities) from MEF and NEF for each time point. The data underlying the graphs shown in the figure can be found in S2 Data. (TIF) [file pbio.3002778.s014.tif]

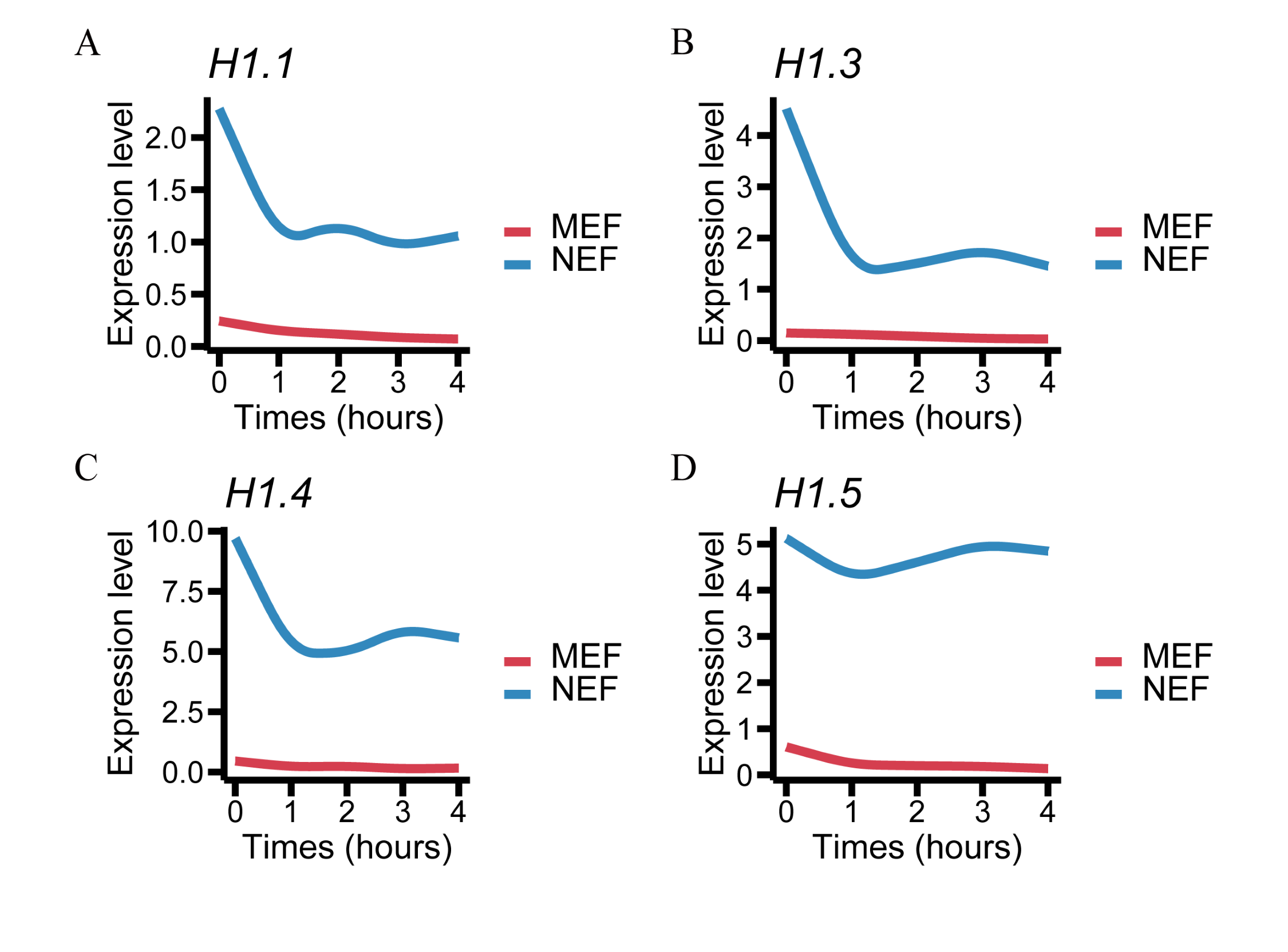

Supplement: S15 Fig — (A) RNA level of H1.1 in NEFs and MEFs according to RNA-seq analysis. Times 0 represent normoxic condition, times 1, 2, 3, 4 represent at 1, 2, 3, and 4 h time points under anoxic conditions, respectively, hereinafter inclusive. (B) RNA level of H1.3 in NEFs and MEFs according to RNA-seq analysis. (C) RNA level of H1.4 in NEFs and MEFs according to RNA-seq analysis. (D) RNA level of H1.5 in NEFs and MEFs according to RNA-seq analysis. The data underlying the graphs shown in the figure can be found in S2 Data. (TIF) [file pbio.3002778.s015.tif]

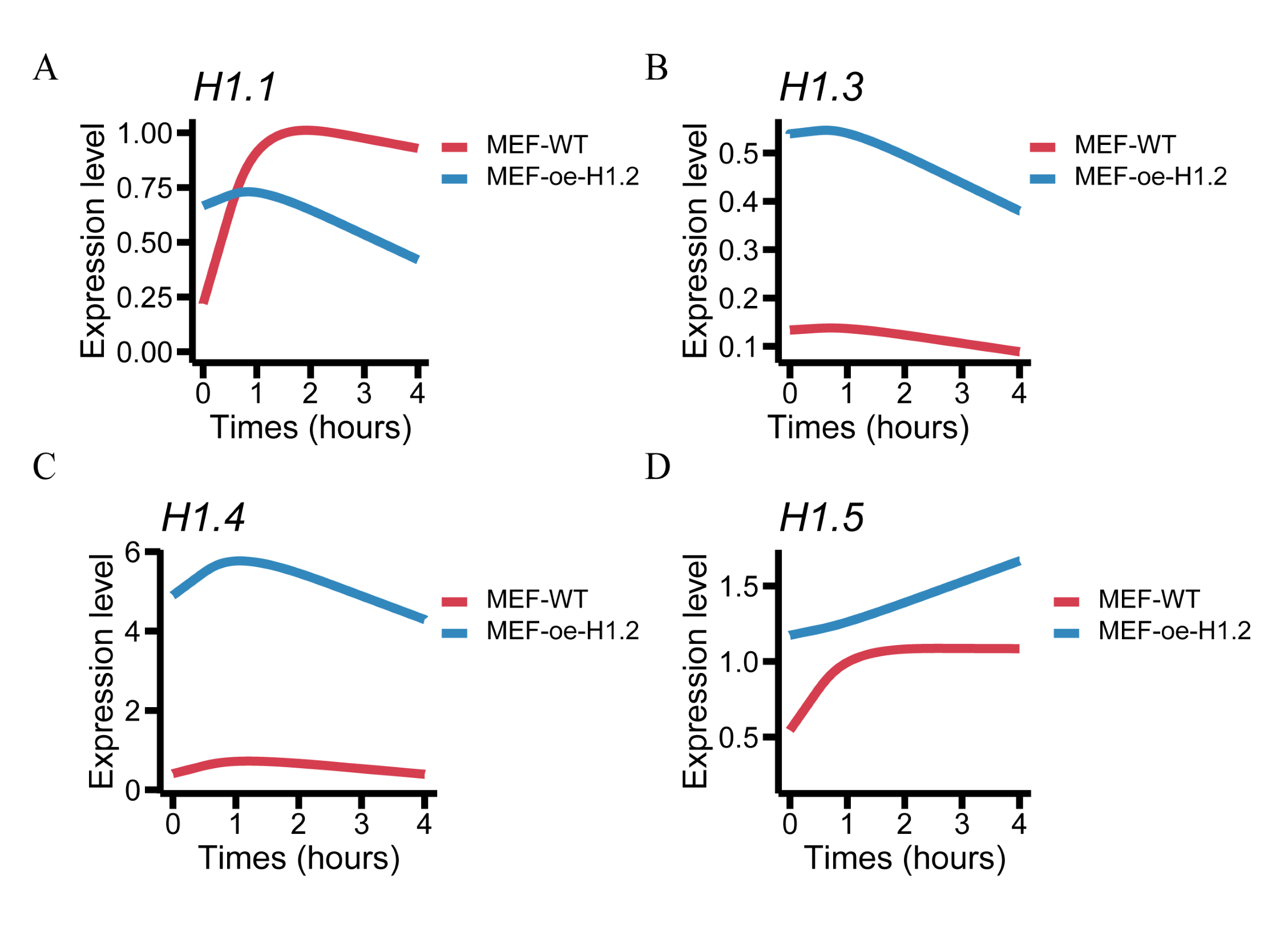

Supplement: S16 Fig — (A) RNA level of H1.1 in overexpression H1.2 MEFs and WT MEFs according to RNA-seq analysis. Times 0 represent normoxic condition, times 1, 2, 3, 4 represent at 1, 2, 3, and 4 h time points under anoxic conditions, respectively, hereinafter inclusive. (B) RNA level of H1.3 in overexpression H1.2 MEFs and WT MEFs according to RNA-seq analysis. (C) RNA level of H1.4 in overexpression H1.2 MEFs and WT MEFs according to RNA-seq analysis. (D) RNA level of H1.5 in overexpression H1.2 MEFs and WT MEFs according to RNA-seq analysis. The data underlying the graphs shown in the figure can be found in S2 Data. (TIF) [file pbio.3002778.s016.tif]

**3F**

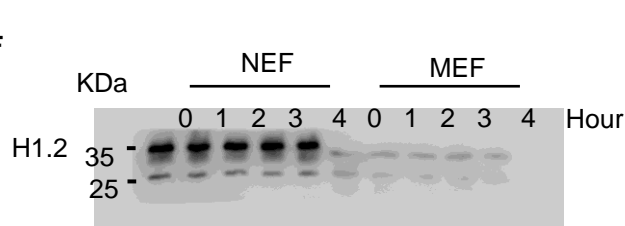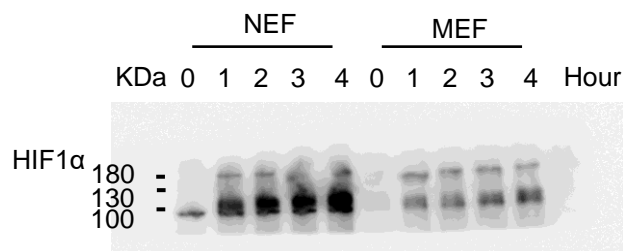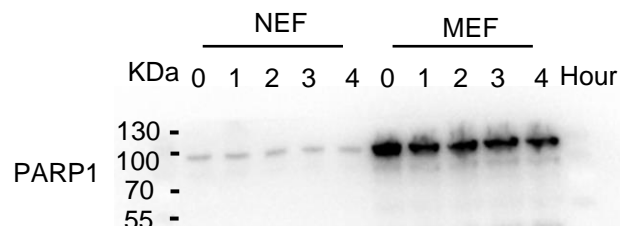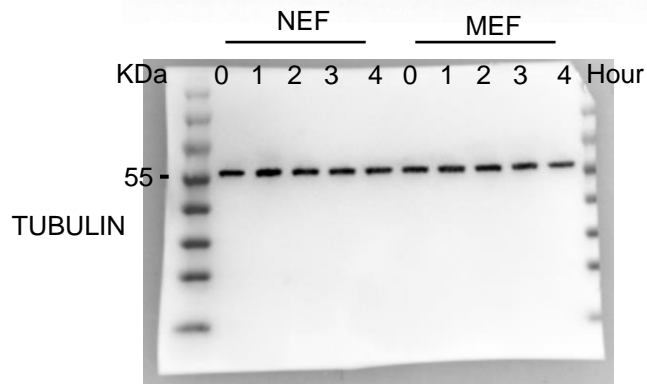

**3G**

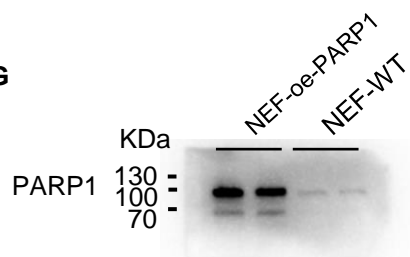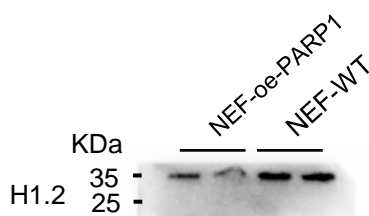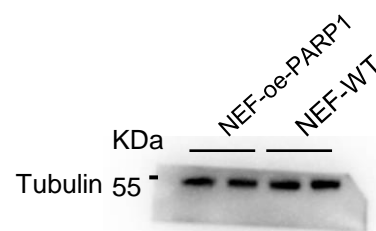

**3H**

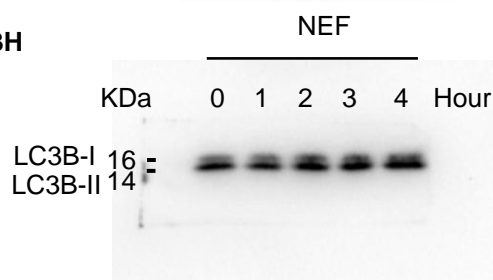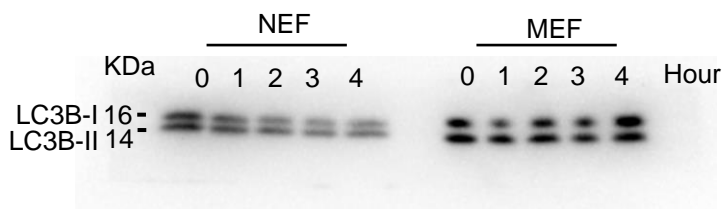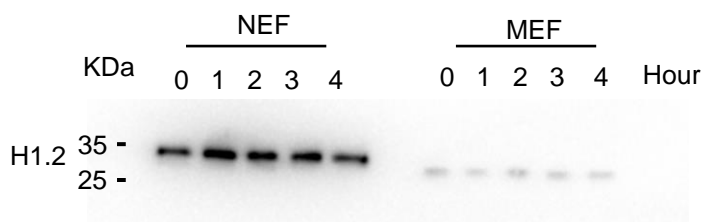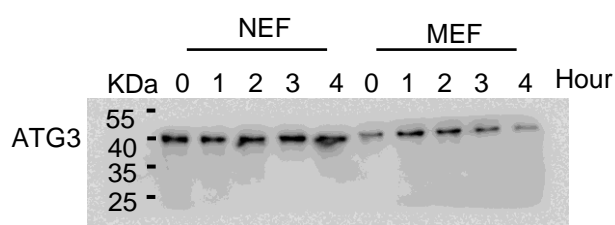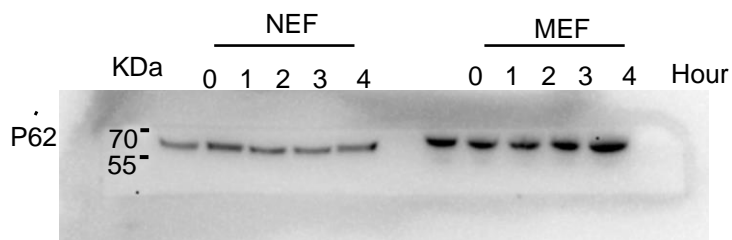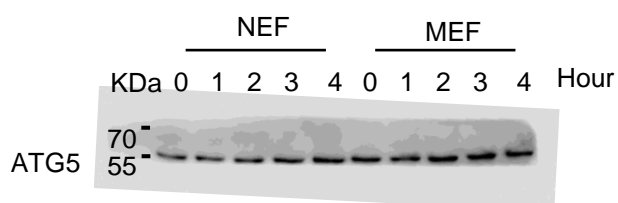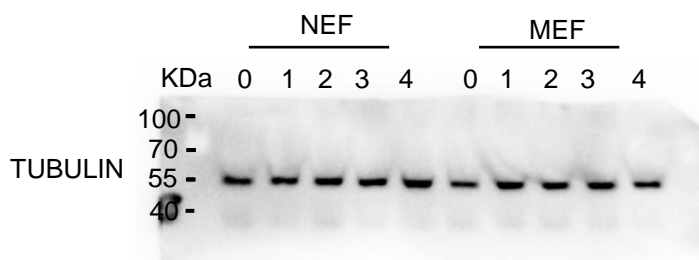

3K

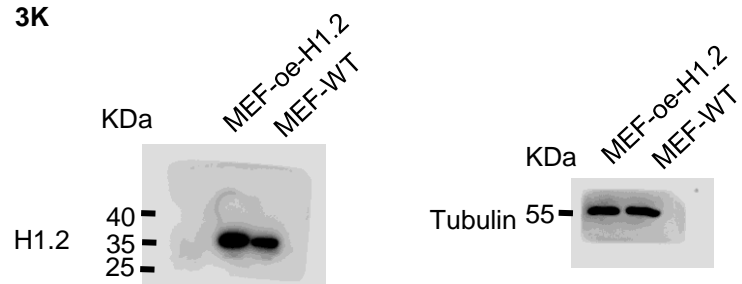

3M

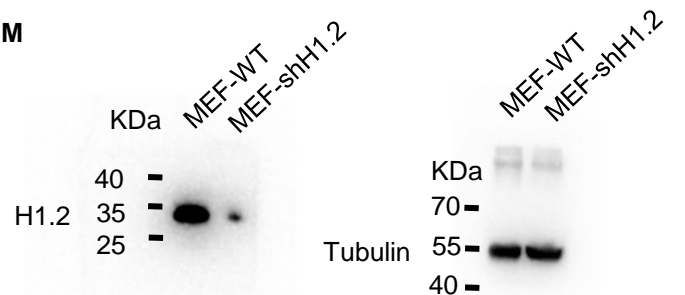

3L

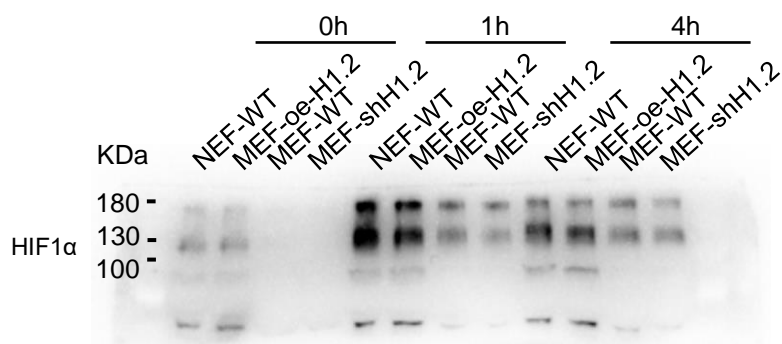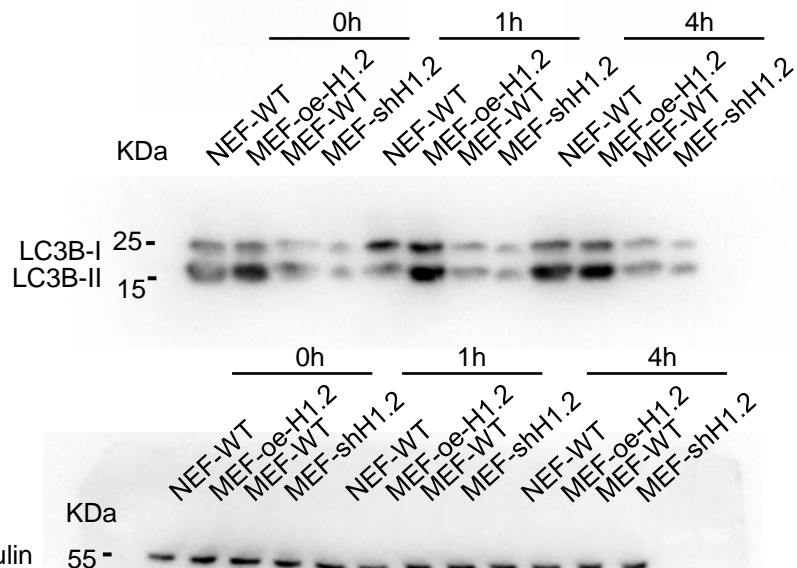

4C

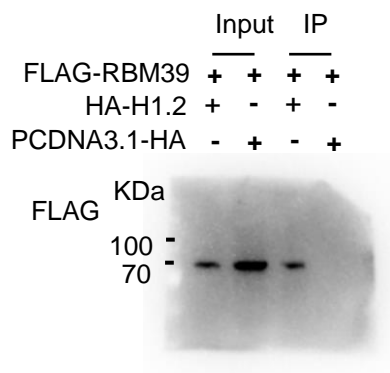

4D

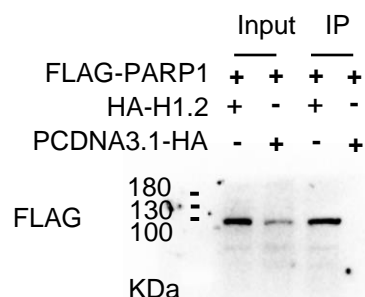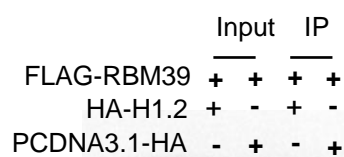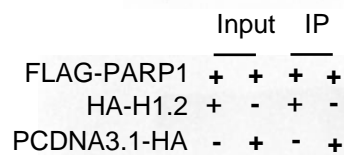

4E

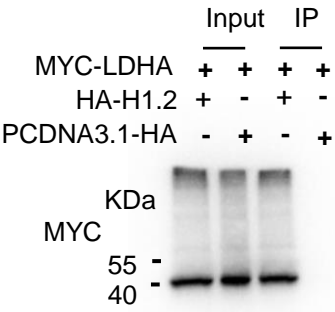

4F

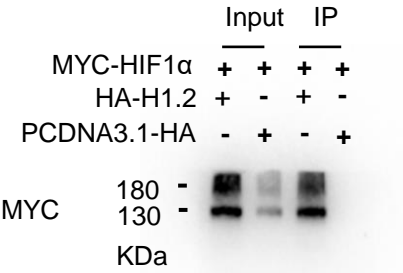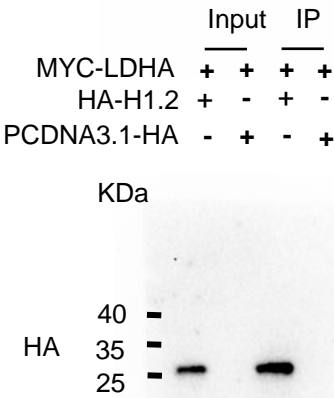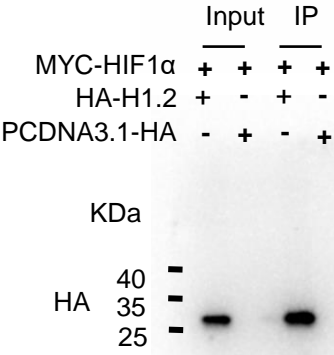

4G

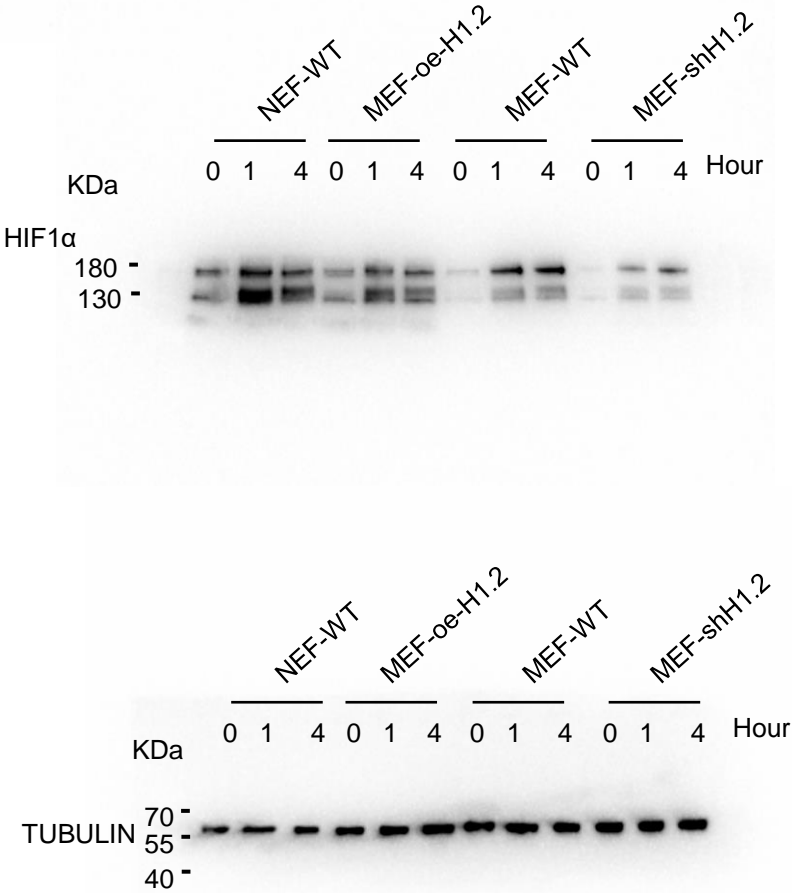

4I

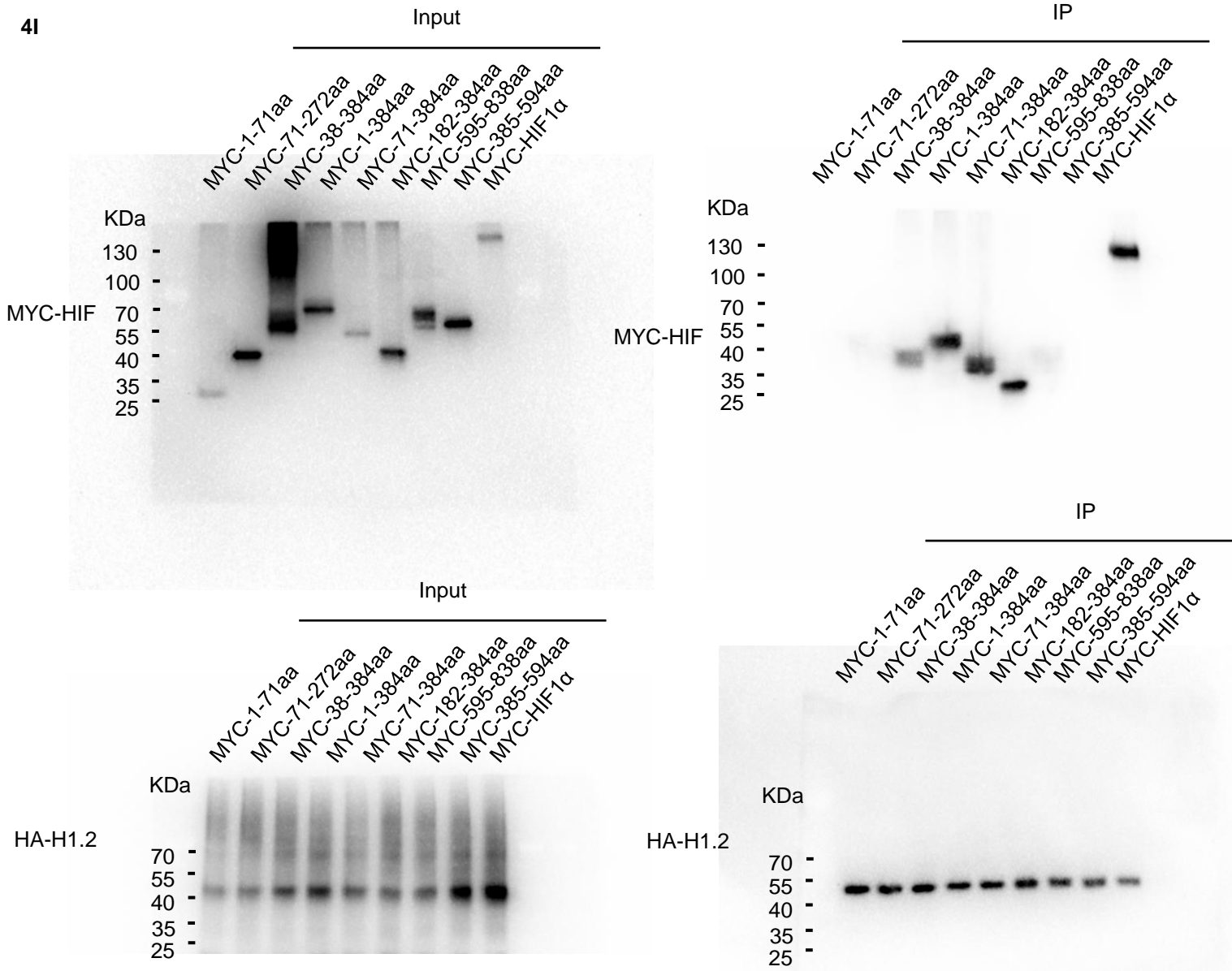

4J

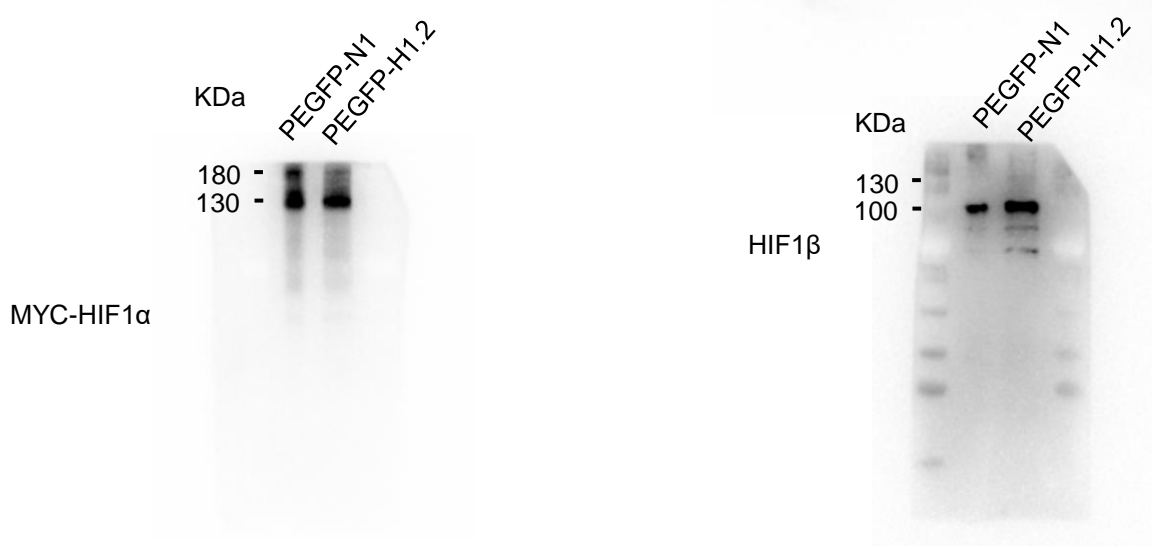

4K

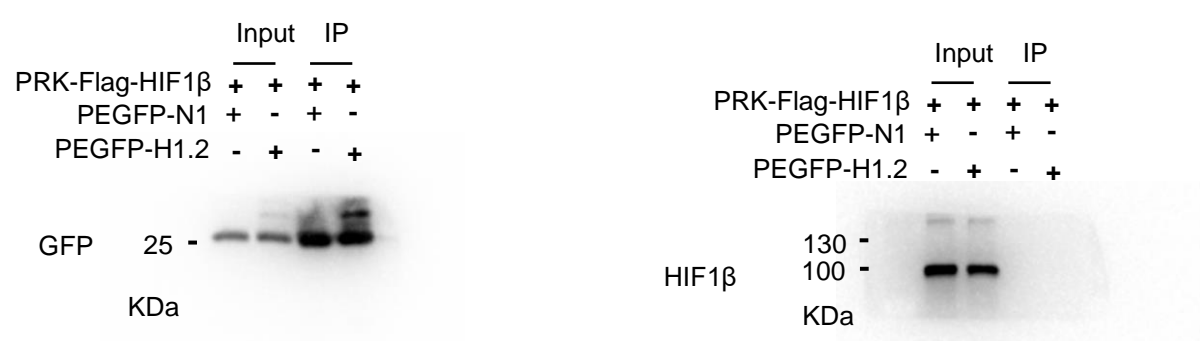

4L

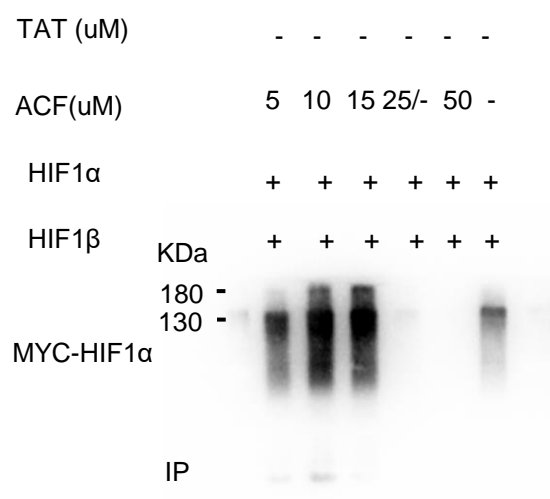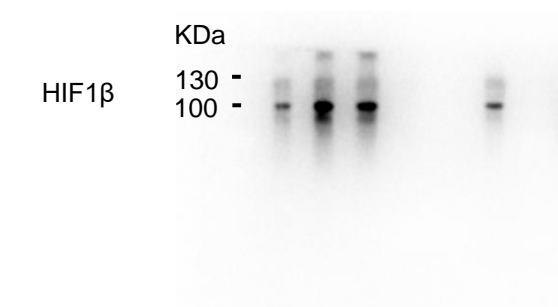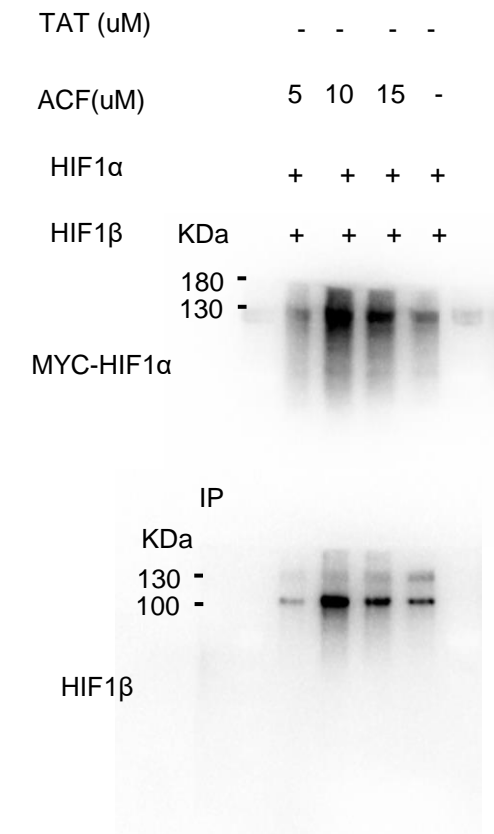

oeH1.2

oeH1.2

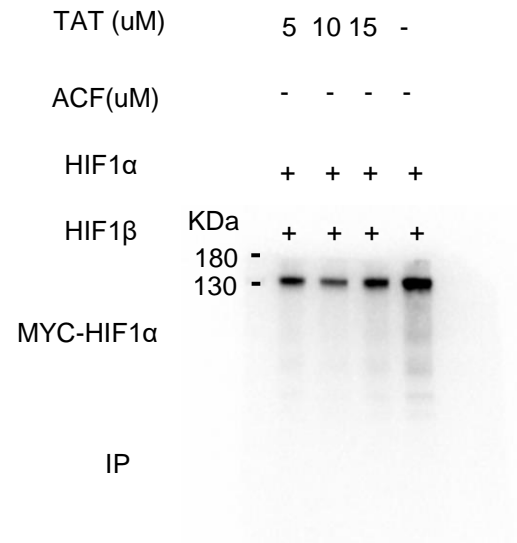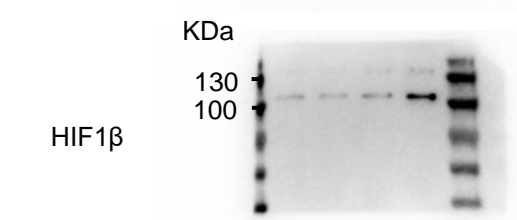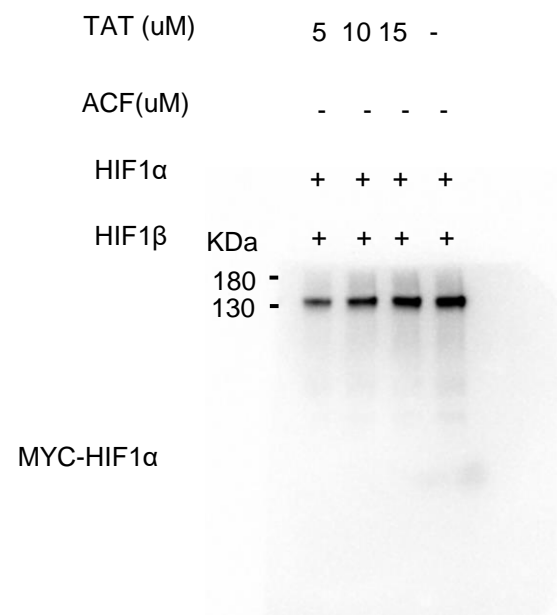

MYC-HIF1α

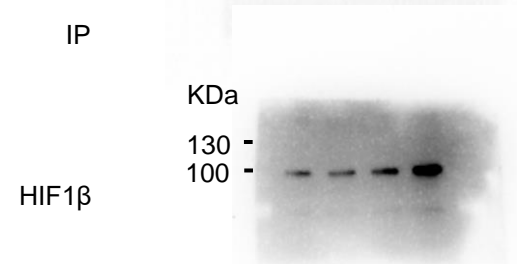

HIF1β

oeH1.2

oeH1.2

7A

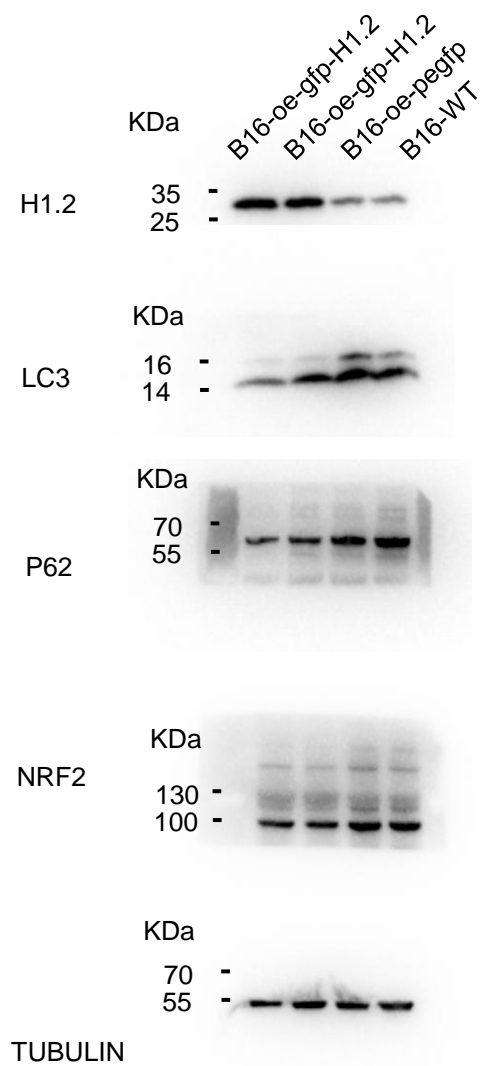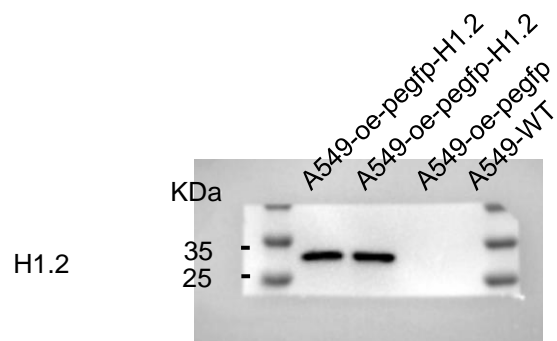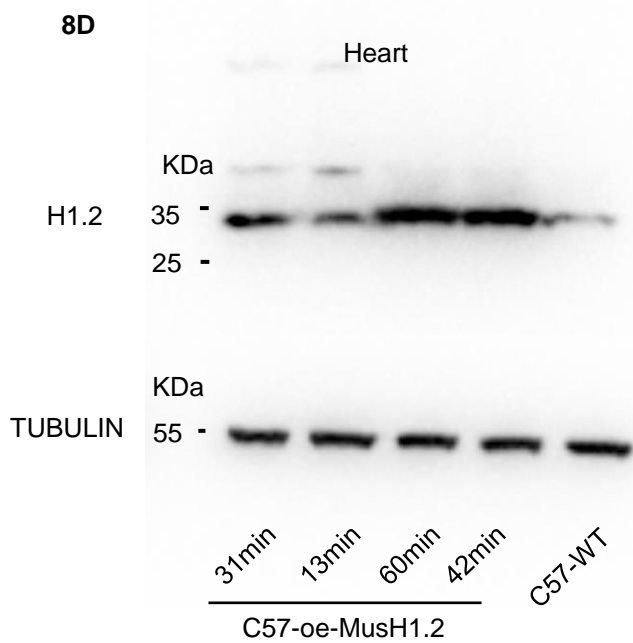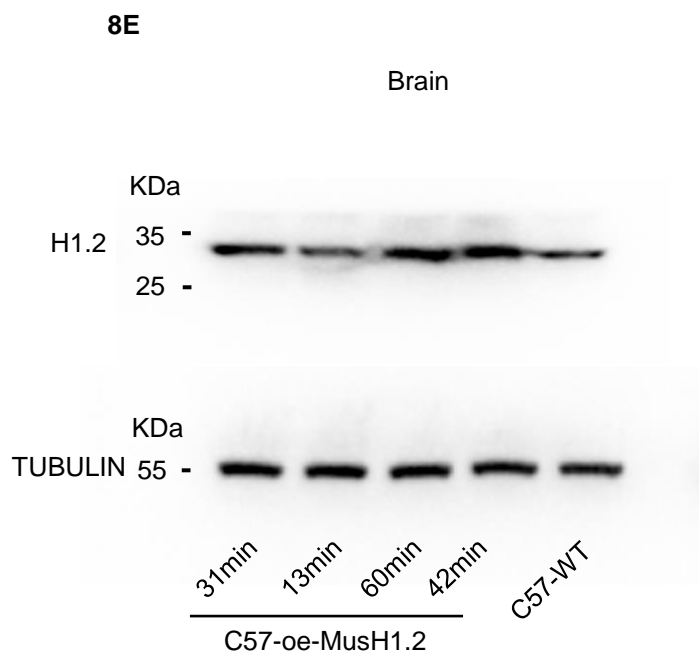

8F

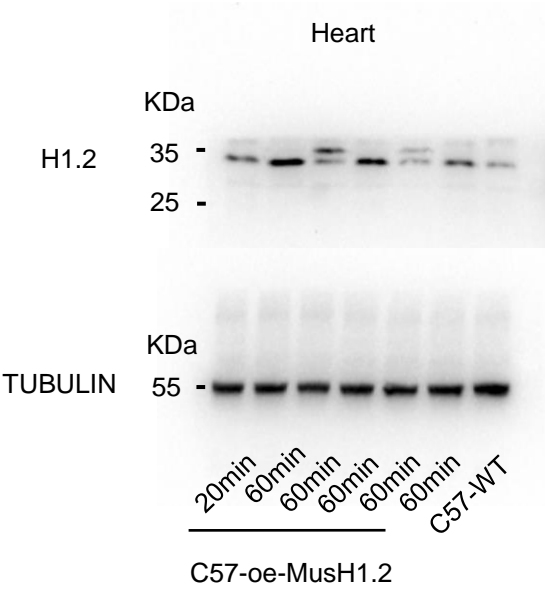

8G

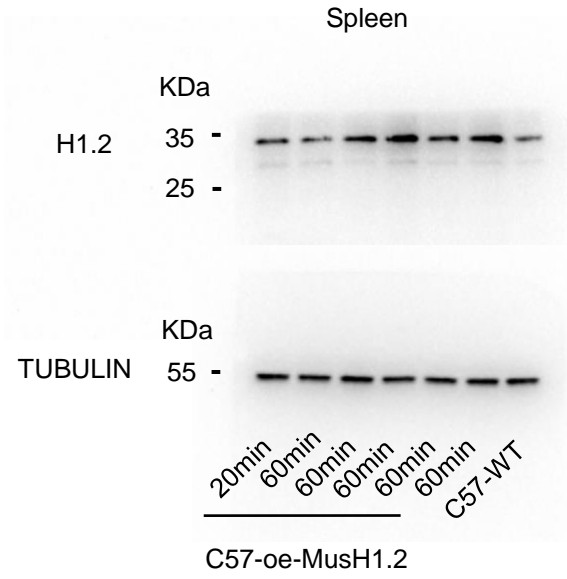

8H

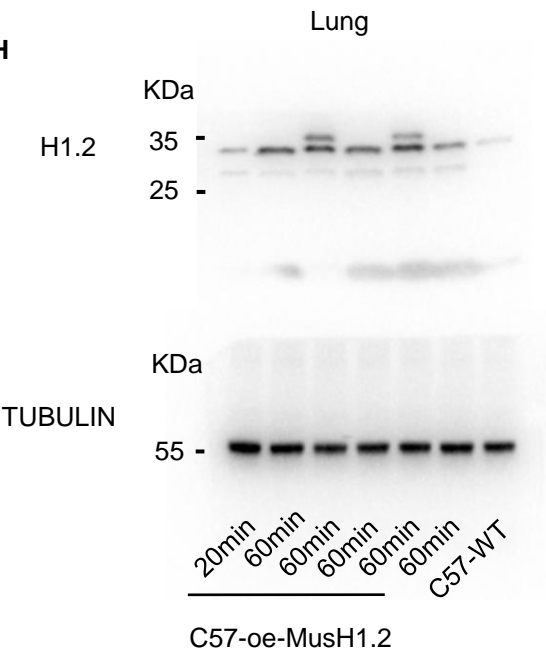

8I

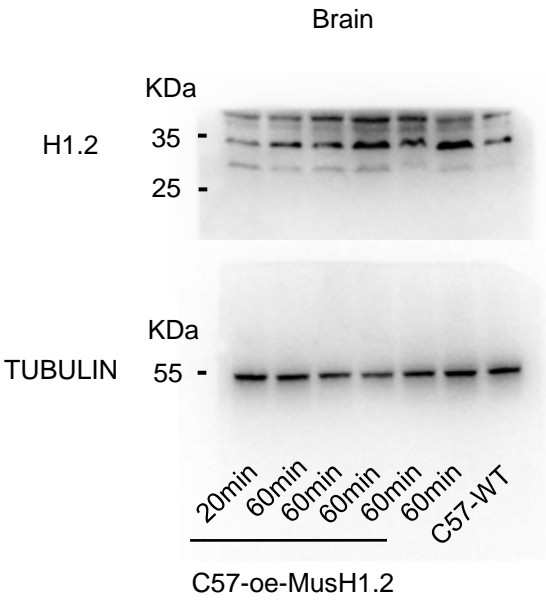

9C

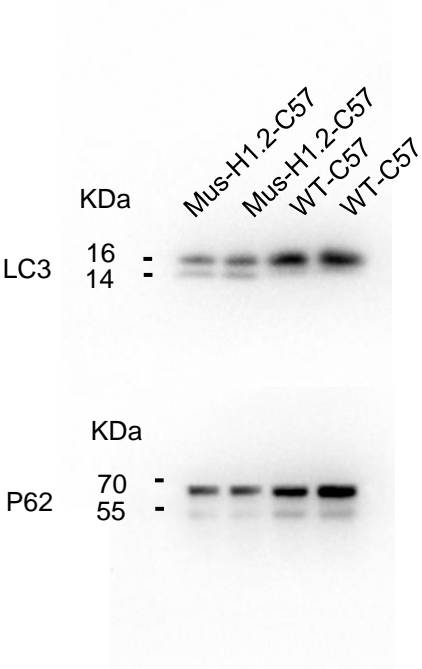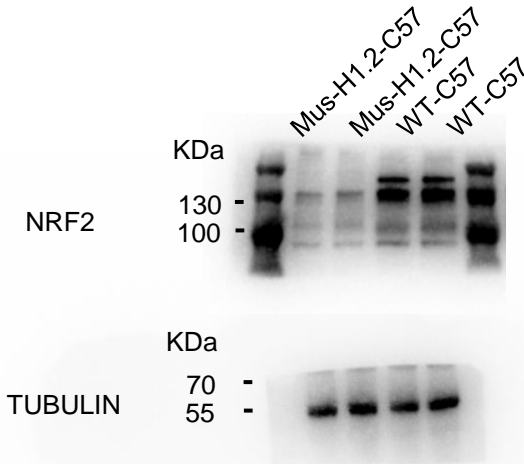

S11

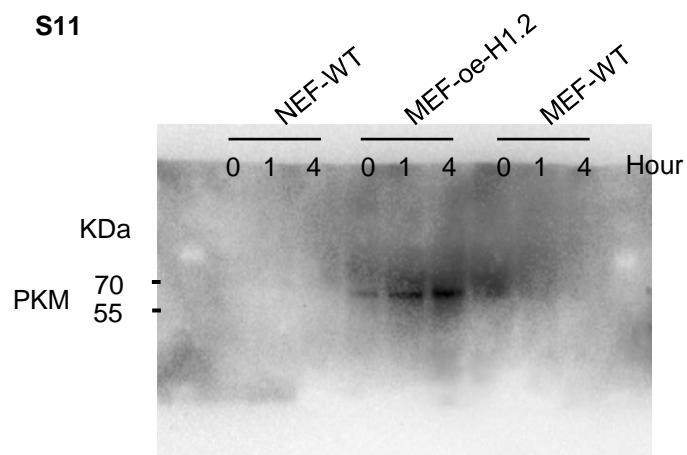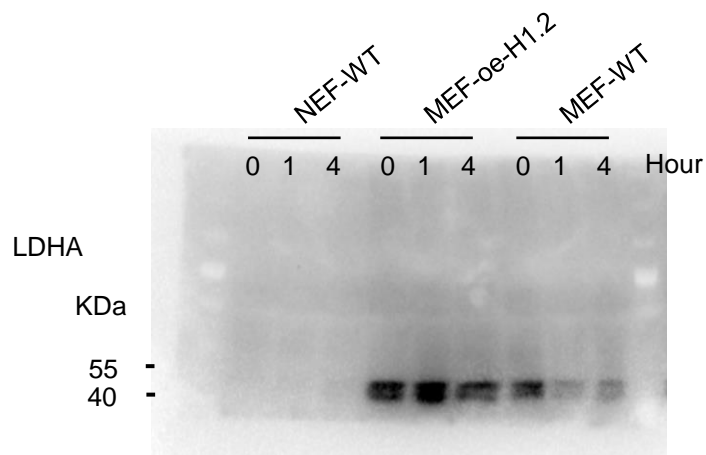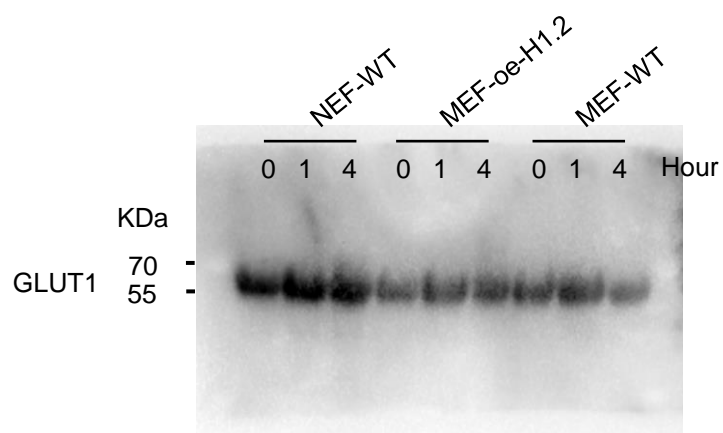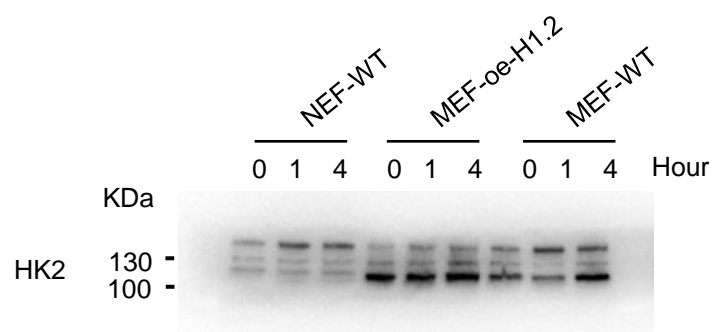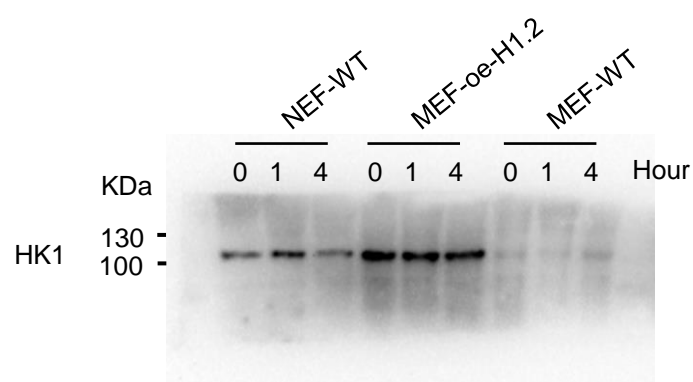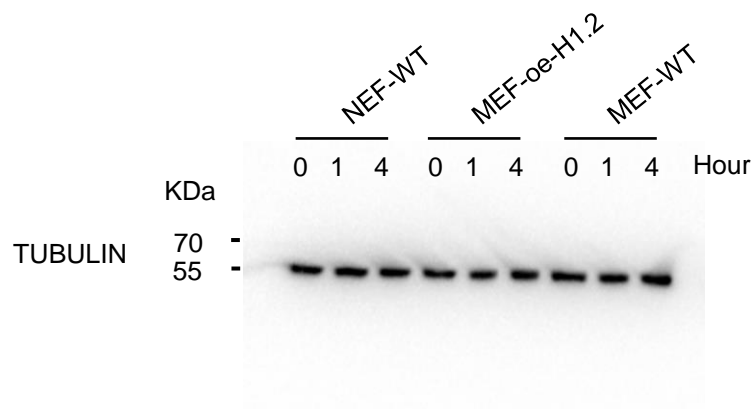

Supplement: S1 Raw Images — (PDF) [file pbio.3002778.s031.pdf]
